# Identification and single-base gene-editing functional validation of a *cis-EPO* variant as a genetic predictor for EPO-increasing therapies

**DOI:** 10.1016/j.ajhg.2022.08.004

**Published:** 2022-09-01

**Authors:** Charli E. Harlow, Josan Gandawijaya, Rosemary A. Bamford, Emily-Rose Martin, Andrew R. Wood, Peter J. van der Most, Toshiko Tanaka, Hampton L. Leonard, Amy S. Etheridge, Federico Innocenti, Robin N. Beaumont, Jessica Tyrrell, Mike A. Nalls, Eleanor M. Simonsick, Pranav S. Garimella, Eric J. Shiroma, Niek Verweij, Peter van der Meer, Ron T. Gansevoort, Harold Snieder, Paul J. Gallins, Dereje D. Jima, Fred Wright, Yi-hui Zhou, Luigi Ferrucci, Stefania Bandinelli, Dena G. Hernandez, Pim van der Harst, Vickas V. Patel, Dawn M. Waterworth, Audrey Y. Chu, Asami Oguro-Ando, Timothy M. Frayling

**Affiliations:** 1 University of Exeter Medical School, University of Exeter, Royal Devon and Exeter NHS Trust, Exeter EX2 5DW, UK; 2 University of Groningen, University Medical Center Groningen, Department of Epidemiology, Groningen 9713, the Netherlands; 3 Longitudinal Studies Section, Translation Gerontology Branch, National Institute on Aging, Baltimore, MD 21224, USA; 4 Laboratory of Neurogenetics, National Institute on Aging, NIH, Bethesda, MD 20892, USA; 5 Data Tecnica International, Glen Echo, MD 20812, USA; 6 Center for Alzheimer’s and Related Dementias, National Institutes of Health, Bethesda, MD 20892, USA; 7 Eshelman School of Pharmacy and Center for Pharmacogenomics and Individualized Therapy, University of North Carolina at Chapel Hill, 120 Mason Farm Road, Chapel Hill, NC 27599, USA; 8 AbbVie Inc., 1000 Gateway Boulevard, South San Francisco, CA 94080, USA; 9 Division of Nephrology-Hypertension, University of California San Diego, San Diego, CA, USA; 10 Laboratory of Epidemiology and Population Sciences, National Institute on Aging, Bethesda, MD 20892, USA; 11 University of Groningen, University Medical Center Groningen, Department of Cardiology, Groningen 9713, the Netherlands; 12 University of Groningen, University Medical Center Groningen, Department of Nephrology, Groningen 9713, the Netherlands; 13 Bioinformatics Research Center, North Carolina State University, 1 Lampe Drive, Raleigh, NC 27695, USA; 14 Center for Human Health and the Environment, North Carolina State University, Raleigh, NC 27606, USA; 15 Geriatric Unit, Azienda Sanitaria Firenze, Florence 50134, Italy; 16 Department of Cardiology, University Medical Center Utrecht, Utrecht 3584, the Netherlands; 17 GlaxoSmithKline, Collegeville, PA 19426, USA; 18 GlaxoSmithKline, Boston, MA 02140, USA

**Keywords:** anaemia in chronic kidney disease, mendelian randomization, drug-target mendelian randomization, gene editing, functional genomics, CRISPR-Cas9, functional validation, statistical genetics, population studies, genome-wide association study, cardiovascular disease risk

## Abstract

Hypoxia-inducible factor prolyl hydroxylase inhibitors (HIF-PHIs) are currently under clinical development for treating anemia in chronic kidney disease (CKD), but it is important to monitor their cardiovascular safety. Genetic variants can be used as predictors to help inform the potential risk of adverse effects associated with drug treatments. We therefore aimed to use human genetics to help assess the risk of adverse cardiovascular events associated with therapeutically altered EPO levels to help inform clinical trials studying the safety of HIF-PHIs. By performing a genome-wide association meta-analysis of EPO (n = 6,127), we identified a *cis-EPO* variant (rs1617640) lying in the *EPO* promoter region. We validated this variant as most likely causal in controlling EPO levels by using genetic and functional approaches, including single-base gene editing. Using this variant as a partial predictor for therapeutic modulation of EPO and large genome-wide association data in Mendelian randomization tests, we found no evidence (at p < 0.05) that genetically predicted long-term rises in endogenous EPO, equivalent to a 2.2-unit increase, increased risk of coronary artery disease (CAD, OR [95% CI] = 1.01 [0.93, 1.07]), myocardial infarction (MI, OR [95% CI] = 0.99 [0.87, 1.15]), or stroke (OR [95% CI] = 0.97 [0.87, 1.07]). We could exclude increased odds of 1.15 for cardiovascular disease for a 2.2-unit EPO increase. A combination of genetic and functional studies provides a powerful approach to investigate the potential therapeutic profile of EPO-increasing therapies for treating anemia in CKD.

## Graphical abstract

**Figure:**
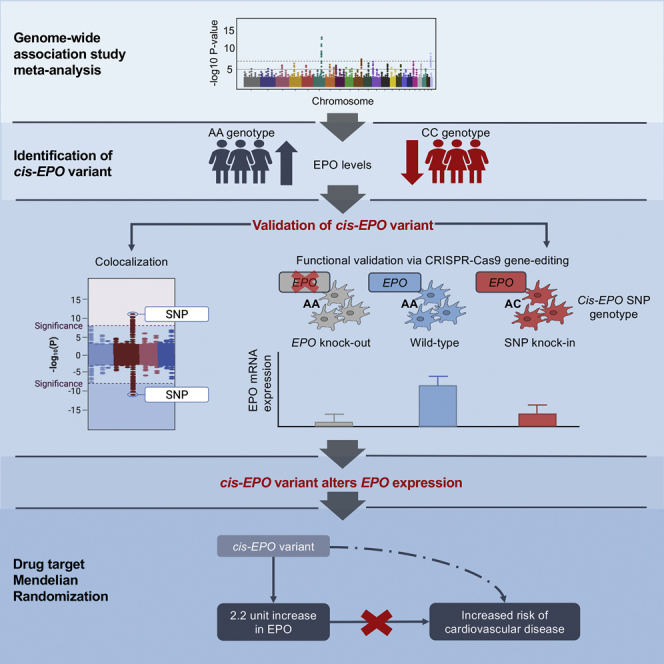

## Teaser

We identified a common genetic variant associated with EPO levels by performing a GWAS. We successfully validated the genetic variant as causal by using gene-editing techniques. Finally, we used the variant as a partial predictor for EPO-raising therapies and found no evidence of adverse cardiovascular events with long-term EPO rises.

## Introduction

Anemia, one of the primary complications of chronic kidney disease (CKD), affects one out of every seven individuals with CKD.^1^, ^2^, ^3^ Anemia is associated with faster progression of CKD and increased risk of adverse events, particularly heart disease or stroke, two of the major causes of death associated with CKD.^4^^,^^5^ Current therapies used to treat anemia in CKD include blood transfusions, intravenous iron therapies, or parenteral injections of recombinant erythropoietin (rhEPO). These treatments have safety and compliance concerns, including risk of infection, adverse gastro-intestinal effects, and increased risk of stroke, myocardial infarction (MI), venous thromboembolism, and heart failure.^4^^,^^6^, ^7^, ^8^, ^9^, ^10^, ^11^, ^12^ These safety concerns have led to the development of hypoxia-inducible factor (HIF) prolyl hydroxylase inhibitors (PHIs) as a novel class of treatment for anemia in CKD. PHIs inhibit the prolyl hydroxylase enzymes (PHD1-3) allowing the level of HIF-1 to rise and bind to the hypoxia response element that, among other activities, increases the transcription of *EPO* and in turn endogenous EPO levels.^13^^,^^14^ EPO stimulates the bone marrow to increase the production of red blood cells and circulating hemoglobin (Hgb) levels.^12^ The recent completion of phase III clinical trials assessing cardiovascular safety has indicated non-inferiority of PHIs compared to rhEPO and shown that PHIs can increase and maintain Hgb levels with small increases in circulating EPO levels compared to exogenous EPO administration.^4^^,^^15^, ^16^, ^17^, ^18^, ^19^, ^20^, ^21^, ^22^, ^23^ PHIs have already received approval for clinical use in Japan, supporting ongoing development elsewhere.^11^^,^^15^^,^^18^^,^^24^, ^25^, ^26^

To further assess the potential side-effects of EPO-based treatments, we used a genetic approach. Several studies have shown that genetic data can provide supporting evidence of an association between the drug target and intended therapeutic indication. Genetic associations can also help identify potential unintended effects and inform potential drug safety profiles.^27^, ^28^, ^29^, ^30^, ^31^, ^32^, ^33^ Genetic variants lying within or nearby the gene encoding the drug target are more likely to have functional impact on the protein product than those further away in the genome.^34^ These variants can be used in Mendelian randomization (MR) tests as partial proxies for pharmacological action to help inform the effects of long-term modulation of drug targets on disease outcomes.^35^, ^36^, ^37^ The properties of inherited genetic variation mean the associations are far less likely to be confounded or biased, compared to observational studies. Functional studies can provide further evidence that a genetic variant is a valid proxy for a drug target, for example, by confirming that the variant controls the expression of the expected gene. The development of CRISPR-Cas9 gene editing has made functional validation more straightforward by establishing whole-gene knockouts and modifying single-nucleotide polymorphisms (SNPs).^38^, ^39^, ^40^

This study aimed to use human genetics to examine the long-term effect of genetically predicted therapeutic modulation of endogenous EPO levels. First, we identified a genetic variant lying *in cis* with *EPO* that is associated with endogenous circulating EPO levels. Second, we functionally validated that variant by using CRISPR-Cas9 gene-editing techniques. Third, we used this variant in drug-target MR tests as a genetic predictor for therapeutically altered EPO levels to help inform the long-term effects of elevated endogenous EPO levels on risk of cardiovascular disease (CVD) or clinical markers (blood pressure and resting heart rate) predisposing to CVD risk factors (e.g., hypertension).

## Material and methods

The steps performed to identify and functionally validate a genetic variant associated with circulating EPO are outlined in Figure S1 and, briefly, are as follows.

### Identification of genetic variants associated with circulating EPO levels

To identify genetic variants associated with circulating EPO levels, we performed a genome-wide association study (GWAS) meta-analysis of 6,127 individuals of European and African descent from four independent cohorts: InCHIANTI (n = 1,210), PREVEND (n = 2,954), BLSA (n = 458), and HealthABC (n = 1,505) (Table S1). Details of phenotype generation and inclusion criteria are described in the supplemental material and methods, and an analysis plan is outlined in Figure S2. Each study was approved by an institutional review board as described in the supplemental material and methods. We inverse normalized EPO levels to account for a skewed distribution and performed GWAS in GEMMA^41^ by using an additive linear mixed model adjusting for age, sex, and any study-specific covariates alongside a genomic relationship matrix to account for all types of relatedness. We combined study-specific estimates and performed inverse variance-weighted fixed-effects meta-analysis on ∼25.1 million imputed SNPs in 6,127 unrelated individuals of European and African descent by using METAL^42^ with the following filters: minor allele count (MAC) > 3, effect allele frequency (EAF) > 1, EAF < 0, Info ≥ 0.3. After performing meta-analysis, we excluded SNPs with a minor allele frequency (MAF) < 0.01 and performed a multi-SNP-based step-wise conditional and joint association analysis by using GCTA-COJO^43^^,^^44^ to select SNPs independently associated with EPO (p < 5 × 10^−8^).

### Identification and validation of *cis-EPO* genetic variant

To identify a genetic variant most likely to directly impact on the protein product for use as a genetic predictor for therapeutically altered endogenous EPO levels, we analyzed the GWAS data around *EPO* specifically to identify any *cis-*acting genetic variants and selected variants previously identified. We converted the genetic effect estimate (in SDs) of the genetic variant to original units (IU/L) by using the standard deviation from the InCHIANTI study (Table S2).

### Expression quantitative trait loci (eQTLs) analysis

Having identified a *cis-EPO* variant (rs1617640) associated (p = 9.32 × 10^−4^) with circulating EPO protein levels, we tested its *cis*-effects (+/− 500 kb) with gene expression in a meta-analysis of hepatic gene expression from 861 livers from European individuals in three datasets^45^ (Table S3) and 236 kidneys from 134 individuals in one renal gene expression dataset^46^ (Table S4). Additional study details can be found in supplemental material and methods. We selected the liver and kidney, as *EPO* is highly expressed in both tissues.

### Colocalization analysis

We performed colocalization analysis to assess the likelihood that the liver *EPO* eQTL was the same signal as the circulating protein level association. We obtained summary data for hepatic *cis*-eQTLs associated with *EPO* expression (false discovery rate [FDR] < 0.1) 500 kb on either side of rs1617640 and extracted the summary statistics for these SNPs from our circulating EPO meta-analysis. We performed approximate Bayes factor colocalization analyses by using the R coloc package.^47^^,^^48^ We obtained overall estimates of the posterior probability that both our EPO meta-analysis and the liver eQTL share the same causal variant.

### Establishment of whole-*EPO* knockout

With the use of CRISPR-Cas9 gene-editing technology, human embryonic kidney (HEK)-293 cells were subjected to gene editing to generate isogenic *EPO* knockout (*EPO*^−/−^) cell lines. In brief, paired guide-RNAs (gRNAs), one targeting exon 2 (Ensembl: ENST00000252723.3) (5′-AGAGGTACCTCTCCAGGACT**CGG**-3′) and one targeting exon 4 (5′-CATGTGGATAAAGCCGTCAG**TGG**-3′), were separately cloned into the CRISPR-Cas9 expression vector containing a green fluorescent protein (GFP) reporter (pSpCas9(BB)-2A-GFP (PX458), Addgene: #48138) and an mCherry fluorescent protein reporter (pU6-(BbsI) CBh-Cas9-T2A-mCherry, Addgene: #64324), respectively. 1 × 10^6^ HEK-293 cells were seeded in 10 cm plates and 24 h later were co-transfected with 6 μg of each CRISPR-Cas9/gRNA vector using Lipofectamine LTX reagent (Thermo Fisher Scientific, Massachusetts, USA). Successfully transfected GFP and red fluorescent protein-positive cells were manually isolated under the EVOS FLoid Imaging system (Thermo Fisher Scientific, Massachusetts, USA). Single cells were clonally expanded for around 2 weeks and media containing 10% FBS was replaced every 48 h. Single cells were screened via polymerase chain reaction (PCR) assay using primers on either side of the two gRNAs (forward: 5′-TCTAGAATGTCCTGCCTGGC-3′, reverse: 5′-GGCCCTGTGACATCCTTAGA-3′). Sanger sequencing was used to confirm successful disruption to *EPO*. All wild-type (WT) HEK-293 cells were treated the same throughout the experiments but treated with empty CRISPR-Cas9 plasmids (i.e., containing no gRNAs).

### *EPO* overexpression

For use as a positive control*,* wild-type HEK-293 cells were transfected with an *EPO* overexpression (hEPO) construct (Addgene: #139057)^49^ using Lipofectamine LTX reagent (Thermo Fisher Scientific, Massachusetts, USA).

### RNA extraction and qRT-PCR

Total RNA was isolated and purified from *EPO*^−/−^ and WT HEK-293 cell lines using the Direct-zol RNA Miniprep kit following manufacturers protocol (Cambridge Biosciences, Cambridge, UK). 500 ng of RNA was converted to cDNA using PrimeScript RT reagent kit (Takara Bio Europe SAS, Saint-Germain-en-Laye, France). Quantitative reverse transcription (qRT)-PCR was performed using Hot FIREPol EvaGreen qPCR Master Mix with ROX (Solis BioDyne, Teaduspargi, Estonia) with the QuantStudio 6 Flex qPCR machine (Thermo Fisher Scientific, Massachusetts, USA) on at least three biological replicates. Primer sequences are listed in Table S5. Samples with Ct values > 2 SD from the mean were removed. Gene expression levels were standardized against the reference gene *GAPDH* messenger RNA (mRNA) levels using the 2^−ΔΔCt^ method.^50^ Expression of alternative housekeeping genes (*UBC* and *Pol2ra*) was also assessed. RefFinder^51^ was used to determine the most stable gene or combination of genes for use as an endogenous control (Table S6). Differences in gene expression levels between WT and *EPO*^−/−^ cell lines were investigated for statistical significance by a paired t test carried out in RStudio version 3.6.1.^52^

### RNA sequencing (RNA-seq)

After RNA extraction, RNA concentration and quality were assessed using the Qubit 4 Fluorometer (Thermo Fisher Scientific, Massachusetts, USA) and the Agilent 2200 TapeStation System, respectively (Agilent Technologies, California, USA). 1,000 ng of RNA from samples with an RNA integrity (RIN) score > 8 were prepared for RNA-seq.^53^ Library preparation and sequencing were performed by the Exeter Sequencing Service, resulting in 75 bp paired-end sequencing (additional details in supplemental material and methods). Quality control checks were undertaken on the raw reads using MultiQC.^54^ Adapter sequences (defined by Illumina), nucleotides with poor quality from the 3′ end (Phred < 25), and reads shorter than 25 bp were removed using CutAdapt version 1.13^55^ (Figure S3). Reads were aligned to the *Homo sapiens* GRCh38/hg38 reference genome using STAR version 2.7.1^56^ (Figure S4) and gene quantification was performed using the *featureCounts* subread package^57^ based on Ensembl GRCh38/hg38 annotation release version 2.0.0 (Figure S5). All other analysis was performed in RStudio unless otherwise stated.^52^ Transcripts whose mean count across all samples were <10 were removed. Counts were normalized and transformed using the “*rlogTransformation*” function implemented in *DeSeq2*.^58^ Principal-component analysis (PCA) was performed using the R “*prcomp*” function to check for similarity between samples. To identify genotype-specific gene expression changes, we performed differential gene expression analysis by using *DeSeq2*.^58^ p values were calculated using the Wald test, and a Benjamini-Hochberg correction was applied to account for multiple testing. Statistically significant differentially expressed genes (DEGs) were determined by an adjusted p value ≤ 0.05. We determined strong differential expression when genes were regulated by 2-fold. As we performed RNA-seq on two *EPO*^*−/−*^ cell lines (KOA and KOB) to obtain the most accurate list of DEGs most likely to be differentially expressed due to the effect of *EPO*^−/−^, differential expression analysis was performed comparing WT to each knockout cell line (WT versus KOA and WT versus KOB). DEGs (p ≤ 0.05) overlapping in both comparisons were identified using the R “*venn.diagram*” function. Gene set enrichment analysis was performed on this list of overlapping DEGs using Enrichr.^59^, ^60^, ^61^ Top DEGs were subsequently subjected to qRT-PCR to validate differential expression.

### Immunoblot analysis

*EPO*^−/−^, WT controls, and HEK-293 cells transfected with hEPO overexpression vector^49^ were subjected to immunoblot analysis, as described previously,^62^ using a monoclonal mouse anti-EPO antibody (1:1,000; MAB2871, R&D systems, Abingdon, UK)^63^ and a mouse anti-GAPDH antibody (1:1,000; sc-47724, Santa Cruz Biotechnology, Texas, USA). Goat anti-mouse IgG (H + L) Cross-Absorbed Alexa Fluor 680 (1:5,000; A21057, Invitrogen, Massachusetts, USA), was used as the secondary antibody. Membranes were visualized on the LI-COR Odyssey CLx system (LI-COR Biotechnologies, Nebraska, USA). Images were converted to grayscale using Image Studio Lite version 5.2.5 (LI-COR Biotechnologies, Nebraska, USA).

### Knockin of the rs1617640 C allele via CRISPR-Cas9 gene editing with the *piggyBac* transposon system

Using CRISPR-Cas9 and the *piggyBac* system, HEK-293 cells were subjected to single-base gene editing to generate isogenic cell-lines with either the A/A or A/C genotype for rs16167640 (the A allele being associated with higher circulating EPO levels) using a previously described protocol.^64^ In brief, a single gRNA (5′-GGAATCTCACTCCTCTGGCTCAGGG-3′) was cloned into the GFP CRISPR-Cas9 expression vector. In addition, 5′ and 3′ homology arms were designed (supplemental material and methods), one containing the desired base edit, and cloned into the *piggyBac* multivector (SGK:005, MV-PGK-Puro-TK, Hera BioLabs, Kentucky, USA) either side of the *piggyBac* transposon (containing puromycin/TK selection cassettes) using the BsiW1 and Nsi1 cut-sites (supplemental material and methods). Co-transfection was performed as described above. After 48 h, cells were cultured in a 10 cm dish under puromycin (1 μg/mL) (Sigma-Aldrich, Missouri, USA)^65^^,^^66^ for 14 days, replacing media every 2–3 days, to select for cells containing the *piggyBac* transposon. Following selection, single puromycin-resistant cells were isolated and clonally expanded. Cells were screened via PCR and Sanger sequencing to confirm successful insertion of the *piggyBac* transposon and successful editing of rs16167640 from the parental A/A genotype to C/C genotype (primer sequences listed in Table S7). Isogenic HEK-293 cells containing the *piggyBac* transposon were subsequently transfected with 2.5 μg of *piggyBac* transposase in a six-well plate using lipofectamine LTX (Thermo Fisher Scientific, Massachusetts, USA) to remove the *piggyBac* selection cassette from the genome. 48 h after transfection, cells were subject to 200 nM Fialuridine (FIAU) (Sigma-Aldrich, Missouri, USA) selection for 7 days, replacing media every 2–3 days, to select for cells no longer carrying the *piggyBac* transposon cassette. Following selection, single FIAU-resistant cells were isolated and clonally expanded. Cells were screened via PCR (Table S7) and Sanger sequencing to confirm complete removal of the *piggyBac* transposon and successful editing of rs1617640. Successfully edited isogenic HEK-293 cells were subjected to qRT-PCR of the *EPO* gene to measure *EPO* gene expression levels and investigate the effect of the *cis*-*EPO* SNP on dysregulated Notch signaling genes identified through transcriptomic profiling of the *EPO*^*−/−*^ (*HEY2*, *DTX3L*, *PARP9*).

### Using the *cis-EPO* variant to examine the therapeutic profile and cardiovascular risk of genetically proxied therapeutic modulation of endogenous EPO levels

#### Drug-target two-sample Mendelian randomization

To investigate the association between higher endogenous EPO levels and risk of CVD, we performed a two-sample MR by using the *cis*-*EPO* variant as the genetic instrument. We obtained genotype-exposure association statistics from our EPO meta-analysis (n = 6,127). Primary outcomes were CAD, MI, or stroke using GWAS data consisting of 60,801, 42,561, and 40,585 cases, respectively^64^^,^^67^ (Table S8). We also performed a GWAS using UKB on CAD, MI, or stroke in 37,741, 105,90, and 9,092 cases, respectively (supplemental materials and methods, Table S8). Where we had genotype-outcome association data from both UKB and publicly available GWASs, we performed an inverse variance-weighted, fixed-effects meta-analysis by using metan^68^ to estimate the overall genotype-outcome association effect estimate. As only one genetic variant was used as an instrument, we calculated an overall causal estimate between the exposure and outcome by using the Wald ratio.^69^ We also performed MR to investigate the effect of higher circulating EPO levels on levels of clinical markers (systolic blood pressure [SBP], diastolic blood pressure [DBP], and resting heart rate) predisposing to CVD risk factors^70^^,^^71^ (supplemental materials and methods, Table S8).

#### Comparing clinical trial effects and genetic association to estimate the genetically predicted impact of therapeutically altered endogenous EPO levels on cardiovascular risk

To scale the genetic effect estimates to a more representative, therapeutically relevant effect, we obtained the effects of a PHI in patients on dialysis from a phase II fixed dose randomized control trial (RCT).^72^ The RCT provided an estimate of the effect of a fixed dose of HIF-PHIs on EPO levels during the first 4 weeks (median “maximum” change in EPO levels from baseline at week 4 [27.1]/SD at baseline [61] = 0.44 SD).^72^ The scaling factor was calculated by dividing the PHI-induced effect (0.44 SD) by the effect of the *cis-EPO* SNP on EPO levels (0.063 SD). We used this value (7.05) to scale the genetically instrumented effect estimates and 95% confidence intervals of the *cis-EPO* SNP on CVD or clinical markers for CVD risk factors to the effect of PHIs on endogenous EPO levels.

### Phenome-wide association study (PheWAS)

To further investigate the therapeutic profile of modulated EPO levels, we tested the association of rs1617640 near *EPO* with 869 traits in up to 451,099 UKB individuals of European ancestry (supplemental materials and methods). Genotype-phenotype associations were generated using BOLT-LMM^73^ and traits were selected as previously described in Frayling et al.^74^ For continuous traits, we performed inverse normalization prior to regression analysis to account for skewed distributions.

## Results

### Identification of three genomic loci associated with EPO levels at genome-wide significance

To identify human genetic variants associated with circulating EPO levels and using these variants as genetic predictors of therapeutically elevated endogenous EPO levels, we performed a GWAS meta-analysis of circulating EPO. We used 6,127 individuals of European and African American descent. After conditional analysis, we identified three genomic loci containing three independent signals associated with circulating EPO (p < 5 × 10^−8^) (Table S1, Figure S6). The most strongly associated genetic variant, rs4895441 (6q23, *HBS1L-MYB* locus), had been previously identified as associated with circulating EPO levels in a GWAS of 2,691 individuals.^75^ This variant has stronger primary effects on other erythrocyte phenotypes in previously published GWASs and a PheWAS in UK Biobank (UKB) European individuals (Table S9). The remaining two independent genomic loci (rs855791 and rs112631630) represent novel associations with circulating EPO levels. However, rs855791, located in the *TMPRSS6* locus, has primary, stronger effects on several other erythrocyte phenotypes compared to the effect on circulating EPO in a PheWAS in UKB European individuals (Table S9) and has been previously associated with other erythrocyte phenotypes and iron homeostasis biomarkers in GWASs.^76^^,^^77^ The variant (rs112631630) located in the *NRAP* locus is only present in one study (African Americans), and we were unable to test in additional datasets. Therefore, these variants were not deemed suitable, specific instruments used in subsequent MR analysis to genetically predict therapeutic modulation of endogenous EPO levels.

### Identification of *cis*-SNP lying in the promoter region of *EPO* for use as a genetic predictor for the therapeutic alteration of endogenous EPO levels

The conditionally independent lead variants identified by the GWAS meta-analysis were not sufficiently specific instruments to act as genetic predictors for higher endogenous EPO levels. We therefore looked for *cis* effects at the *EPO* locus. Previous associations have been reported between variants near *EPO* and circulating EPO levels.^78^^,^^79^ A *cis-*SNP, rs1617640, lying in the *EPO* promoter region, 1,125 bp upstream of the transcription start site, was associated with EPO levels in our study and in the previous study.^78^ On the basis of our meta-analysis, each copy of the A-allele at rs1617640 was associated with 0.063 standard deviations (SD), equivalent to 0.32 IU/L, higher endogenous EPO levels *(*p *=* 9.32 × 10^−4^) (Table 1). The effect of the *cis-EPO* variant is consistent with that previously reported in affected individuals with diabetic retinopathy or hepatitis C.^78^^,^^79^

**Table 1:** Summary statistics for association between the *cis-EPO* genetic variant (rs1617640) and circulating EPO levels or hepatic *EPO* gene expression

**Analysis**	**RSID**	**Chromosome**	**Position**	**A1**	**A2**	**Freq A1**	**β**	**SE**	**p value**	**Sample size**
EPO meta-analysis	rs1617640	7	100,317,298	A	C	0.62	0.063	0.02	9.32E−4	6,127

**Analysis**	**RSID**	**Chromosome**	**Position**	**A1**	**A2**	**Freq A1**	**t-meta**	**SE**	**p value**	**Sample size**
Liver eQTL analysis	rs1617640	7	100,317,298	A	C	0.62	5.39	0.15	6.86E−8	861

### The *cis-EPO* SNP is associated with altered *EPO* expression and nearby *TFR2* expression in the liver

**Figure 1:**
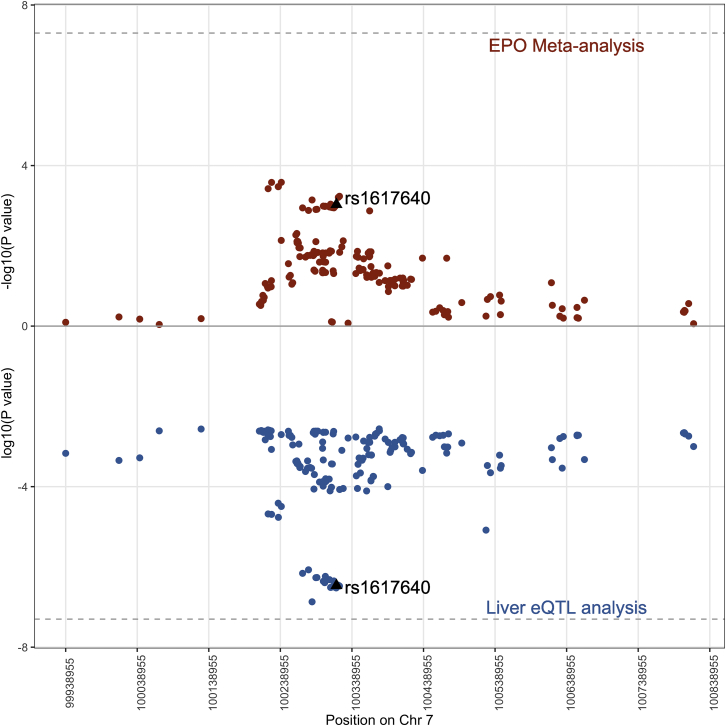
Colocalization analysis of circulating endogenous EPO levels and liver *EPO* gene expression shows that the same genetic variant (*cis-EPO* variant) is likely to be the causal variant (posterior probability = 71%) We tested for colocalization within a 500 kb flanking window of the *cis-EPO* genetic variant (rs1617640—labeled and represented as a black diamond on the plot). The top half of the Miami plot (red dots) represents −log_10_(p) for the circulating EPO levels obtained from the EPO meta-analysis whilst the bottom half of the Miami plot (blue dots) represents log_10_(p) for *EPO* gene expression in the liver obtained from a hepatic eQTL meta-analysis. The dashed lines represent genome-wide significance levels of p = 5 × 10^−8^.

To provide additional insight into the rs1617640-EPO association and further evaluate its utility as an instrument, we tested the association of the *cis-EPO* SNP with gene expression in the kidney and the liver as *EPO* is highly expressed in these two tissues.^80^ In the liver, we found that the C allele at rs1617640 was associated with higher *EPO* expression (β = 0.22 [0.14, 0.3], p = 6.86 × 10^−8^) and also *TFR2* expression (β = 0.23 [0.17, 0.29], p = 1.56 × 10^−13^), a gene that lies upstream of the *EPO* gene and is involved in iron metabolism (Table S3).^76^^,^^81^ No effect of rs1617640 on renal expression of *EPO* (β = 0.16 [−2.46, 2.78], p > 0.05) or *TFR2* (β = 1.33 [−0.59, 3.26], p > 0.05) was found (Table S4). We, therefore, proceeded with hepatic results only. Colocalization analysis provided evidence that the variant associated with circulating EPO levels in the meta-analysis and hepatic *EPO* mRNA expression has a 71% posterior probability of being the casual variant (Figure 1).

### Functional validation of rs1617640 in influencing *EPO* expression

**Figure 2:**
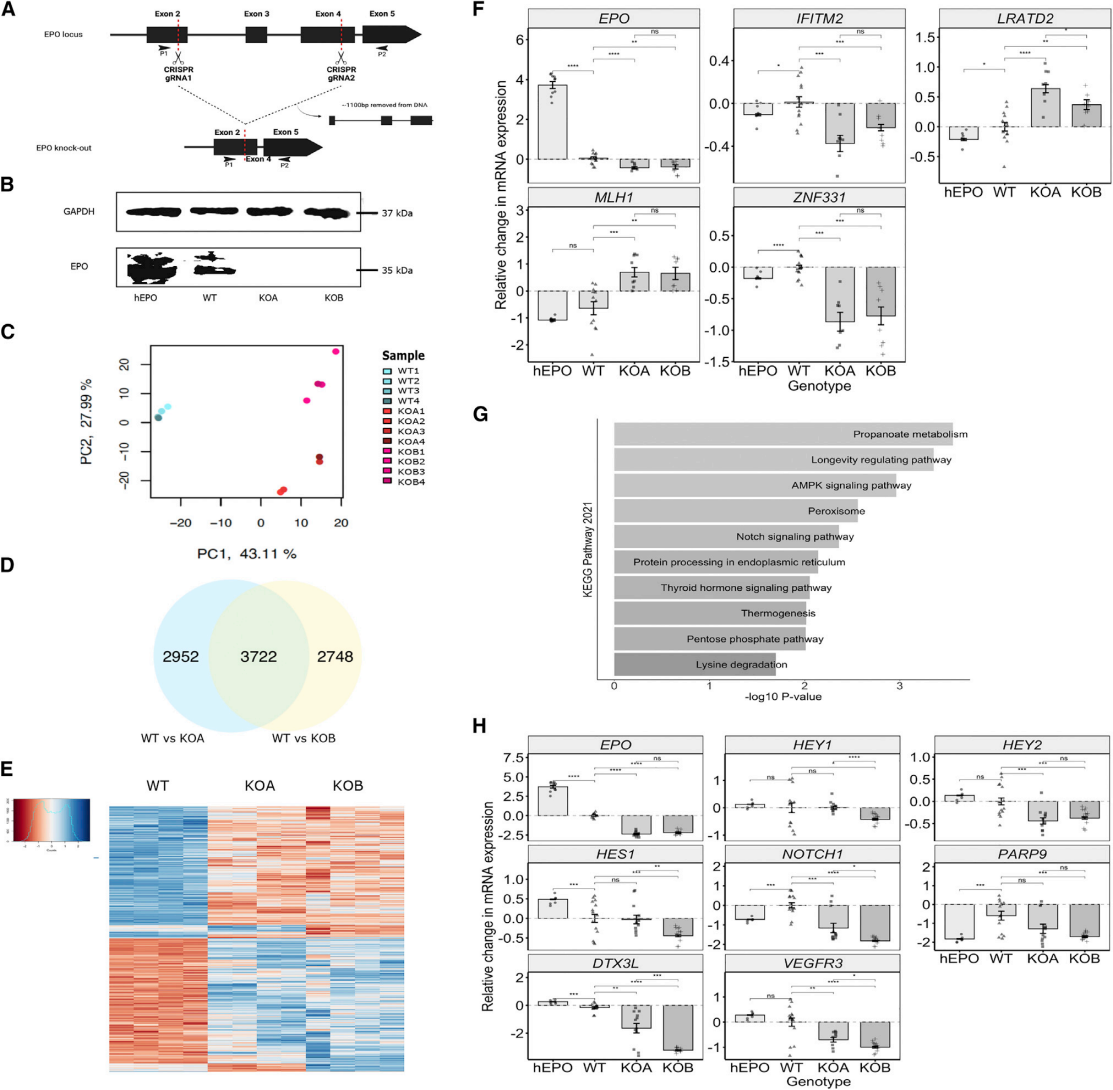
Establishment of whole *EPO* gene knockout in HEK-293 cells using CRISPR-Cas9 gene-editing technology with paired gRNAs and whole transcriptomic profiling of knockouts to identify differentially expressed genes (A) Hypothetical schematic of the construction of the *EPO*^*−/−*^*.* Successful targeting of the paired gRNAs would result in the introduction of two double-stranded breaks (DSBs) and the removal of the intervening region from the genomic DNA (∼645 bp) after DNA repair via non-homologous end joining (NHEJ). The location of the paired gRNAs designed to target the *EPO* gene (ENST00000252723.3) are labeled and highlighted by the red dotted lines. P1 and P2 represent location of primers used for genotyping potential knockouts. (B) Immunoblotting analysis. Immunoblot analysis, probing for EPO and GAPDH as a control, revealed reduced EPO protein expression levels in both *EPO*^*−/−*^ cell lines (KOA and KOB) compared to WT controls and cells transfected with an *EPO* over-expression construct (hEPO). (C) PCA plot illustrating the transcriptomic profiles of the *EPO*^*−/−*^ cell lines compared to WT HEK-293 controls. The plot shows the results obtained from RNA-seq analysis of four WT samples, four KOA samples, and four KOB samples. (D) The number of differentially expressed genes (DEGs) (p ≤ 0.05) obtained by performing differential gene expression analysis comparing WT to KOA and WT to KOB. (E) Heatmap illustrating the expression profile of the 3,722 overlapping DEGs. The red blocks represent down-regulated genes, and the blue bocks represent up-regulated genes; the color scale of the heatmap represents the DEG expression level. (F) qRT-PCR of four of the top DEGs identified by differential gene expression analysis to validate our RNA-seq findings. The graph shows the relative change in mRNA expression levels (+/− SEM) between genotypes. (G) The top ten KEGG Pathways identified through GEO analysis of the 3,501 overlapping with consistent direction of effects. Analysis was performed with Enrichr online tool. The color scale represents the log_10_ p value. (H) qRT-PCR of genes involved in the Notch signaling pathway identified by differential gene expression analysis to determine whether *EPO*^*−/−*^ results in altered Notch signaling activity. hEPO represents WT HEK-293 cells transfected with an over-expression *EPO* construct. The graph shows the relative change in mRNA expression levels (+/− SEM) between genotypes. Columns and error bars in (F) and (H) represent mean and SEM values. Paired t test was performed. ns, non-significant; ^∗^p ≤ 0.05, ^∗∗^p ≤ 0.01, ^∗∗∗^p ≤ 0.001, ^∗∗∗∗^p ≤ 0.0001.

To further validate the rs1617640 variant, we first sought a better understanding of the downstream causal genes and signaling cascades of EPO. We generated two *EPO* knockout (*EPO*^*−/−*^*)* cell lines by using CRISPR-Cas9 gene editing with a paired gRNA approach (Figures 2A and 2B, Figure S7). By performing RNA sequencing (RNA-seq) analysis, we found large transcriptional differences between *EPO*^*−/−*^ and wild-type (WT) cell lines (Figures 2C and S8). To obtain a list of differentially expressed genes (DEGs) specific to *EPO* knockdown and not due to potential differences in cellular conditions, we performed differential gene expression analysis by comparing controls to the two *EPO*^−/−^ cell lines, KOA and KOB, (Figures S9A and S9B) and then combined lists of DEGs to obtain a final list of 3,722 overlapping DEGs (p ≤ 0.05) (Figures 2D and 2E, Table S10). 3,501 of the overlapping DEGs showed consistent directions of differential expression (Figure S9C, r^2^ = 0.90, p < 2.2 × 10^−16^) and were considered for downstream analysis. 314 of the 3,501 had a log_2_ fold-change ≥ |2|. We validated the differential expression of four DEGs (p ≤ 0.05, log_2_ fold-change ≥ |2|) with the highest expression in HEK-293 cells (based on the Human Cell Atlas^82^) by using qRT-PCR (Figure 2F). Gene Ontology (GO) analysis of the 3,501 DEGs suggested enrichment for multiple biological processes involved in DNA repair, mRNA processing, ATPase activity, notch signaling, apoptosis, fatty acid oxidation, and cellular respiration (Table S11). Notch signaling and related mitogenic pathways featured prominently in these GO analyses (Figure 2G), so we selected seven genes from these pathways and validated differential expression with qRT-PCR (Figure 2H).

**Figure 3:**
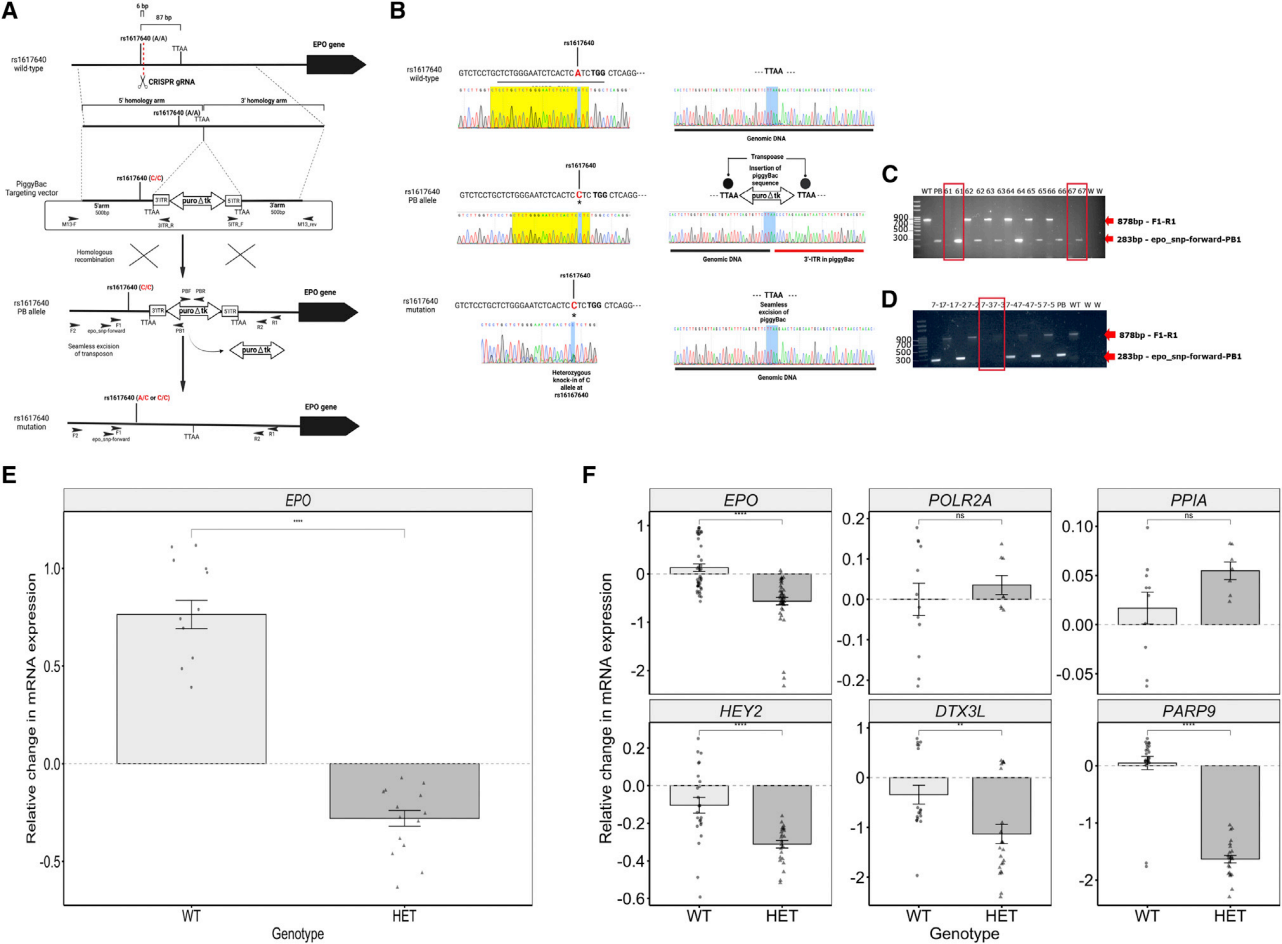
Establishment of heterozygous knockin at rs1617640 (A/C) in HEK-293 cells using CRISPR-Cas9 gene-editing technology alongside *piggyBac* transposon system (A) Schematic of the generation of a SNP knockin model of rs1617640 using CRISPR-Cas9 gene editing alongside the *piggyBac* transposon system. The location of rs1617640 (A/A) and the site at which the gRNA is designed to introduce a DSB within the wild-type sequence of HEK-293 cells is shown in the top panel. Two homology arms either side of the closest TTAA site are designed with one (5′ arm) containing the desired gene edit at rs1617640 (C/C). The two homology arms are cloned into the targeting construct either side of the *piggyBac* transposon carrying the selectable markers, *puroΔtk.* Upon introduction of a DSB at the site targeted by the gRNA, DNA repair via homologous recombination due to the presence of the homologous arms is initiated and the the *piggyBac* transposon becomes integrated into the genomic DNA at the TTAA site. After selection with puromycin, clones with mutation-corrected lines were identified and transiently transfected with *piggyBac* transposase plasmid, followed by FIAU treatment to eliminate *piggyBac*-containing clones. Mutation-corrected heterozygous clones for rs1617640 (A/C genotype) were isolated with no marks remaining within the genomic DNA. (B) Sanger sequencing results of the sequence at each stage of the CRISPR-Cas9 and *piggyBac* transposon technique. The top panel represents the wild-type sequence at rs1617640 and the wild-type sequences up- and downstream of the TTAA site prior to gene editing. The middle panel represents the mutated sequence at rs1617640 and the insertion of the *piggyBac* transposon sequence at the TTAA site following homologous recombination after successful introduction of the DSB. The bottom panel represents the seamless excision of the *piggyBac* transposon from the genomic DNA. The yellow highlighted region represents the gRNA sequence. (C) PCR gel electrophoresis confirming the insertion of the *piggyBac* transposon in clones 61 and 67. (D) PCR gel electrophoresis confirming successful removal of the *piggyBac* transposon from the genome following treatment with the transposase in clone 7-3. (E) qRT-PCR of *EPO* to validate rs1617640 as causal in controlling *EPO* mRNA expression levels. The graph shows the relative change in mRNA expression levels (+/− SEM) between genotypes (A/A and A/C). (F) qRT-PCR of genes involved in the Notch signaling pathway (*HEY2*, *DTX3L*, and *PARP9)* and two control genes (*PPIA* and *POLR2A*). *EPO* was repeated again as a positive control for altered expression for comparison. The graph shows the relative change in mRNA expression levels (+/− SEM) between genotypes. Columns and error bars in (E) and (F) represent mean and SEM values. Paired t test was performed. ns, non-significant; ^∗^p ≤ 0.05, ^∗∗^p ≤ 0.01, ^∗∗∗^p ≤ 0.001, ^∗∗∗∗^p ≤ 0.0001.

To functionally validate the *cis*-*EPO* SNP as the most likely causal variant in controlling *EPO* expression levels and therefore the most valid instrument for use to genetically predict the associated risk of CVD, we used CRISPR-Cas9 and the *piggyBac* transposon system to generate two isogenic cell lines, one homozygous for the A allele (A/A) and one heterozygous for the A allele (A/C) at rs1617640 (Figures 3A–3D, S10, and S11). We performed qRT-PCR in the A/A and A/C isogenic cell lines. We showed that homozygous cells for the A allele of rs1617640 had higher *EPO* mRNA expression levels than heterozygotes for the A allele, confirming that rs1617640 has an allele-specific effect on *EPO* gene expression levels (Figure 3E). These results are consistent with our genetic findings that the A allele is associated with higher circulating EPO levels. We also performed qRT-PCR on three Notch-signaling genes (*HEY2*, *DTX3L*, *PARP9*), which showed differential expression in the *EPO*^*−/−*^ knockouts compared to wild-type, to see whether specific alteration of the *cis-EPO* variant also resulted in dysregulated Notch signaling. We found that A allele homozygotes of rs1617640 had a down-regulated expression of these Notch-signaling genes compared to heterozygotes (Figure 3F). Negative control experiments with two genes not differentially expressed in *EPO*^−/−^ confirmed that the *cis*-SNP editing was specific to the EPO pathways (Figure 3F). As the eQTL analysis showed that the *cis-EPO* SNP was associated with hepatic *TFR2* expression, we also performed qRT-PCR to further investigate the potential pleiotropic effect of the *cis-*SNP. We found no difference in *TFR2* mRNA expression in A allele heterozygotes compared to A allele homozygotes or *EPO*^*−/−*^ knockouts (Figure S12).

### Genetically predicted long-term higher endogenous EPO levels are not associated with increased cardiovascular risk

We used the *cis-EPO* SNP as an instrument in two-sample Mendelian randomization (MR) as a genetic predictor for therapeutically altered EPO levels on risk of CVD or levels of clinical markers predisposing to CVD risk factors. The three main CVDs of interest were coronary artery disease (CAD; 98,542 cases, 442,396 controls), myocardial infarction (MI; 53,151 cases, 564,013 controls), and stroke (49,677 cases, 752,534 controls) because of availability of large-scale genetic association data. The clinical markers of interest were systolic blood pressure (SBP; n = 678,320), diastolic blood pressure (DBP; n = 677,567), and resting heart rate (n = 514,695). Using these very large sample sizes, we found no evidence (at p < 0.05) of a genetic association between 1 SD higher endogenous EPO levels (equivalent to 5.1 IU/L) and increased odds of CAD (odds ratio [OR] [95% CI] = 1.03 [0.85, 1.25], p = 0.72), stroke (OR [95% CI] = 0.92 [0.70, 1.21], p = 0.55), or MI (OR [95% CI] = 0.98 [0.75, 1.29], p = 0.89) (Table S12). We found evidence of a genetic association between 1 SD, equivalent to 5.1 IU/L, higher endogenous EPO levels, and lower resting heart rate (effect estimate [95% CI] = −0.996 [−1.74, −0.25], p = 0.01) and lower DBP (effect estimate [95% CI] = −0.98 [−1.67, −0.29], p = 0.006) but not with SBP (effect estimate [95% CI] = 0.53 [−0.65, 1.71], p = 0.38) (Table S12).

### Comparison of the genetic associations with the effects observed in clinical trials

**Figure 4:**
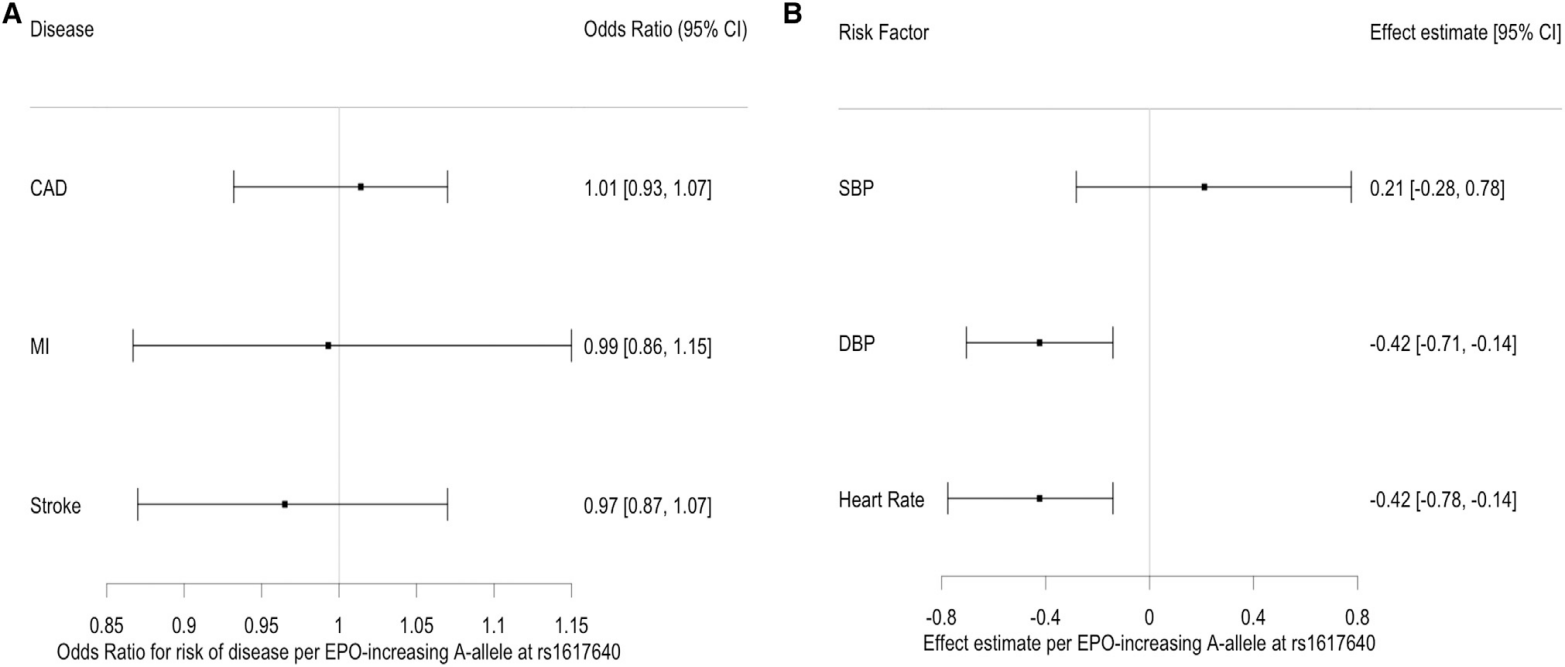
Genetically predicted therapeutic rises in endogenous EPO levels are not associated with an increased risk of CVD or clinical markers for CVD risk factors We rescaled the genetic estimates obtained through two-sample MR by using the *cis-EPO* SNP to genetically estimate the effect of therapeutic rises in endogenous EPO levels to the PHI-induced effect reported in a phase II RCT. The black point on the plot represents the odds ratio and the bars represent the 95% confidence intervals.For all other figures, elements have been defined in the corresponding figure legends. (A) On the basis of the upper confidence interval, we were able to exclude increased odds of 1.07, 1.15, and 1.07 for CAD, MI, or stroke, respectively with 2.2 IU/L genetically mediated higher endogenous EPO levels. (B) On the basis of the upper confidence interval, we were also able to exclude levels higher than 0.21 mmHg for SBP and no increase in DBP or resting heart rate with genetically mediated therapeutic higher endogenous EPO levels.

In an RCT, individuals receiving a PHI (daprodustat) had EPO levels 0.44 SD (27.1/61), equivalent to 2.2 IU/L, higher than baseline EPO levels.^72^ Given that the per-allele effect of rs1617640 on endogenous EPO was 0.063 SD, we rescaled our genetic association by multiplying by 7.05 (0.44/0.063) to obtain a clinically relevant estimate of the likely impact of genetically predicted therapeutic rises in endogenous EPO on cardiovascular risk. Using this scaling factor allowed us to quantify the upper and lower bounds on the genetically predicted effects of long-term endogenous EPO rises on CVD equivalent to therapeutically, physiologically relevant effects (Figure 4, Table S13). By rescaling the genetic associations to the PHI-induced effects of genetically predicted therapeutic rises in endogenous EPO levels, the odds of disease were 1.01 (95% CI = 0.93, 1.07) for CAD, 0.99 (95% CI = 0.87, 1.15) for MI, and 0.97 (95% CI = 0.87, 1.07) for stroke (Figure 4, Table S13). On the basis of the upper 95% confidence intervals, we could exclude a 1.07, 1.15, and 1.07 increased odds of CAD, MI, or stroke, respectively (Figure 4, Table S13). For the clinical markers predisposing to CVD, we did not observe an association between higher genetically predicted therapeutic rises in endogenous EPO levels and SBP (β [95% CI] = 0.21 [−0.28, 0.78]), DBP (β [95% CI] = −0.42 [−0.71, −0.14]), or resting heart rate (β [95% CI] = −0.42 [−0.78, −0.14]). On the basis of the upper confidence intervals, we could exclude 0.78 mmHg increased SBP levels and any increase in DBP or resting heart rate (Figure 4, Table S13).

### The *cis-EPO* SNP is associated with several relevant erythrocyte phenotypes and no unintended effects or diseases

To further determine the specificity of the *cis-EPO* SNP as a genetic instrument for endogenous, physiological EPO levels and identify any potential additional, unintended effects that may be associated with long-term rises in genetically predicted endogenous EPO levels, we tested the association between the *cis*-*EPO* SNP and 869 traits in up to 451,099 unrelated European UKB individuals. We found the *cis*-*EPO* SNP was associated with 18 relevant erythrocyte traits (p < 5 × 10^−8^) with effects between 0.01 and 0.06 SD (Figure S13, Table S14). We also found evidence for an association between the *EPO*-increasing A allele of rs1617640 and decreased fibrosis-4 score (β = −0.01, p = 4.7 × 10^−17^) and non-alcoholic fatty acid liver disease (NAFLD) fibrosis score (β = −0.02, p = 2.20 × 10^−25^) (Figure S13, Table S14). However, these associations were not clinically significant (equivalent to a 0.06 and 0.07 change in fibrosis-4 or NAFLD for 1 IU/L increase in EPO levels). These associations are most likely driven by the strong association with higher platelet counts (β = 0.02, p = 4.7 × 10^−39^). We did not find evidence for an association between genetically predicted higher endogenous EPO levels and other unintended effects or diseases.

## Discussion

We have shown how a combination of genetic analyses and functional validation studies provides a powerful approach to assess the therapeutic profile and effects of long-term genetically mediated alterations in drug target levels. Several lines of evidence indicate the *cis-EPO* variant is an excellent proxy to naturally mimic the pharmaceutical effects of raising endogenous EPO levels and test effects on cardiovascular risk. First*,* we found the A allele of rs1617640 increases EPO levels in a meta-analysis of circulating EPO levels (Table 1). Second, we found the *cis-EPO* SNP is associated with hepatic *EPO* gene expression and, from colocalization analysis, is likely to represent the same causal variant (Table 1, Figure 1). Third, by establishing a heterozygous knockin cell model using CRISPR-Cas9 single-base gene editing, we provided evidence that the *cis-EPO* variant has an allele-specific effect on *EPO* expression levels with homozygotes of the A allele having higher *EPO* expression compared to heterozygotes for the A allele (Figure 3). Our findings help clarify previous studies that found rs1617640 to have an allele-specific effect on *EPO* expression levels. Some had reported the A allele to be associated with higher EPO concentrations,^78^^,^^79^ while others reported conflicting evidence with the C allele being associated with higher EPO concentrations and promoter activity.^83^^,^^84^ We found an association between the variant and hepatic *EPO* gene expression; however, the C allele was found to be associated with increased hepatic *EPO* expression while the A allele was found to increase endogenous EPO levels (Table 1). We did not find an association between the variant and renal gene expression despite the CRISPR-Cas9 gene functional work in an embryonic kidney cell line. These differences could be due to lack of power attributable to the small sample size (n = 236 kidneys) compared to the liver dataset. The rs1617640 SNP has different effects depending upon cell type, physiological condition, state, and timing alongside the complex compensatory feedback mechanisms involved in EPO signaling.^85^ When testing the *in vitro* effects of *EPO* gene knockdown, we indicate a role for EPO in Notch signaling. The Notch-signaling pathway is known to play a role in cell-cycle signaling, cell-fate specification, and metabolic processes and therefore may represent the broad mitogenic effects of EPO.^86^^,^^87^ We also found the *cis-EPO* SNP to alter gene expression levels of Notch-signaling genes that were identified through whole-transcriptomic profiling of *EPO*^*−/−*^ cell lines, indicating that rs1617640 is important in controlling *EPO* expression levels and in downstream implicated pathways involved in cell-cycle activity, DNA repair, and metabolic processes (Figures 2 and 3).

Having provided genetic and functional evidence that a *cis*-*EPO* variant alters *EPO* gene expression and circulating protein levels, we used this variant as a genetic predictor for long-term therapeutic modulation of EPO levels to show that genetically predicted higher endogenous EPO levels (equivalent to 5.1 IU/L) are not associated with increased cardiovascular risk or elevated values of clinical markers (SBP, DBP, or resting heart rate) predisposing to CVD risk factors (e.g., hypertension). To obtain a more representative effect of therapeutically altered endogenous EPO levels, we rescaled these genetic effects to the PHI-induced effects on endogenous EPO levels from a fixed dose phase II trial^72^ and did not observe an association between genetically predicted therapeutic rises in endogenous EPO levels and risk of CVD (Figure 4). Using the upper bound of the confidence interval, we were able to statistically exclude increased odds of 1.07, 1.15, and 1.07 for CAD, MI, or stroke, respectively, and 0.78 mmHg increased levels of SBP (Figure 4). We were able to exclude adverse effects on DBP and heart rate (Figure 4). Our results are consistent with the hypothesis that PHIs are not likely to increase CVD risk or CVD risk factors in treating anemia of CKD when increasing circulating EPO levels within the physiological range. Scaling genetic estimates to the effect of a fixed dose after 4 weeks of treatment may not be the most clinically relevant because PHIs require titration to a hemoglobin target, but this was the best estimate available, as changes in EPO levels were only measured in the one fixed dose phase II trial.^72^ Because all PHIs work through the same mechanism, these genetically predicted effects of therapeutically altering endogenous EPO levels are likely applicable to all PHI compounds. Any slight differences in effects on EPO levels and CVD risk between HIF-PHI compounds would most likely be related to independent biochemical properties of the compounds, variations in dosages, and the effects of PHIs on transcription of other hypoxic response genes, which were not investigated in this study.

There are some limitations to our study. As with any study using human genetics as predictors, our results cannot rule out effects but instead can provide upper bounds on their probability.^88^ First, here we performed the genetic analyses in a general, “healthy” population as opposed to a diseased cohort in whom the treatment is used. Diseased populations may respond differently to that estimated by the genetic association as a result of having variable baseline EPO levels or additional underlying conditions.^89^^,^^90^ Despite rescaling the genetic effect to the PHI-induced effect to try and overcome this, we still assume linearity, which may not be the case for the therapy. For example, MR estimates could change depending on baseline levels, and therefore inferences about the likely effect at the individual level need careful consideration, particularly when doses are titrated.^90^ As larger studies become available, particularly in disease cohorts, our ability to detect associations and perform stratified analyses will increase. We will become more confident about the conclusions drawn from these types of investigations.^90^^,^^91^ Second, common genetic variants differ from clinical trials in that they represent subtle, life-long perturbations, whereas clinical trials test more acute larger changes.^92^^,^^93^ In addition, therapeutic interventions may result in localized effects at a particular time, titration to a particular level resulting in different individual-level biomarker increases, or may only be efficacious in a certain physiological state, which is difficult to accurately predict using genetics.^89^^,^^93^ Third, as with any MR investigation, pleiotropy could lead to biased estimates. As we only used a single genetic instrument, we were unable to fully test for horizontal pleiotropy using established statistical methods and it remains possible that our MR findings are confounded by the variant’s association with *TFR2*, as well as *EPO.* The presence of the variant in the *EPO* promoter, and the alteration of *EPO* expression, but not *TFR2* expression, in our single-base editing experiments suggests *EPO* is the main target. The single-base editing enabled separation of the primary effect of the *cis-*SNP on *EPO* from a likely secondary effect on *TFR2* (Figure S12) eliminating some concerns of biased estimates due to horizontal pleiotropy.

In summary, this study indicates that genetically predicted long-term rises in endogenous EPO levels do not increase cardiovascular risk, with upper limits of 1.07, 1.15, and 1.07 for CAD, MI, and stroke, respectively, given a clinically relevant 2.2 unit rise in endogenous EPO levels. These estimates were established using extremely large case-control studies. Our functional evidence using CRISPR-Cas9 and the *piggyBac* system to change the allele at rs1617640 validated the *cis-EPO* SNP as a partial proxy for therapeutically altered endogenous EPO. We have shown how genetic analyses combined with functional validation studies represent a powerful approach to identify relevant genetic markers that can investigate the long-term effect of therapeutic action.

### Data and code availability

The code generated during this study are available at https://github.com/CharliHarlow/EPO_metaanalysis_rnaseq_MR_phewas. The accession number for the summary-level EPO meta-analysis data generated in this paper is Zenodo: 6811853 (https://zenodo.org/record/6811853). The accession number for the raw sequencing read data generated in this paperr is Zenodo: 6811704 (https://zenodo.org/record/6811704#.Ys1Jzy2ZPOQ). The published article includes the rest of the data generated or analyzed during this study.

## Web resources

AddGene, https://www.addgene.org

Benchling, https://www.benchling.com

BioRender, https://biorender.com

BLAST, https://www.ensembl.org/Multi/Tools/Blast

Ensembl, https://www.ensembl.org/index.html

Enrichr, https://maayanlab.cloud/Enrichr/

GEMMA, https://www.xzlab.org/software/GEMMAmanual.pdf

Genewiz, https://www.genewiz.com/en-GB/

GTEx, https://gtexportal.org/

Human Cell Atlas, https://www.humancellatlas.org

METAL, https://genome.sph.umich.edu/wiki/METAL_Quick_Start#Meta-Analysis_Results

RefFinder, https://www.heartcure.com.au/reffinder/?type=reference

RStudio, https://www.rstudio.com

Stata, https://www.stata.com

## Supplemental information

Document S1. Figures S1–S13, Tables S1–S9, S12, and S13, and supplemental material and methods

Table S10. Differentially expressed genes with consistent direction of effects in both knockouts and adjusted p value < 0.05

Table S11. Gene Ontology analysis of the 3,501 DEGs identified through differential expression analysis of wild-type cells versus EPO knockout

Document S2. Article plus supplemental information
