# Supplementary material for: Identification and single-base gene-editing functional validation of a cis-EPO variant as a genetic predictor for EPO-increasing therapies
Source: Am J Hum Genet. 2022 Sep 1;109(9):1638–52. doi: 10.1016/j.ajhg.2022.08.004 (PMC9502050; doi:10.1016/j.ajhg.2022.08.004)
Supplement: Document S1. Figures S1–S13, Tables S1–S9, S12, and S13, and supplemental material and methods [file mmc1.pdf]

The American Journal of Human Genetics, Volume *109*

# Supplemental information

# Identification and single-base gene-editing functional validation of a *cis-EPO* variant as a genetic predictor for EPO-increasing therapies

**Charli E. Harlow, Josan Gandawijaya, Rosemary A. Bamford, Emily-Rose Martin, Andrew R. Wood, Peter J. van der Most, Toshiko Tanaka, Hampton L. Leonard, Amy S. Etheridge, Federico Innocenti, Robin N. Beaumont, Jessica Tyrrell, Mike A. Nalls, Eleanor M. Simonsick, Pranav S. Garimella, Eric J. Shiroma, Niek Verweij, Peter van der Meer, Ron T. Gansevoort, Harold Snieder, Paul J. Gallins, Dereje D. Jima, Fred Wright, Yi-hui Zhou, Luigi Ferrucci, Stefania Bandinelli, Dena G. Hernandez, Pim van der Harst, Vickas V. Patel, Dawn M. Waterworth, Audrey Y. Chu, Asami Oguro-Ando, and Timothy M. Frayling**

## Supplemental Figures

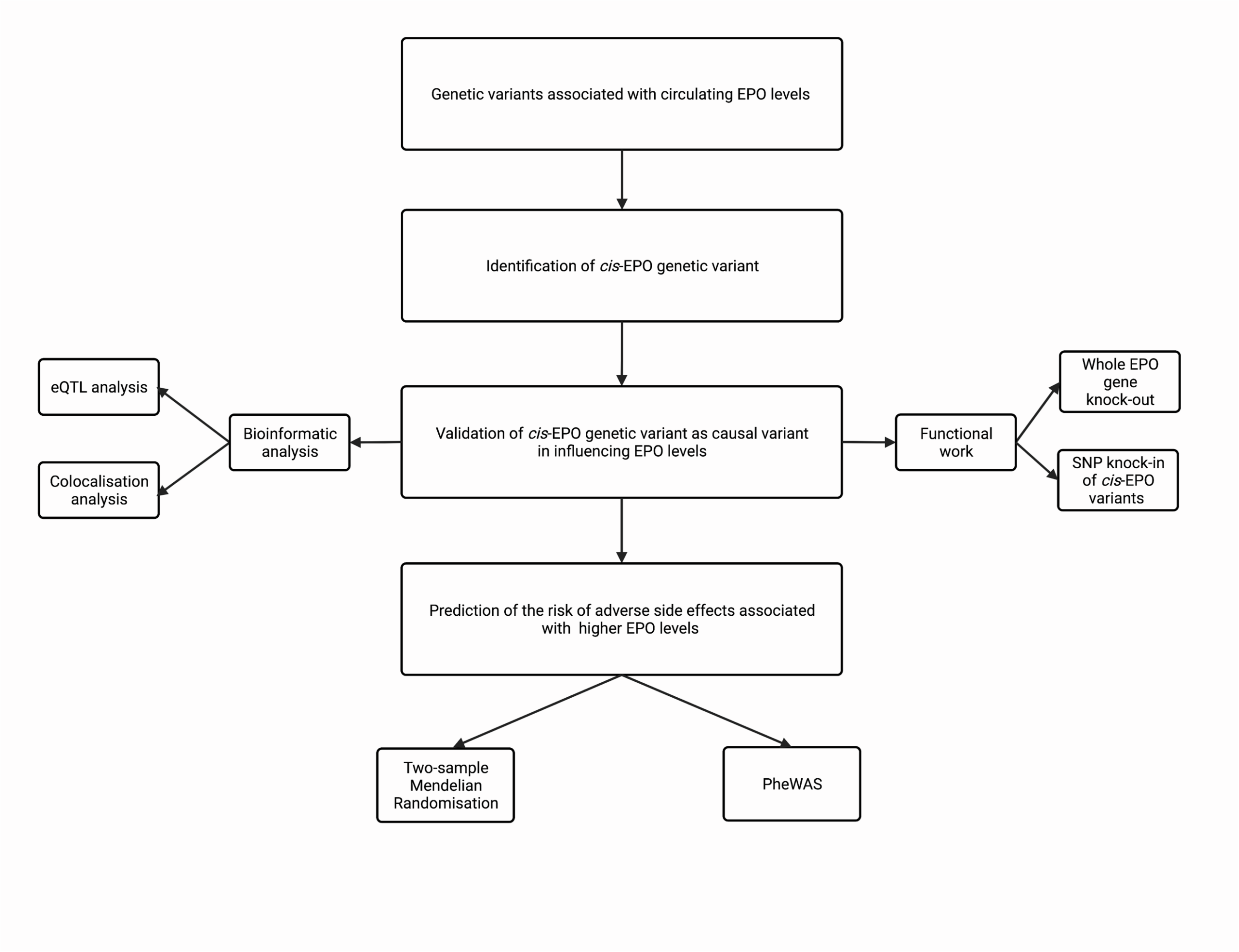


**Figure S1. Study design.** Using a combination of genetic studies and functional approaches, this study identified and validated a genetic variant lying in *cis* with the *EPO* gene for use as a proxy to characterize the therapeutic profile of long-term therapeutic rises in endogenous EPO levels particularly in terms of CVD risk.

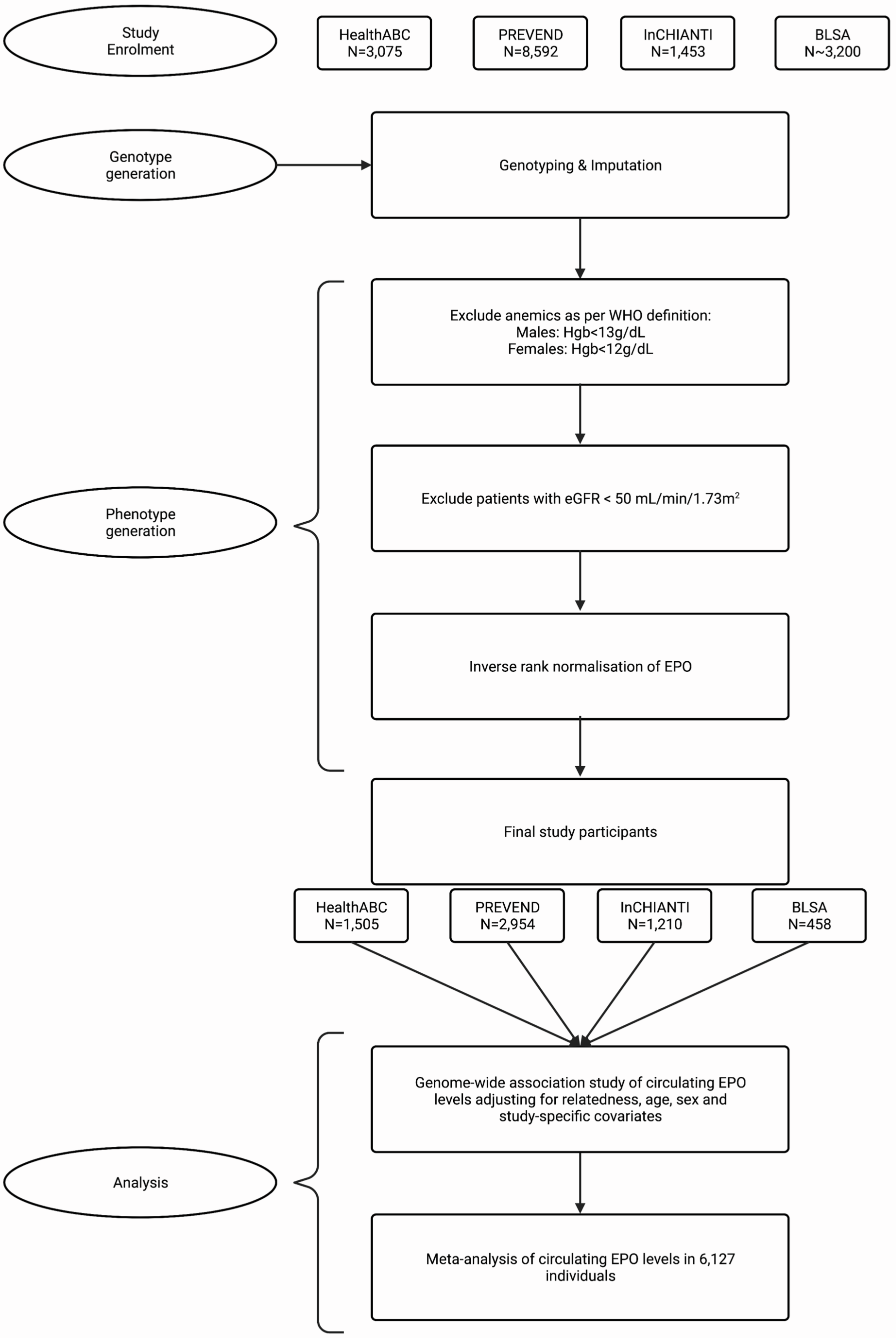


**Figure S2. Analysis plan for the genome-wide association study meta-analysis of circulating EPO levels.** Four independent cohorts with valid genotypic and phenotypic data were used for the meta-analysis of circulating EPO levels. The EPO phenotype was derived by excluding anemic individuals and those with an estimated glomerular filtration rate < 50 mL/min/1.73m$^2$. Phenotypes were then inverse rank normalized to account for the skewed distribution. GWAS was performed adjusting for age, sex, PCs and study specific covariates before a fixed-effects inverse variance-weighted meta-analysis was implemented. The final study sample size included 6,127 individuals of European and African American ancestry.

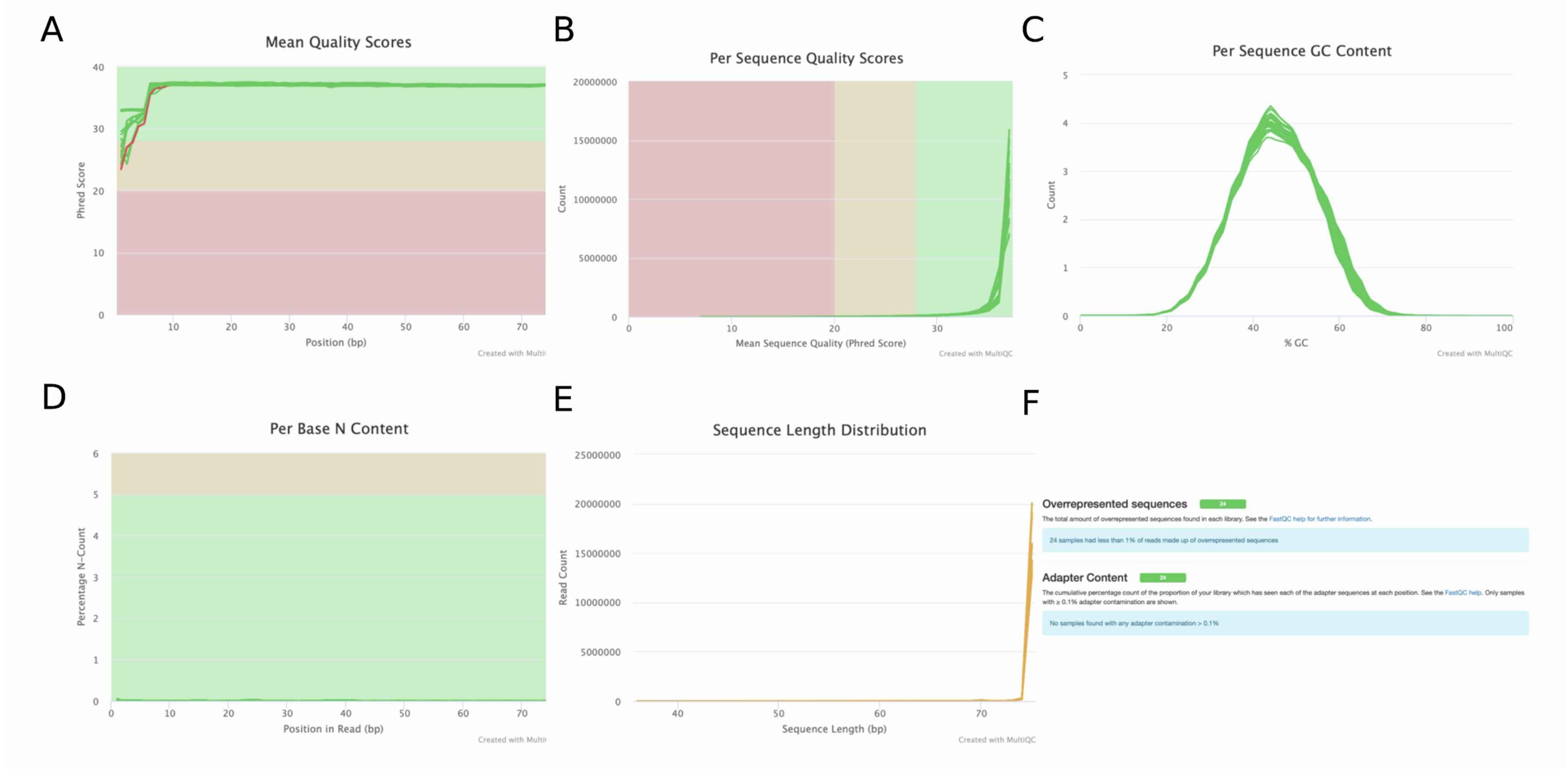


**Figure S3. Quality Control checks of the RNA-seq raw reads after trimming had been performed. a)** Plot of the Phred Scores for the 12 trimmed sequencing reads. All Phred scores are above 20. **b)** Per sequence quality plot. All sequencing reads had an average quality > 30. **c)** Plot showing the GC content per sequence. **d)** The number of bases read as ‘N’ along each sequencing read. **e)** The distribution of the sequence lengths across reads. **f)** Quality control check to see if all adapter sequences had been removed and if any sequences were over-represented. Images were produced using MultiQC.

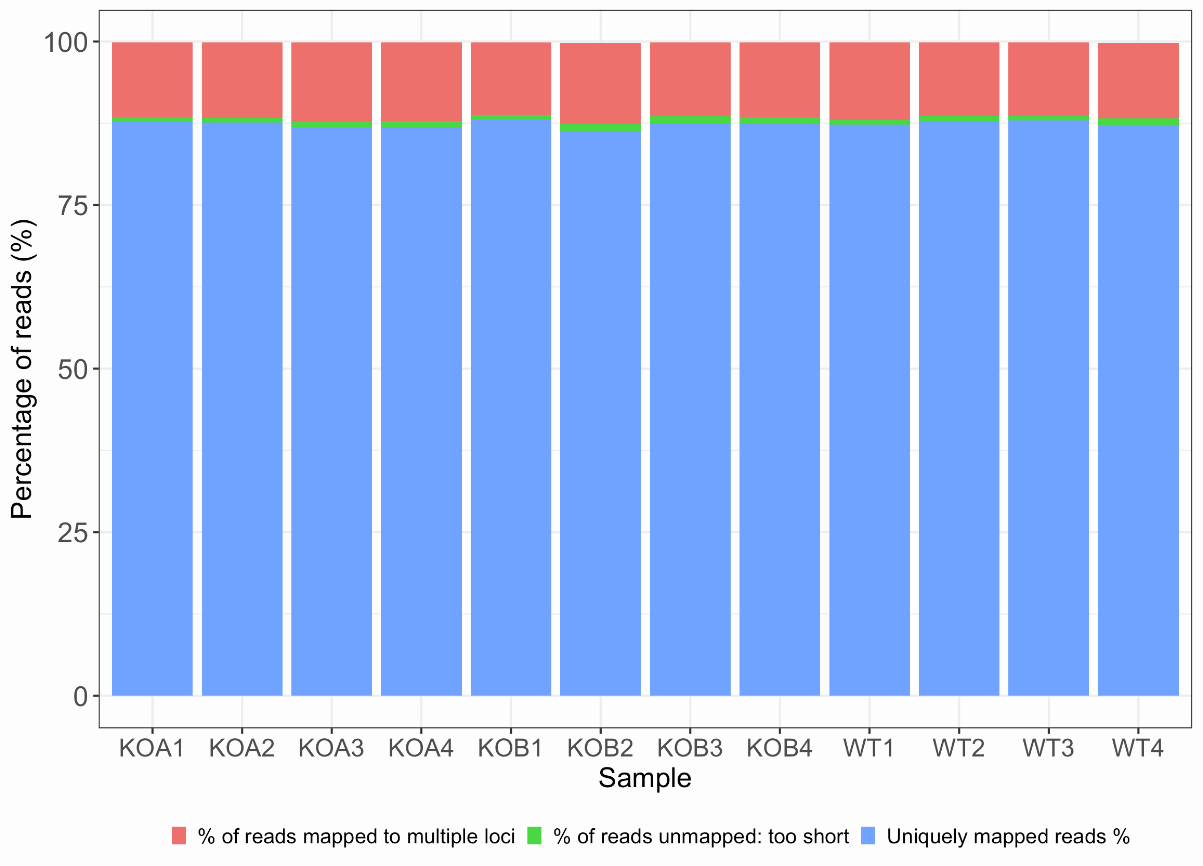


**Figure S4. Alignment statistics for the RNA-seq analysis.** The percentage of reads (y-axis) per sample (x-axis) aligning to either unique positions (blue), multiple loci (red) or no loci due to being too short (green) on the GRCh38/hg38 reference genome.

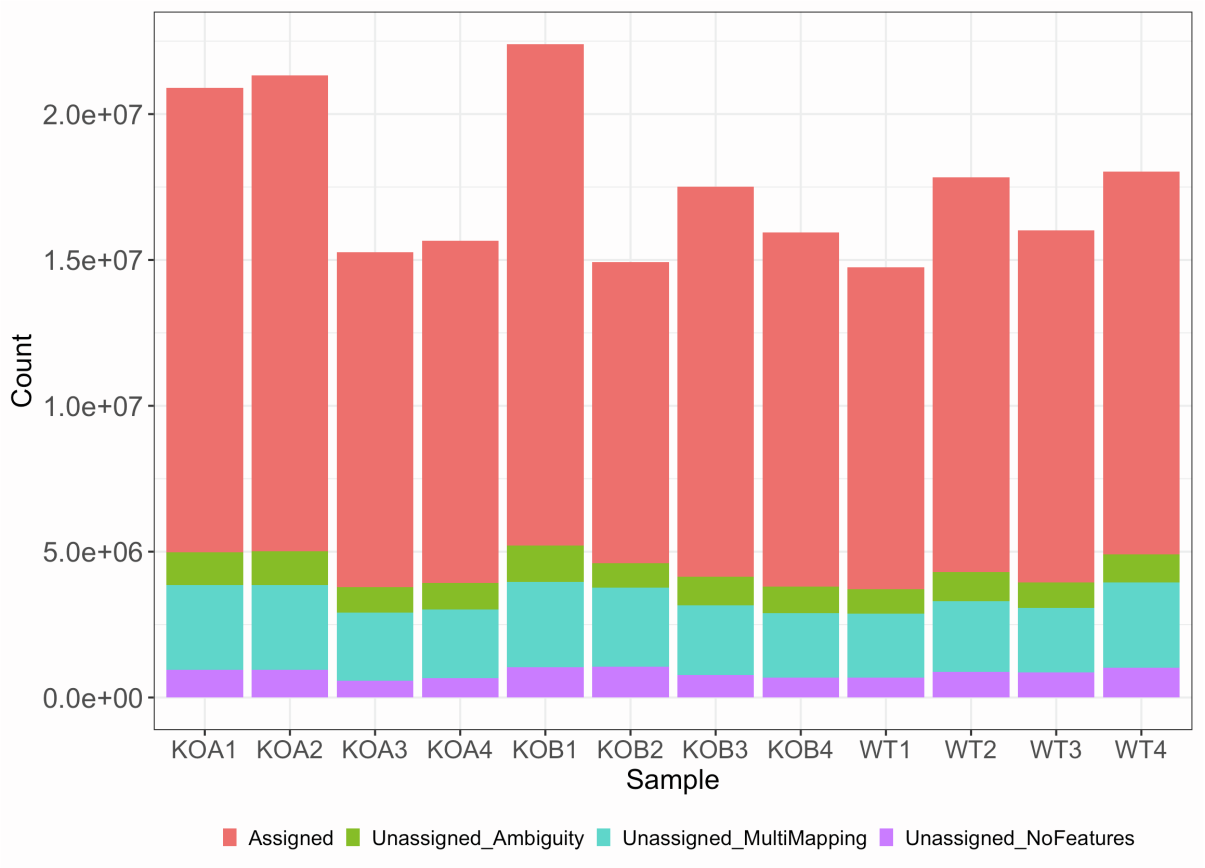


**Figure S5. Gene Quantification Plot.** The number of reads (y-axis) per sample (x-axis) being assigned to genomic features (exons). The red blocks represent reads that reads that were successfully assigned to a genomic feature. The green blocks represent reads unassigned to a genomic feature due to ambiguity. The turquoise blocks represent reads unassigned to a genomic feature due to multi-mapping. The purple blocks represent reads unassigned to a genomic feature due to not overlapping any genomic feature.

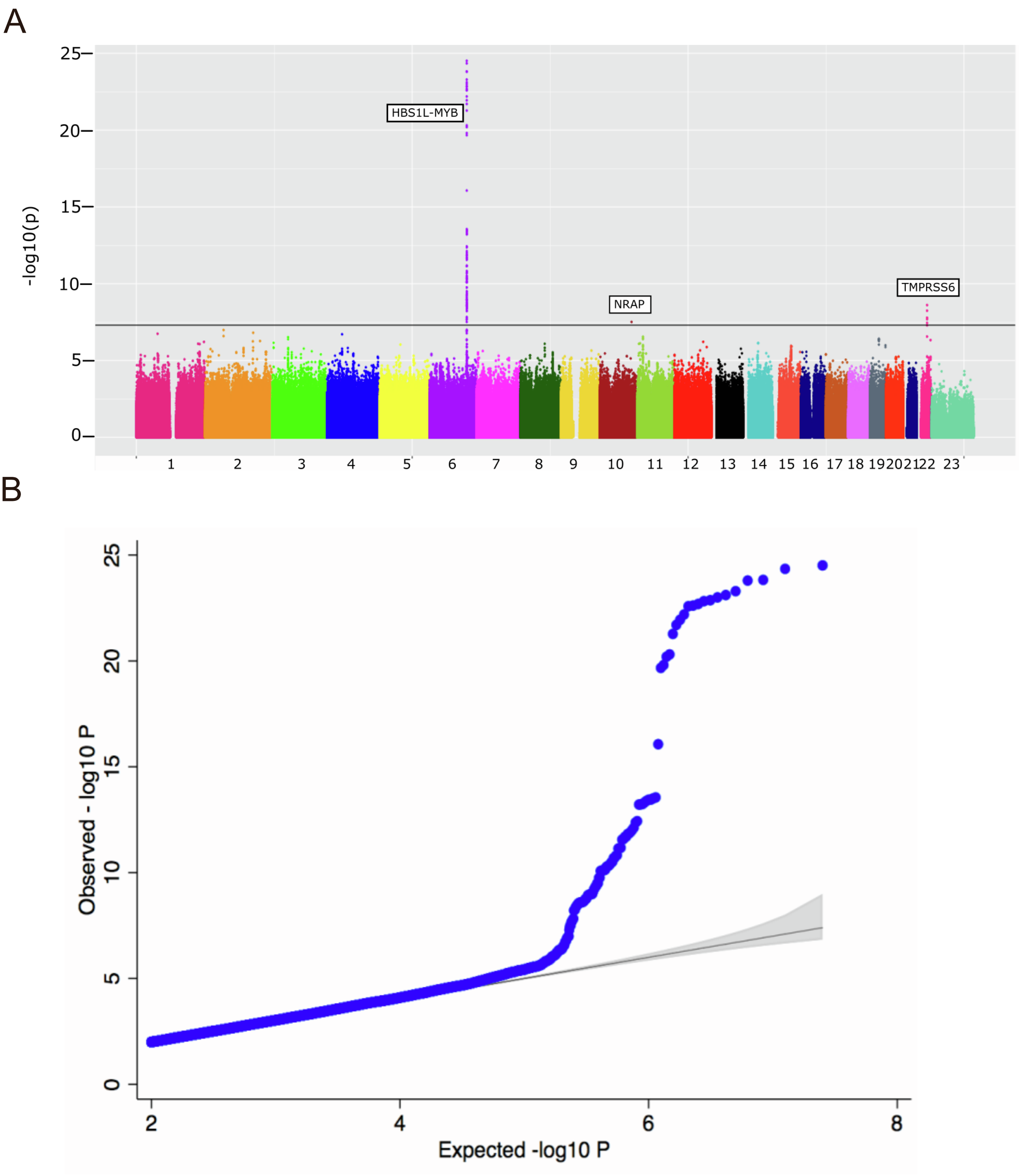


**Figure S6. Genome-wide association study of endogenous EPO in 6,127 individuals. A:** Manhattan Plot of results from genome-wide association study meta-analysis of circulating EPO levels in 6,127 individuals of European and African American descent. Meta-analysis was performed on genome-wide summary statistics from four independent studies using a fixed-effects inverse-variance weighted models. Black solid line indicates p value threshold for genome-wide significance ($p < 5 \times 10^{-08}$). Conditionally independent genomic loci passing genome-wide significance, as identified through conditional analysis, are labelled. Each dot represents an individual marker. Results are plotted by chromosome and position (x-axis). **B:** Quantile-Quantile plots of p values from genome-wide association study

meta-analysis in 6,127 individuals of European and African descent. The areas shaded in grey are 95% confidence bands for the black diagonal line (i.e. the expected distribution of p values). Points to the left of the diagonal line represent associations that are more significant than expected

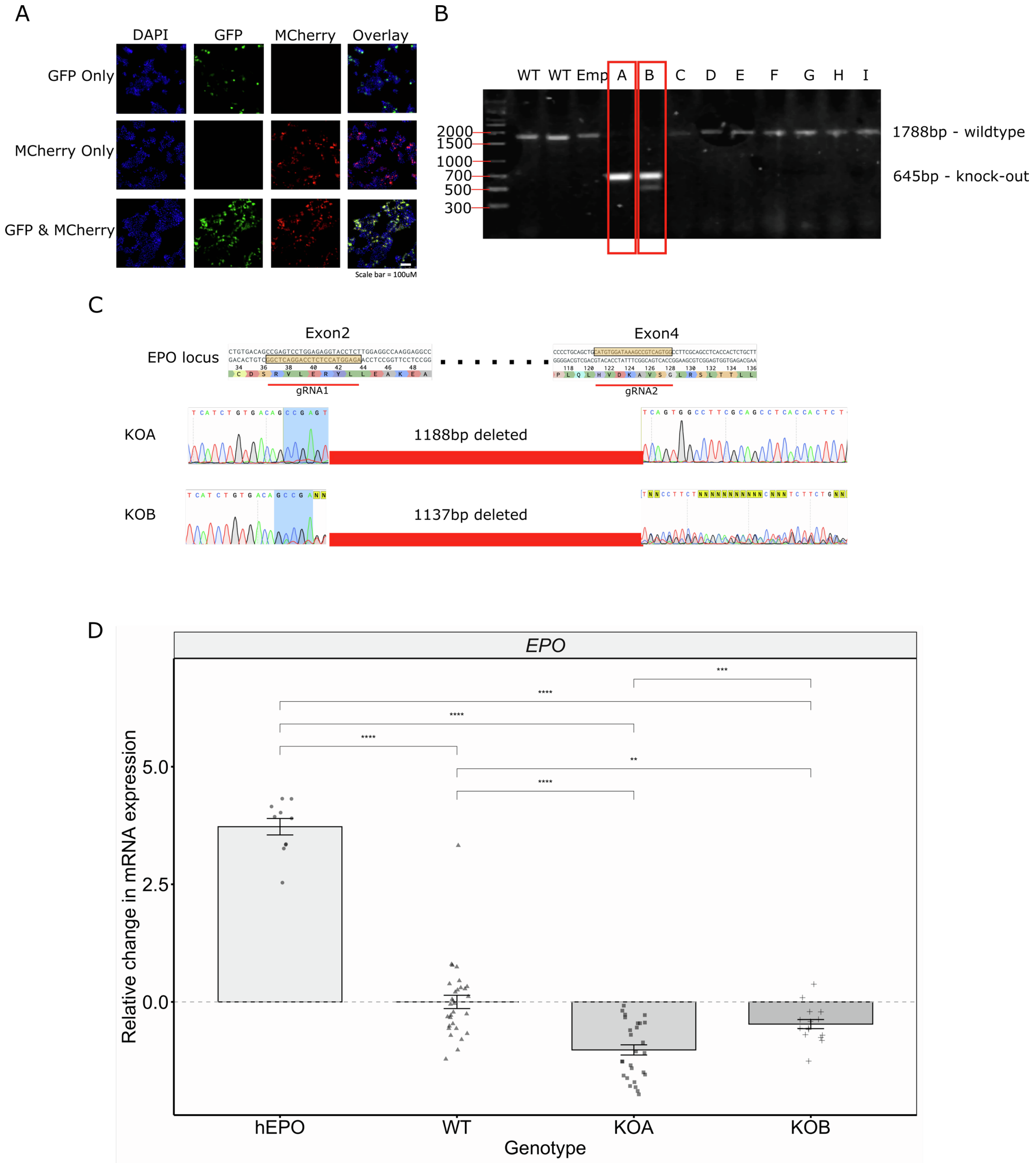


**Figure S7. Establishment of whole *EPO* gene knock-out cell-lines in HEK-293 cells using CRISPR-Cas9 gene-editing technology with paired gRNA approach.** **A)** Fluorescence microscopy images of HEK-293 cells to confirm successful transfection of both CRISPR-Cas9 plasmids (mCherry and GFP) containing the paired gRNAs. **B)** PCR Gel electrophoresis image screening CRISPR-Cas9/gRNA treated cell-lines for potential *EPO* knock-out. Cell-lines A and B appeared to be potential *EPO* knock-outs due to the presence of a PCR amplicon of 645 bp as opposed to 1788 bp. WT = wild-type HEK-293 cells, Emp = wild-type HEK-293 cells treated with empty CRISPR-Cas9 vectors (i.e. no gRNAs) used as a negative control. Lane 1 kb ladder. **C)** Sanger sequencing of KOA and KOB to confirm successful disruption of the *EPO* genomic sequence. The expected sequence between the two gRNAs has been deleted from the genome in both KOA and KOB confirming successful gene-editing. **D)** qRT-PCR analysis confirmed a significant reduction in *EPO* mRNA expression levels in KOA and KOB compared to WT cells. Scale bar in A represents 100μM. Data in D is shown as mean ± SEM. Paired t-test was performed. * $p \leq 0.05$, ** $p \leq 0.01$, *** $p \leq 0.001$, **** $p \leq 0.0001$.

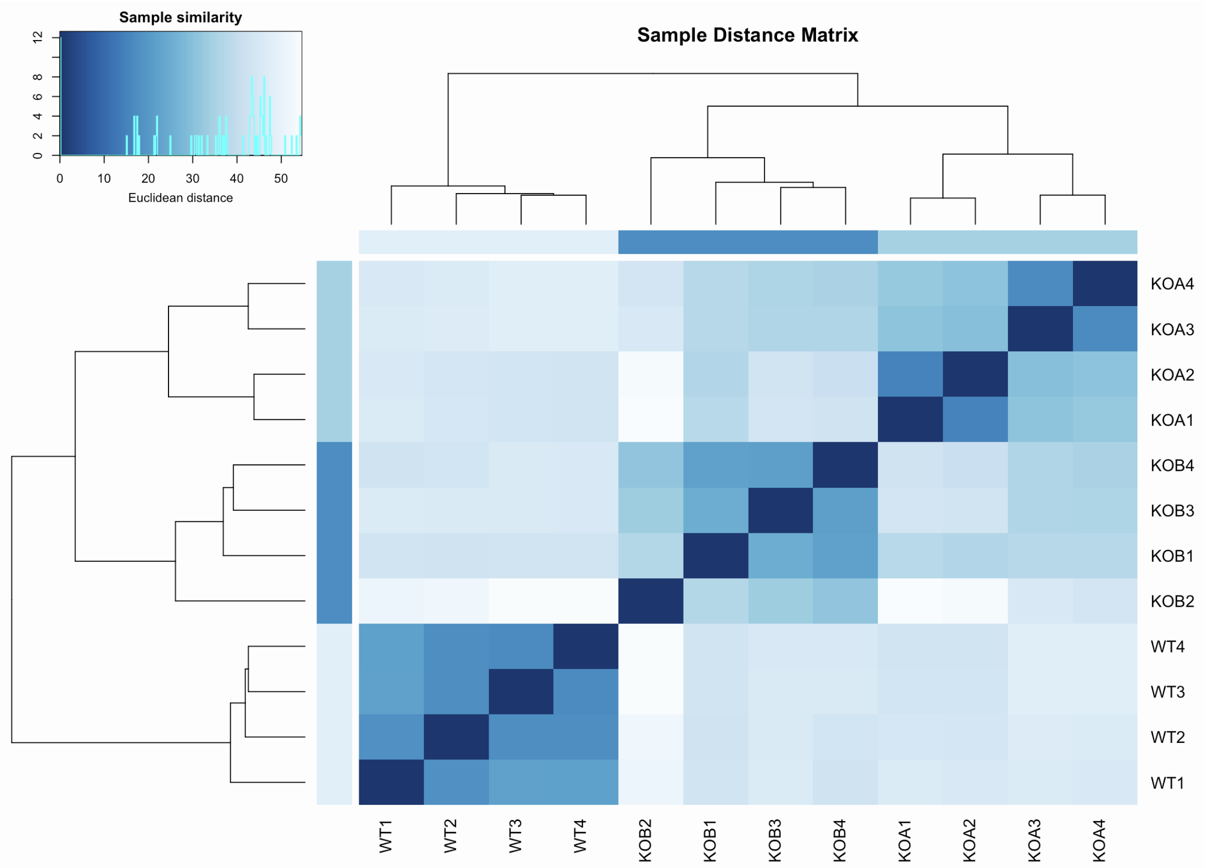


**Figure S8. Distance Matrix Plot illustrating the transcriptomic profile of wild-type HEK293 cells compared to the *EPO*$^{-/-}$ cell-lines.** The color scale represents the distances between samples, where the darker the blue represents the smaller the distance between samples.

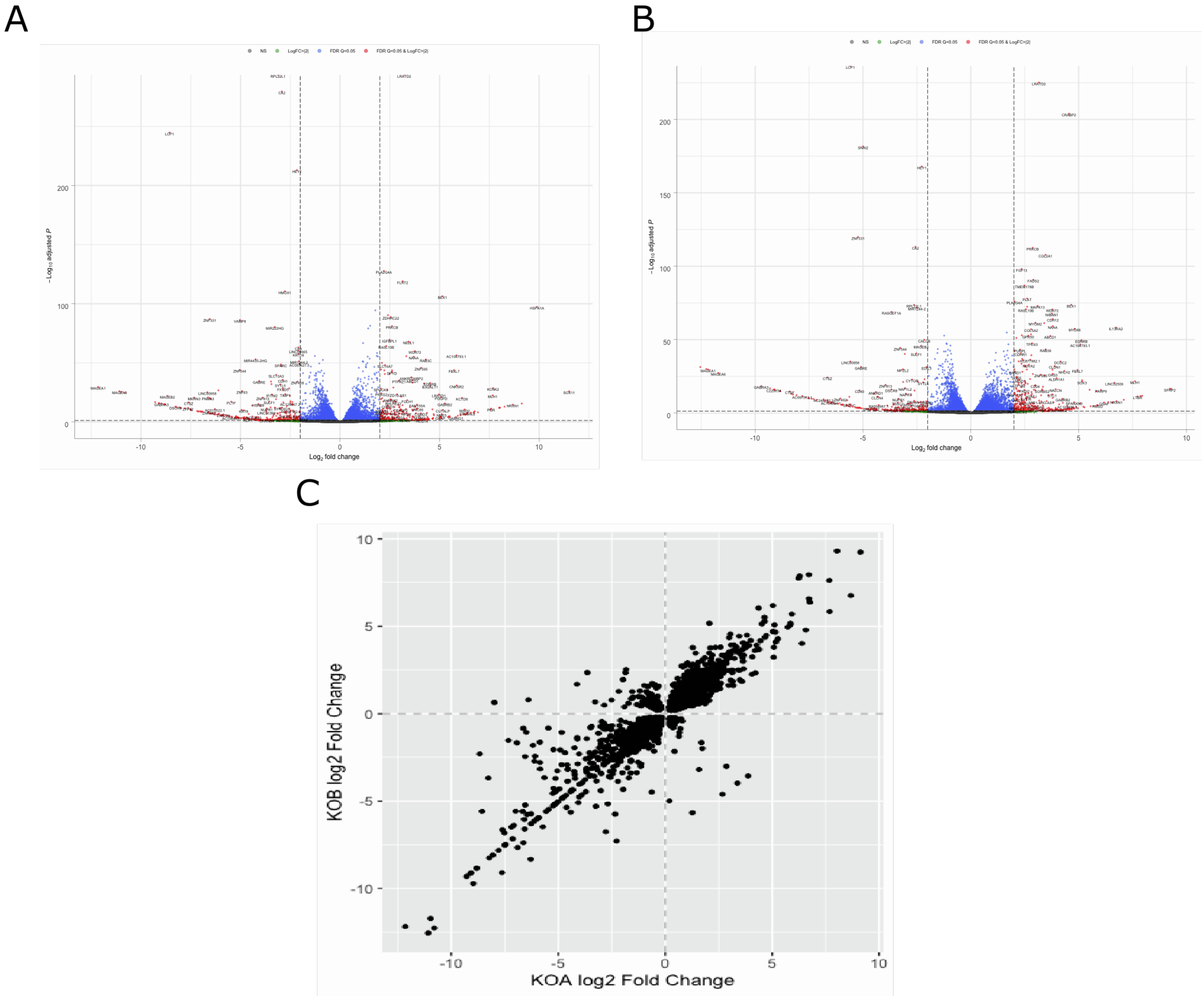


**Figure S9. Differential gene expression analysis of WT HEK-293 cells versus the two *EPO*$^{-/-}$ cell-lines (KOA or KOB). A)** Volcano plot of differential gene expression analysis comparing WT to KOA. **B)** Volcano plot of differential gene expression analysis comparing WT to KOB. The x-axis shows the $log_2$ fold-change and the y-axis represents the $-log_{10}$ adjusted p value. The vertical dashed lines represent a $log_2$ fold-change > |2| and the horizontal lines represent an adjusted p value ≤ 0.05. The red circles represent genes with an adjusted p value ≤ 0.05 and a $log_2$ fold-changes > |2|, the blue circles represent genes with an adjusted p value ≤ 0.05, the green circles represent genes with a $log_2$ fold-change > |2|. Black dots represent all gene that did not reach statistical significance (i.e. had a $log_2$ fold-change < |2| or an adjusted p > 0.05). **C)** Comparison of the $log_2$ fold-changes of the 3,722 overlapping DEGs ($p \leq 0.05$) identified from WT vs KOA (x-axis) differential gene expression analysis and the WT vs KOB (y-axis) differential gene expression analysis. Pearson's correlation coefficient, $r^2 = 0.90$, $p < 2.2 \times 10^{-16}$. The 3,501 DEGs with consistent $log_2$ fold-changes were taken forward for further analysis.

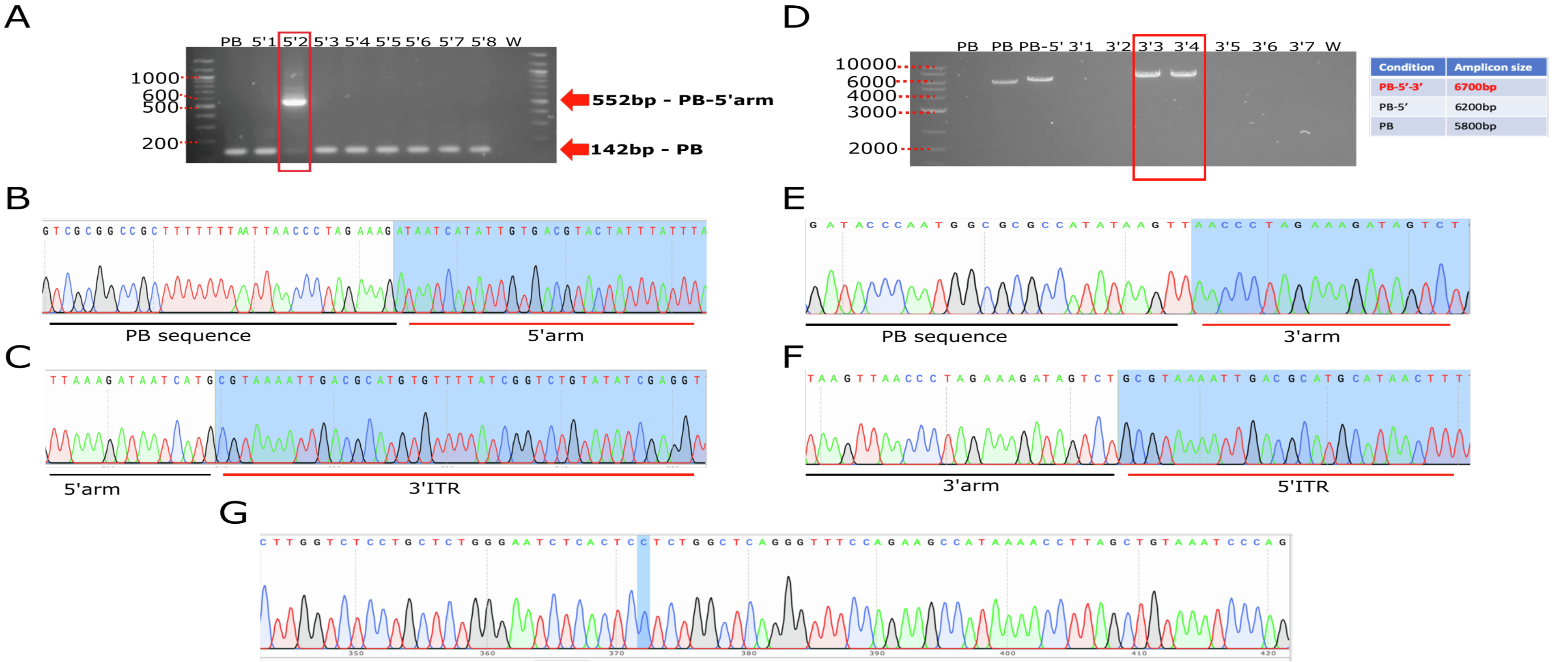


**Figure S10. Cloning of the 5’ and 3’ homology arms into the *piggyBac™* plasmid. A)** PCR Gel electrophoresis to screen *piggyBac™* plasmids for successful cloning of the 5’ homology arm upstream of the 3’ITR region. Plasmid 5’2 contained the 5’ homology arm inserted into *piggyBac™* plasmid. Lane 1: 100 bp ladder. **B)** Sanger sequencing to confirm successful integration of the 5’ homology arm within the correct location downstream of the plasmid sequence in the *piggyBac™* backbone plasmid. **C)** Sanger sequencing to confirm successful integration of the 5’ homology arms upstream of the 3’ITR in the *piggyBac™* backbone plasmid. **D)** Gel electrophoresis of *NsiI* digested *piggyBac™* plasmids to screen for *piggyBac™* plasmids which contain the 5’homology arm and the 3’homology arm. Digested plasmid 3’3 and 3’4 appear to have both the 5’ and 3’ homology arms. Lane 1: 1 kb ladder. **E)** Sanger sequencing to confirm successful integration of the 5’ homology arm within the correct location upstream of the *piggyBac™* plasmid sequence. **F)** Sanger sequencing to confirm successful integration of the 5’ homology arm downstream of the 5’ITR in the *piggyBac™* backbone plasmid. **G)** Sanger sequencing to confirm the desired single base gene-edit (A > C at variant rs1617640) within the 5’ homology arm. PB: empty *piggyBac™* plasmid; W: $ddH_20$; PB-5’: *piggyBac™* plasmid with the 5’arm inserted.

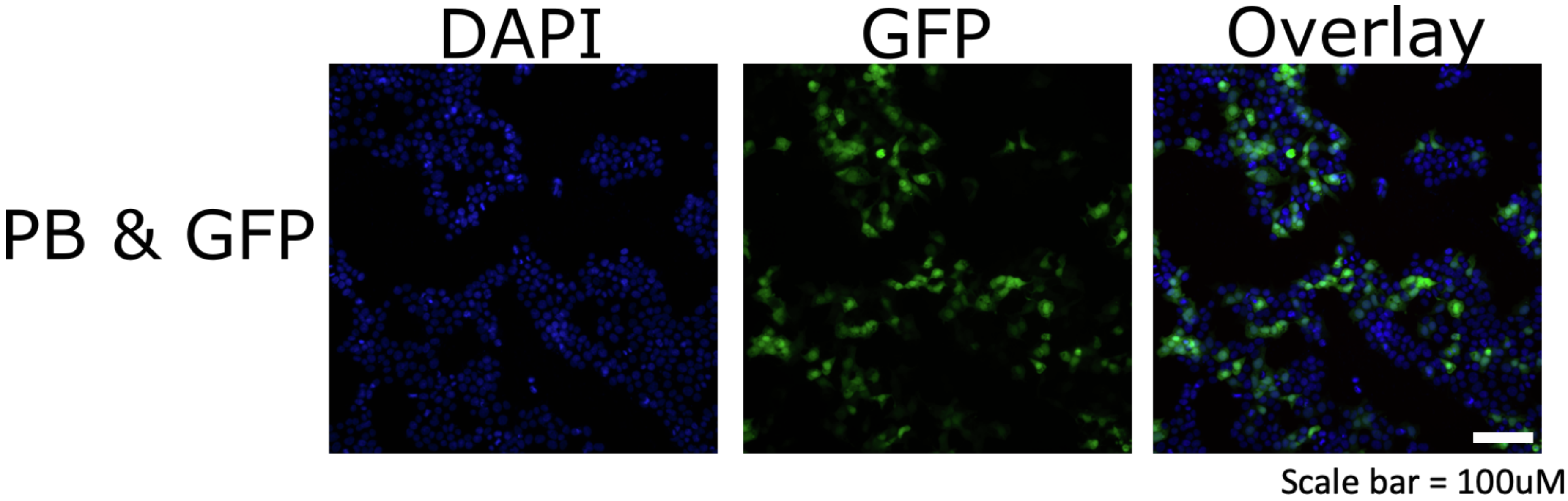


**Figure S11. Fluorescent microscopy images confirming successful transfection of the CRISPR-Cas9-GFP plasmid and the *piggyBac™* plasmid into HEK-293 cells.** Scale bar represents 100μM.

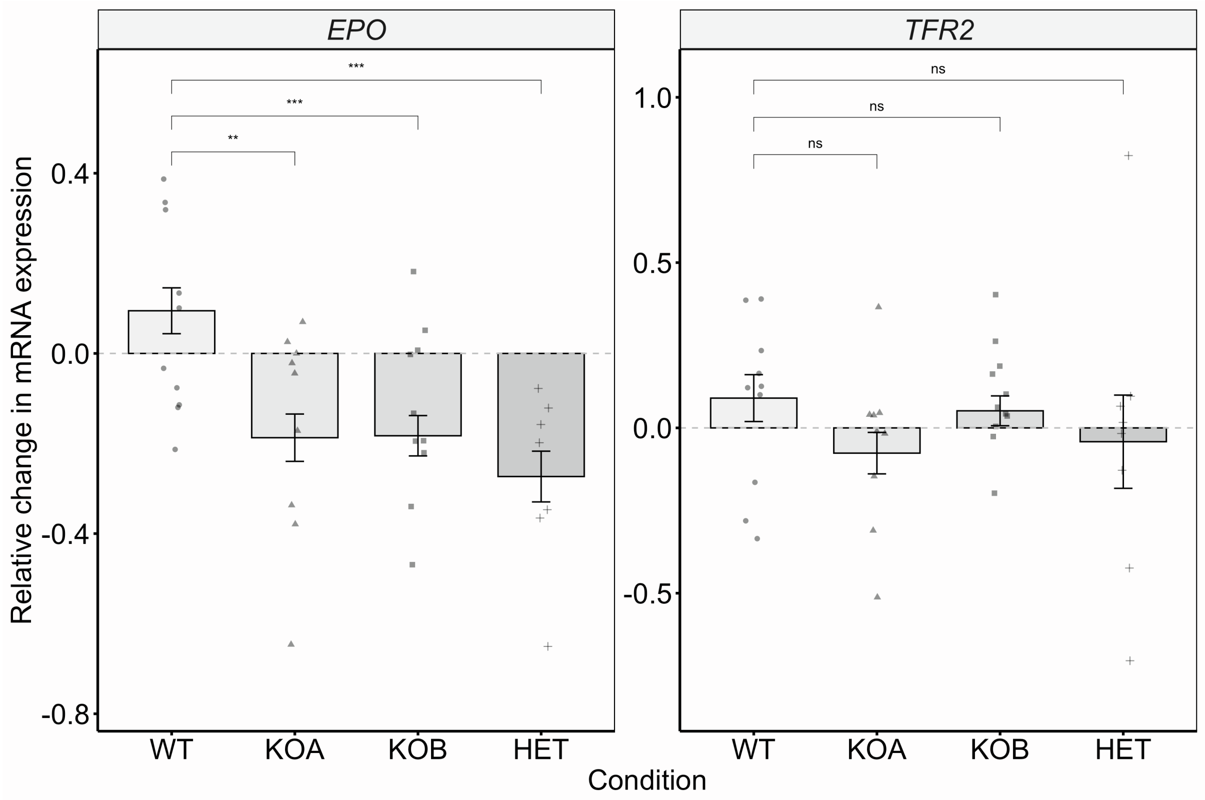


**Figure S12. Investigation of the effect of disruption to the *EPO* gene on *TFR2* mRNA expression.** qRT-PCR showed no difference in *TFR2* mRNA expression in *EPO*$^{-/-}$ knock-outs (KOA or KOB) or heterozygotes for the A-allele at the *cis-EPO* SNP (HET) compared to wild-type control cells homozygote for the A-allele at the *cis-SNP* (WT). Data is shown as mean ± SEM. Paired t-test was performed. * p ≤ 0.05, **p ≤ 0.01, *** p ≤ 0.001, ns=non-significant.

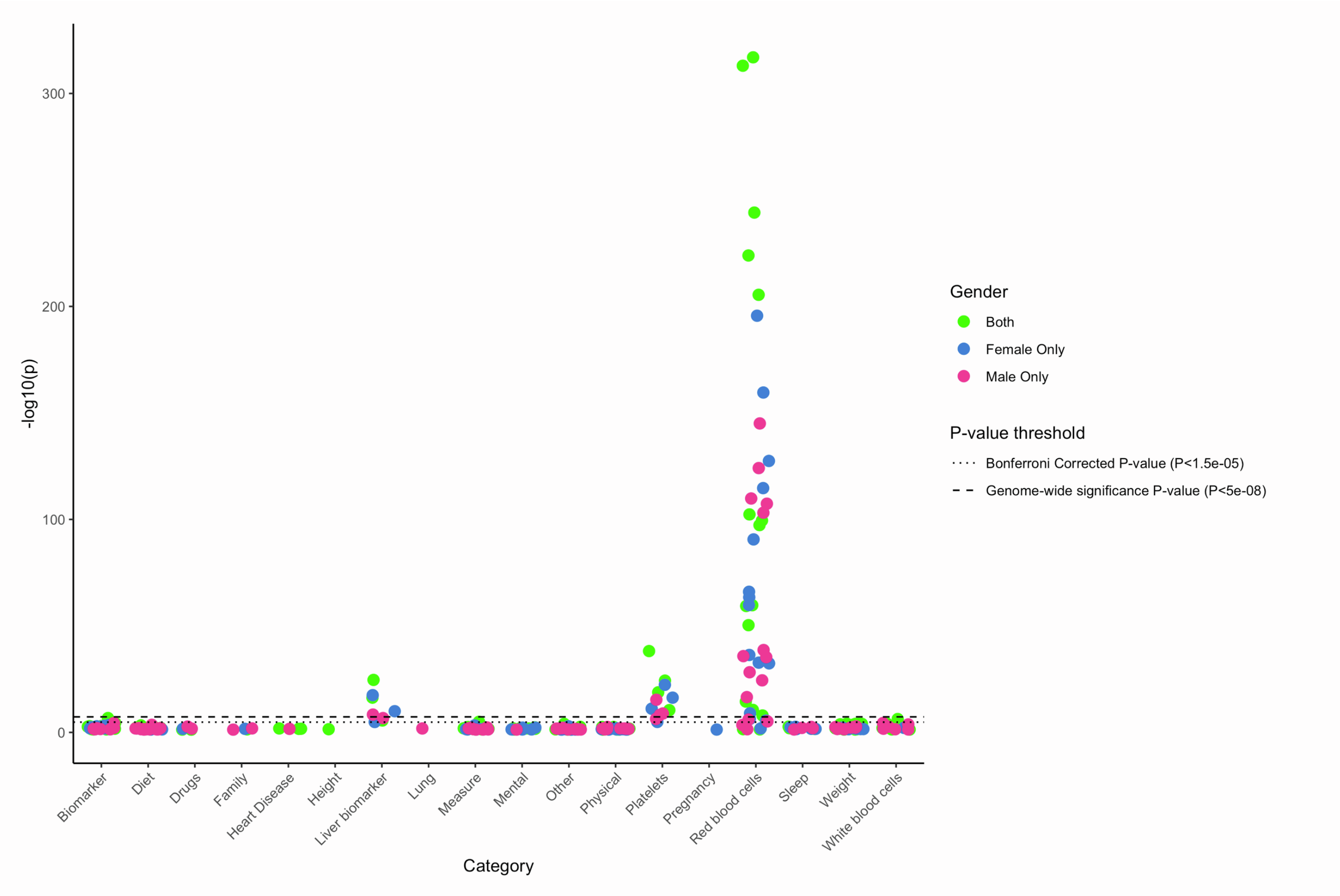


**Figure S13. PheWAS of the *cis*-EPO SNP with 869 traits in up to 451,099 individuals from UK Biobank.** Plot represents the $-\log_{10}$ p values (y-axis) for all traits passing a p value threshold of 0.05. Analysis was performed in both males and females combined (green dots), females only (blue dots) and males only (pink dots). The dotted line highlights associations passing a Bonferroni corrected p value < 1.5 x $10^{-05}$ and the dashed line highlights associations passing genome-wide significance p value < 5 x $10^{-08}$. Traits have been clumped together into categories which are represented on the x-axis.

## Supplemental Tables

**Table S1. Conditionally independent SNPs associated with (at p < *5E-08)* circulating EPO levels in meta-analysis of 6,127 individuals of European and African American descent.**

| Lead SNP | Chromosome | Position | Genomic Locus | Effect allele | EAF | Effect size | SE | p value | Sample size |
|---|---|---|---|---|---|---|---|---|---|
| rs4895441 | 6 | 135426573 | HBS1L | A | 0.74 | -0.24 | 0.021 | 1.45E-30 | 6127 |
| rs112631630 | 10 | 115407228 | NRAP | A | 0.996 | 3.598 | 0.65 | 3.09E-08 | 536 |
| rs855791 | 22 | 37462936 | TMPRSS6 | A | 0.42 | 0.113 | 0.019 | 2.47E-09 | 6529 |

**Table S2. Study characteristics of the four independent cohorts included in EPO meta-analysis.**

| Cohort | Sample size | % Men | Mean Age/years (SD) | Mean EPO/ IU/L (SD) | Mean hemoglobin/ g/dL (SD) | Mean eGFR/ mL/min/1.73m$^2$ (SD) | Software GWAS implemented in | Covariates adjusted for | Other sample exclusion criteria based on GWAS data |
|---|---|---|---|---|---|---|---|---|---|
| InCHIANTI | 1210 | 44.63 | 66.7(15.3) | 9.7 (5.1) | 14.1 (1.1) | 75.8 (16.0) | GEMMA 0.94.1 | Age and Sex | Genotype or phenotype missing data |
| PREVEND | 2954 | 51.76 | 53.69(11.92) | 9.03 (14.94) | 13.76(1.23) | 80.95 (13.95) | SNPtest v2.5.4 | Age, Sex, 10 Pcs | Genotype or phenotype missing data, sex mismatch, <95% call rate, PC outliers. |
| HealthABC Europeans | 969 | 51.7 | 73.678 (2.772) | 12.937 (6.467) | 14.161 ( 1.081 ) | 78.458 ( 15.953 ) | Rvtests 2.1.0 | Age, Sex, Study site, and PCs | Excess heterozygosity, missingness > 5%, sex mismatch, population |

| | | | | | | | | | |
|---|---|---|---|---|---|---|---|---|---|
| | | | | | | | | | outliers, and related individuals were excluded by a grm cut-off of 0.125 (no closer than cousin) |
| Health ABC African Americans | 536 | 41.2 | 73.254 (2.858) | 13.626 (1.014) | 13.626 (1.014) | 88.332 (20.832) | Rvtests 2.1.0 | Age, Sex, Study site, and PCs | Excess heterozygosity, missingness > 5%, sex mismatch, population outliers, and related individuals were excluded by a grm cut-off of 0.125 (no closer than cousin) |
| BLSA | 458 | 50 | 69.1 (13.6) | 15.2 (1.48) | 14.0 (1.1) | 72.1 (13.8) | GEMMA 0.94.1 | Age, sex and PCs | Phenotype or GWAS missing data |

**Table S3. Association between rs1617640 and EPO and TFR2 expression in human liver.** The data was coded such that a negative beta (tmeta) means that as the number of minor alleles (C-alleles) increases there is a decrease in *EPO* or *TFR2* expression.

| Dataset | Sample Size | Expression | Genotyping | PMID | *EPO* | | | *TFR2* | | |
|---|---|---|---|---|---|---|---|---|---|---|
| | | | | | **Beta** | **SE** | **p value** | **Beta** | **SE** | **p value** |
| Dataset1 | 161 | Agilent-014850 Whole Human Genome. 4x44K gene expression (NCBI GEO accession: GSE25935) | Illumina Human610-Quad v1.0 BeadChip (NCBI GEO accession: GSE26105) | 21637794 | 0.0592 | 0.1008 | 0.558 | 0.0827 | **1.56 E-13** | 0.3036 |
| | | 4x44K gene expression | (NCBI GEO accession: GSE26105) | | | | | | | |
| | | (NCBI GEO accession: GSE25935) | | | | | | | | |
| Dataset2 | 145 | Illumina Human Whole Genome-6 v2.0 (NCBI GEO accession: GSE32504) | Illumina HumanHap300-Duo v2.0 Genotyping (NCBI GEO accession: GSE39036) | 18462017 | 0.1048 | 0.0989 | 0.2911 | 0.2653 | 0.0729 | 3.98 E-4 |
| | | (NCBI GEO accession: GSE32504) | (NCBI GEO accession: GSE39036) | | | | | | | |

| | | | | | | | | | | |
|---|---|---|---|---|---|---|---|---|---|---|
| Dataset3 | 555 | Agilent Technologies (NCBI GEO accession: GSE9588) | HumanHap 650Y | 21602305 | 0.2842 | 0.0492 | 1.35 E-8 | 0.246 | 0.0367 | 5.43 E-11 |
| | | (NCBI GEO accession: GSE9588) | | | | | | | | |
| **Meta-analysis** | **861** | | | | **$T_{mets}$ = 5.39** | **0.1488** | **6.86 E-8** | **$T_{mets}$ = 7.38** | **0.1488** | **1.56 E-13** |

**Table S4. Association between rs1617640 and *EPO* and *TFR2* expression in human kidneys.** The data was coded such that a negative beta (tmeta) means that as the number of minor alleles (C-alleles) increases there is a decrease in *EPO* or *TFR2* expression.

| **Dataset** | **Sample size** | **Tissue** | ***EPO*** | | | | ***TFR2*** | | | |
|---|---|---|---|---|---|---|---|---|---|---|
| | | | **T-statistic** | **Beta** | **SE** | **p value** | **T-statistic** | **Beta** | **SE** | **p value** |
| Kidney TransplantLines [2] | 286 | Kidney | 0.117 | 0.157 | 1.338 | 0.907 | 1.358 | 1.333 | 0.981 | 0.177 |

**Table S5. List of primers used in qRT-PCR.**

| Gene | Primer name | Sequence (5’-3’) |
|---|---|---|
| *EPO* | EPO-qpcr_fwd | CCTTCGCAGCCTCACCACT |
| *EPO* | EPO-qpcr_rev | TGTACAGCTTCAGCTTTCCCC |
| *GAPDH* | GADPH_qpcr_fwd | TCCTCTGACTTCAACAGCGAC |
| *GAPDH* | GADPH_qpcr_rev | GCTGTAGCCAAATTCGTTGTCA |
| *IFITM2* | IFITM2_qpcr_fwd | TTCATGAACACCTGCTGCCT |
| *IFITM2* | IFITM2_qpcr_rev | AGATGTTCAGGCACTTGGCG |
| *LRATD2* | LRATD2_qpcr_fwd | GCCGAGCCTACACCTTCAAA |
| *LRATD2* | LRATD2_qpcr_rev | CGAAACCAACTCCAGGGTCA |
| *MLH1* | MLH1_qpcr_fwd | GAAGTTATCCAGCGGCCAG |
| *MLH1* | MLH1_qpcr_rev | TGAATCAACTTCAGGCCTCC |
| *ZNF331* | ZNF331_qpcr_fwd | GGTCTCACTGGATTTGGAGT |
| *ZNF331* | ZNF331_qpcr_rev | AGCGTACCTTCACATATCCAG |
| *HEY1* | HEY1_qpcr_fwd | TGCGGACGAGAATGGAAACT |
| *HEY1* | HEY1_qpcr_rev | TCGTCGGCGCTTCTCAATTA |
| *HEY2* | HEY2_qpcr_fwd | GCAACAGGGGGTAAAGGCTA |
| *HEY2* | HEY2_qpcr_rev | CTTCCACGGAGCTCAGGTAC |
| *HES1* | HES1_qpcr_fwd | AAGAAAGATAGCTCGCGGCA |
| *HES1* | HES1_qpcr_rev | TACTTCCCCAGCACACTTGG |
| *NOTCH1* | NOTCH1_qpcr_fwd | CGCACAAGGTGTCTTCCAG |
| *NOTCH1* | NOTCH1_qpcr_rev | AGGATCAGTGGCGTCGTG |
| *PARP9* | PARP9_qpcr_fwd | GGCAAAGAGGTCCAAGATGCTG |
| *PARP9* | PARP9_qpcr_rev | GCCTCATACATCTCTTCCACGT |
| *DTX3L* | DTX3L_qpcr_fwd | AGAGCTCTAAGTCCTCGGGC |

| | | |
|---|---|---|
| *DTX3L* | DTX3L_qpcr_rev | ACTCTCTCCTTAGCTGCCCT |
| *VEGFR3* | VEGFR3_qpcr_fwd | TGCACGAGGTACATGCCAAC |
| *VEGFR3* | VEGFR3_qpcr_rev | GCTGCTCAAAGTCTCTCACGA |
| *TFR2* | TFR2_qpcr_fwd | TTTCCACCAGGGCAGACTCT |
| *TFR2* | TFR2_qpcr_rev | TGGTTTGCCTGATGGTGTCC |
| *POLR2A* | POLR2A_qpcr_fwd | CCATCAAGAGAGTCCAGTTCG |
| *POLR2A* | POLR2A_qpcr_rev | ACCCTCCGTCACAGACATTC |
| *PPIA* | PPIA_qpcr_fwd | TTCATCTGCACTGCCAAGAC |
| *PPIA* | PPIA_qpcr_rev | TCGAGTTGTCCACAGTCAGC |
| *UBC* | UBC_qpcr_fwd | ATTTGGGTCGCGGTTCTTG |
| *UBC* | UBC_qpcr_rev | TGCCTTGACATTCTCGATGGT |

**Table S6. Comprehensive gene stability of the tested housekeeping genes for use as a reference gene in qRT-PCR.**

| Genes | Geomean of ranking values | Stability |
|---|---|---|
| *GAPDH* | 1.41 | Most stable |
| *Pol2Rα+GAPDH* | 1.57 | |
| *GAPDH+UBC* | 2.63 | |
| *Pol2Rα* | 3.66 | |
| *UBC* | 4.73 | |
| *Pol2Rα+GAPDH+UBC* | 6 | Least stable |

**Table S7. List of primers used in the establishment of the rs1617640 knock-in model using CRISPR-Cas9 and the *piggyBac* system.**

| Primer Name | Function of primer | Sequence (5'-3') |
|---|---|---|
| F1 | Genotyping | CTGGTAGTTTCACCACACCCA |
| R1 | Genotyping | TTGGGCGGAGACTCAGAGAT |
| epo_snp-forward | Genotyping | CTGAATGGGATAGGCTGGTAGT |
| PB1 | Genotyping | CGTCAATTTTACGCATGATTATCTTTAAC |
| F2 | Genotyping | TGAGCCACCACACCTGACTA |
| R2 | Genotyping | TTCTTCCTCCCCACCTCACT |
| PB-F | Detect transposon re-integration | GGCATAGTATATCGGCATAG |
| PB-R | Detect transposon re-integration | GTTAGAAGACTTCCTCTGC |
| M13_F | Insertion of homology arm into piggyBac plasmid | TGTAAAACGACGGCCAGT |
| 3ITR_R | Insertion of homology arm into piggyBac plasmid | CGTCAATTTTACGCATGATTATCTTTAAC |
| 5ITR_F | Insertion of homology arm into piggyBac plasmid | GCGACGGATTCGCGCTATTTAGAAAG |
| M13_R | Insertion of homology arm into piggyBac plasmid | CAGGAAACAGCTATGACCATG |
| 5arm_seq | Sequencing of 5' homology arm & 3' ITR | AGACTGCCTTGGGAAAAG |
| 3arm_seq | Sequencing of 3' homology arm & 5'ITR | GCATTCTAGTTGTGGTTTGTCC |

**Table S8. rs1617640-outcome association statistics.** All effects have been aligned to the EPO-increasing allele (A).

| **Disease Outcomes** | | | | | | | | | |
|---|---|---|---|---|---|---|---|---|---|
| **Outcome** | **Study** | **A1** | **A2** | **A1 freq** | **OR** | **Lower 95%** | **Upper 95%** | **p value** | **N (Cases/Controls)** |
| CAD | UK Biobank | A | C | 0.6 | 1.001 | 0.986 | 1.016 | 0.82 | 37741 / 318892 |
| CAD | Nikpey *et al.* (2015) | A | C | 0.59 | 1.005 | 0.985 | 1.025 | 0.643 | 60801 / 123504 |
| | **Meta-analysis** | | | | **1.002** | **0.99** | **1.01** | **0.72** | **98542 / 442396** |
| MI | UK Biobank | A | C | 0.6 | 0.99 | 0.964 | 1.018 | 0.48 | 10590 / 440509 |
| MI | Nikpay *et al.* (2015) | A | C | 0.57 | 1.004 | 0.983 | 1.026 | 0.706 | 42561 / 123504 |
| | **Meta-analysis** | | | | **0.999** | **0.98** | **1.02** | **0.889** | **53151 / 564013** |
| Stroke | UK Biobank | A | C | 0.6 | 0.963 | 0.935 | 0.993 | 0.014 | 9092 / 346423 |
| Stroke | Malik *et al.* (2018) | A | C | 0.6 | 1.01 | 0.993 | 1.031 | 0.344 | 40585 / 406111 |
| | **Meta-analysis** | | | | **0.995** | **0.98** | **1.01** | **0.553** | **49677 / 752534** |
| **Continuous Outcomes** | | | | | | | | | |
| **Outcome** | **Study** | **A1** | **A2** | **A1 freq** | **Beta (95% CI)** | **Lower 95%** | **Upper 95%** | **p value** | **N** |

| | | | | | | | | | |
|---|---|---|---|---|---|---|---|---|---|
| SBP | UK Biobank | A | C | 0.6 | 0.04 | -0.05 | 0.12 | 0.8 | 450075 |
| SBP | Wain *et al.* (2017) | A | C | 0.59 | 0.024 | -0.11 | 0.16 | 0.725 | 228245 |
| | **Meta-analysis** | | | | **0.03** | **-0.04** | **0.11** | **0.38** | **678320** |
| DBP | UK Biobank | A | C | 0.6 | -0.07 | -1.20E-01 | -0.02 | 1.00E-05 | 449322 |
| DBP | Wain *et al.* (2017) | A | C | 0.59 | -0.03 | -0.11 | 0.05 | 0.469 | 228245 |
| | **Meta-analysis** | | | | **-0.06** | **-0.1** | **-0.02** | **0.006** | **677567** |
| Heart rate | UK Biobank | A | C | 0.6 | -0.07 | -0.13 | -0.02 | 0.019 | 423846 |
| Heart rate | den Hoed *et al.* (2013) | A | C | 0.59 | -0.021 | -0.13 | 0.09 | 0.703 | 90849 |
| | **Meta-analysis** | | | | **-0.06** | **-0.11** | **-0.02** | **0.01** | **514695** |

**Table S9. Genome-wide significant association of rs4895441 and rs855791, two of the three lead genetic variants identified in the EPO meta-analysis, with other phenotypes in up to 451,099 UK Biobank unrelated, European individuals.** Effect estimates are aligned to the EPO-increasing allele.

| Genetic variant | Phenotype | Gender | Beta | SE | p value |
|---|---|---|---|---|---|
| **rs4895441** | Erythropoietin | Combined | 0.218 | 0.021 | 1.45E-30 |
| | Corpuscular hemoglobin | Combined | 0.179 | 0.002 | 4.3E-1635 |
| | Corpuscular Volume | Combined | 0.157 | 0.002 | 6.2E-1353 |
| | Mean corpuscular volume | Combined | 0.157 | 0.002 | 6.7E-1270 |
| | Red blood cell count | Combined | -0.139 | 0.002 | 2.3E-1224 |
| | Mean corpuscular volume (anemics excluded) | Combined | 0.163 | 0.002 | 2.7E-1148 |
| | Platelet crit | Combined | 0.12 | 0.002 | 5.4E-790 |
| | Platelet Count | Combined | 0.107 | 0.002 | 1.8E-635 |
| | Fibrosis-4 Score | Combined | -0.079 | 0.002 | 1.9E-399 |
| | Red blood cell distribution width | Combined | -0.092 | 0.002 | 1.3E-386 |
| | Red blood cell distribution width (anemics excluded) | Combined | -0.098 | 0.002 | 4.5E-378 |
| | Non-alcoholic fatty liver disease fibrosis score | Combined | -0.085 | 0.002 | 2.6E-367 |
| | Corpuscular hemoglobin concentration | Combined | 0.075 | 0.002 | 4.70E-261 |
| | Sphered cell volume | Combined | 0.07 | 0.002 | 7.60E-257 |
| | Hematocrit percentage | Combined | -0.062 | 0.002 | 5.30E-255 |
| | Non-alcoholic fatty liver disease fibrosis score | Female | -0.093 | 0.003 | 4.00E-220 |
| | fibrosis-4 Score | Female | -0.082 | 0.003 | 1.30E-214 |
| | Reticulocyte Volume | Combined | 0.065 | 0.002 | 3.60E-209 |
| | fibrosis-4 Score | Male | -0.078 | 0.003 | 1.30E-171 |
| | Non-alcoholic fatty liver disease fibrosis score | Male | -0.077 | 0.003 | 3.00E-141 |

| | | | | | |
|---|---|---|---|---|---|
| | Hemoglobin Concentration | Combined | -0.038 | 0.002 | 1.10E-103 |
| | Reticulocyte Percentage | Combined | 0.046 | 0.002 | 1.50E-101 |
| | Eosinophil Count | Combined | -0.042 | 0.002 | 2.40E-83 |
| | High Light Scatter reticulocyte percentage | Combined | 0.041 | 0.002 | 8.40E-81 |
| | Glycated hemoglobin | Combined | -0.036 | 0.002 | 3.60E-66 |
| | Lymphocyte Count | Combined | -0.034 | 0.002 | 1.00E-52 |
| | Monocyte Count | Combined | -0.031 | 0.002 | 9.70E-51 |
| | Neutrophil Count | Combined | -0.03 | 0.002 | 1.40E-42 |
| | Eosinophil Percentage | Combined | -0.029 | 0.002 | 2.10E-39 |
| | Glycated hemoglobin | Female | -0.035 | 0.003 | 2.10E-35 |
| | Glycated hemoglobin | Male | -0.037 | 0.003 | 2.50E-31 |
| | Cholesterol corrected for statin use | Combined | -0.024 | 0.002 | 7.10E-30 |
| | Low density lipoprotein corrected for statin use | Combined | -0.024 | 0.002 | 7.60E-30 |
| | Cholesterol corrected for statin use | Male | -0.028 | 0.003 | 1.30E-19 |
| | Platelet distribution width | Combined | 0.018 | 0.002 | 2.60E-19 |
| | Aspartate Aminotransferase | Combined | -0.019 | 0.002 | 3.40E-19 |
| | Low density lipoprotein corrected for statin use | Male | -0.027 | 0.003 | 4.30E-18 |
| | Albumin | Combined | 0.02 | 0.002 | 4.00E-17 |
| | Cholesterol | Combined | -0.017 | 0.002 | 9.10E-15 |
| | High Light Scatter Reticulocyte Count | Combined | 0.016 | 0.002 | 2.20E-14 |
| | Low density lipoprotein corrected for statin use | Female | -0.021 | 0.003 | 2.70E-14 |
| | Cholesterol corrected for statin use | Female | -0.021 | 0.003 | 6.40E-14 |
| | Low density lipoprotein | Combined | -0.015 | 0.002 | 2.40E-12 |
| | Aspartate Aminotransferase | Female | -0.021 | 0.003 | 7.70E-12 |
| | Immature reticulocyte | Combined | 0.014 | 0.002 | 6.40E-11 |
| | Microalbumin | Combined | 0.013 | 0.002 | 1.30E-10 |
| | Reticulocyte Count | Combined | 0.013 | 0.002 | 1.60E-10 |

| | | | | | |
|---|---|---|---|---|---|
| | Albumin | Male | 0.022 | 0.004 | 4.60E-10 |
| | Albumin | Female | 0.019 | 0.003 | 2.20E-09 |
| | Apolipoprotein B | Combined | -0.013 | 0.002 | 2.40E-09 |
| | Microalbumin | Female | 0.019 | 0.003 | 3.25E-03 |
| | Cholesterol levels | Male | -0.019 | 0.003 | 3.47E-03 |
| | Aspartate Aminotransferase | Male | -0.019 | 0.003 | 3.49E-03 |
| | High Density Lipoprotein | Male | -0.018 | 0.003 | 3.45E-03 |
| **rs855791** | EPO | Combined | 0.113 | 0.019 | 2.47E-09 |
| | Corpuscular Hemoglobin | Combined | -0.151 | 0.002 | 3.6E-1399 |
| | Corpuscular Volume | Combined | -0.129 | 0.002 | 3.0E-1102 |
| | Mean corpuscular volume | Combined | -0.13 | 0.002 | 9.0E-1038 |
| | Mean corpuscular volume (anemics excluded) | Combined | -0.133 | 0.002 | 2.5E-932 |
| | Red blood cell distribution width (anemics excluded) | Combined | 0.113 | 0.002 | 1.4E-480 |
| | Hemoglobin concentration | Combined | -0.073 | 0.002 | 3.5E-446 |
| | Red blood cell distribution width | Combined | 0.113 | 0.002 | 1.0E-416 |
| | Corpuscular Hemoglobin concentration | Combined | -0.067 | 0.002 | 9.20E-251 |
| | Glycated hemoglobin levels | Combined | 0.064 | 0.002 | 2.00E-244 |
| | Hematocrit Percentage | Combined | -0.053 | 0.002 | 2.10E-224 |
| | Sphered cell volume | Combined | -0.055 | 0.002 | 1.10E-193 |
| | Glycated hemoglobin levels | Female | 0.062 | 0.003 | 8.10E-129 |
| | Glycated hemoglobin levels | Male | 0.065 | 0.003 | 7.10E-111 |
| | Reticulocyte Percentage | Combined | -0.04 | 0.002 | 6.60E-90 |
| | Reticulocyte Count | Combined | -0.035 | 0.002 | 4.70E-72 |
| | Total Bilirubin | Combined | -0.029 | 0.002 | 9.10E-67 |
| | Platelet Count | Combined | 0.027 | 0.002 | 5.00E-52 |
| | Platelet crit | Combined | 0.027 | 0.002 | 7.30E-52 |

| | | | | | |
|---|---|---|---|---|---|
| | Fibrosis-4 score | Combined | -0.022 | 0.002 | 1.60E-38 |
| | High light scatter reticulocyte percentage | Combined | -0.025 | 0.002 | 5.60E-37 |
| | Total Bilirubin | Female | -0.031 | 0.003 | 1.40E-35 |
| | Total Bilirubin | Male | -0.032 | 0.003 | 8.80E-34 |
| | High light scatter reticulocyte count | Combined | -0.022 | 0.002 | 1.80E-28 |
| | Non-alcoholic fatty liver disease fibrosis score | Combined | -0.021 | 0.002 | 3.40E-28 |
| | Platelet distribution width | Combined | -0.019 | 0.002 | 8.90E-24 |
| | Direct Bilirubin | Combined | -0.019 | 0.002 | 4.80E-23 |
| | Red blood cell count | Combined | 0.016 | 0.002 | 2.20E-22 |
| | Fibrosis-4 score | Female | -0.024 | 0.002 | 4.20E-22 |
| | Non-alcoholic fatty liver disease fibrosis score | Female | -0.025 | 0.003 | 1.40E-19 |
| | Direct Bilirubin | Male | -0.023 | 0.003 | 2.20E-16 |
| | Fibrosis-4 score | Male | -0.020 | 0.003 | 2.80E-15 |
| | Immature reticulocytes | Combined | 0.015 | 0.002 | 5.20E-13 |
| | Non-alcoholic fatty liver disease fibrosis score | Male | -0.018 | 0.003 | 1.10E-10 |
| | Phosphate levels | Combined | 0.012 | 0.002 | 1.20E-08 |
| | Direct Bilirubin | Female | -0.014 | 0.003 | 4.00E-08 |

**Table S10.** ***See separate excel sheet.***

**Table S11.** ***See separate excel sheet.***

**Table S12. Two-sample MR, using rs1617640 as an instrumental variable and a meta-analysis of UK Biobank GWAS and previously published, publicly available GWAS, to assess the genetic association between genetically predicted therapeutically altered endogenous EPO levels and risk of CVD or clinical markers for CVD risk factors.**

| Exposure | Outcome | Odds ratio | Lower 95% | Upper 95% | p value | Number of Cases | Number of Controls |
|---|---|---|---|---|---|---|---|
| EPO | CAD | 1.03 | 0.85 | 1.25 | 0.72 | 98542 | 442396 |
| EPO | Stroke | 0.92 | 0.70 | 1.21 | 0.55 | 49677 | 752534 |
| EPO | MI | 0.98 | 0.75 | 1.29 | 0.89 | 53151 | 564013 |
| | **Risk Factors** | **Effect estimate** | **Lower 95%** | **Upper 95%** | **p value** | **Total Sample size** | |
| EPO | SBP | 0.53 | -0.65 | 1.71 | 0.38 | 586080 | |
| EPO | DBP | -0.98 | -1.67 | -0.29 | 0.0057 | 585325 | |
| EPO | Heart rate | -0.996 | -1.74 | -0.25 | 0.0097 | 514706 | |

**Table S13. Rescaling the genetic estimates of EPO levels on adverse cardiovascular risk to the PHI-induced effect on EPO levels (obtained from a recent RCT (Meadowcroft et al., 2019)).**

| Disease | | | | |
|---|---|---|---|---|
| **Exposure** | **Outcome** | **Odds ratio** | **Lower 95%** | **Upper 95%** |
| EPO | CAD | 1.0141892 | 0.9315804 | 1.07268792 |
| EPO | MI | 0.99296952 | 0.86721782 | 1.14986417 |
| EPO | Stroke | 0.96527007 | 0.86721782 | 1.07268792 |
| **Risk Factors** | | | | |
| **Exposure** | **Outcome** | **Effect estimate** | **Lower 95%** | **Upper 95%** |
| EPO | SBP | 0.21155347 | -0.2820713 | 0.77569607 |
| EPO | DBP | -0.4231069 | -0.7051782 | -0.1410356 |
| EPO | Heart rate | -0.4231069 | -0.7756961 | -0.1410356 |

**Table S14. Association of rs1617640 and traits passing genome-wide significance (P < 5E08) obtained from a PheWAS on 869 traits in up to 451,099 European, unrelated, UK Biobank individuals.** All reported effect sizes are aligned to the EPO-increasing A allele of rs1617640.

| Phenotype | Beta | SE | P-value |
|---|---|---|---|
| Red blood cell count | -0.0643 | 0.0017 | 2.3E-315 |
| Corpuscular hemoglobin | 0.0721 | 0.002 | 3.3E-313 |
| Corpuscular volume | 0.0615 | 0.0019 | 8.60E-245 |
| Mean corpuscular volume | 0.0612 | 0.002 | 1.20E-224 |
| Mean corpuscular volume (anemics excluded) | 0.0634 | 0.0021 | 3.50E-206 |
| Red blood cell distribution width | -0.0429 | 0.0021 | 4.50E-103 |
| Red blood cell distribution width (anemics excluded) | -0.0454 | 0.0022 | 3.40E-100 |
| Hematocrit percentage | -0.0349 | 0.0017 | 4.70E-98 |
| Corpuscular hemoglobin concentration | 0.0328 | 0.002 | 1.90E-60 |
| Sphered cell volume | 0.0308 | 0.0019 | 4.60E-60 |
| Hemoglobin concentration | -0.0244 | 0.0016 | 4.40E-51 |
| Platelet Count | 0.0234 | 0.0019 | 6.40E-39 |
| Non-alcoholic fatty acid liver disease fibrosis score | -0.0202 | 0.0019 | 2.20E-25 |
| Platelet volume | -0.0178 | 0.0018 | 4.80E-25 |
| Platelet crit | 0.0165 | 0.0019 | 1.50E-19 |
| Fibrosis-4 score | -0.0141 | 0.0017 | 4.70E-17 |
| Reticulocyte volume | 0.0152 | 0.002 | 3.30E-15 |
| Reticulocyte percentage | 0.013 | 0.002 | 2.70E-11 |
| Platelet distribution width | -0.0124 | 0.0019 | 3.50E-11 |
| High light scatter reticulocyte percentage | 0.0114 | 0.002 | 1.10E-08 |

# Supplemental information

## Supplemental Materials and Methods

### Invecchiare in Chianti (InCHIANTI)

InCHIANTI is a prospective, population-based study of 1,453 individuals aged between 20-102 years (1,156 > 65 years) living in the Chianti region of Tuscany, Italy. Data was collected between 1998 and 2000 and included telephone interviews, medical examinations and blood samples. A detailed description of the study has been described previously[1]. The study was approved by the ethical committee of the Italian National Institute of Research and Care of Aging and complies with the Declaration of Helsinki. All participants received a detailed description of the study purpose and procedures and all signed the informed consent. The present study included 1,210 participants with valid phenotypic and genotypic information.

### Baltimore Longitudinal Study of Aging (BLSA)

BLSA is a longitudinal cohort study conducted by the Intramural Research Program of the National Institute of Aging (NIH) which started in 1958[2]. Healthy volunteers aged between 17 and older are enrolled in the study and participate in follow-up assessment visits of health, physical and psychological performance every 2 years. Currently, the study population has over 3,200 active participants. An independent institutional review board approved the BLSA study protocol, and participants provided informed consent for all analyses included in this report. The present study included 458 patients with valid phenotypic and genotypic data.

### Prevention of Renal and Vascular ENd-stage Disease (PREVEND)

The PREVEND study[3] is a prospective, observational cohort of 8,592 Groningen inhabitants aged between 28-75 years. The main aim of the study is to assess the long-term impact of elevated urinary albumin levels on cardiac- renal- and peripheral vascular end-stage diseases. Upon enrolment, participants agreed to giving a urine sample and answering a questionnaire and are followed up every 2-3 years for a survey on cardiac-, renal- and peripheral vascular morbidity. The PREVEND study

was approved by the medical ethics committee of the University Medical Center Groningen and conducted in accordance with the guidelines of the Declaration of Helsinki. All participants gave written informed consent. The present study included 2,954 individuals with valid phenotypic and genotypic information.

**The Health, Aging and Body Composition Study (HealthABC)**

HealthABC is a prospective, longitudinal study of 3,075 individuals aged between 70-79 between 1997 and 1998 living in Memphis, Tennessee or Pittsburgh. 42% of participants were of African-American ancestry and 52% were of Caucasian ancestry. Participants were enrolled in the study if they had no disabilities, no life-threatening conditions or difficulties walking quarter of a mile and climbing 10 steps. The study consisted of yearly clinical examinations for 6 years, primarily taking measurements of body composition, strength and function, and biannual phone calls to update health status, followed by bi-annual telephone interviews up until Year 16 and examination in Year 16[4]. All respondents provided written informed consent, and all protocols were approved by the institutional review boards at the study sites. The present study included 1,505 individuals with valid phenotypic and genotypic information.

**UK Biobank (UKB) Cohort**

Briefly, UKB recruited more than 500,000 individuals aged 37-73 years between 2006 and 2010 from across the UK[5]. Participants provided a range of information via questionnaires and interviews (e.g. health status, lifestyle) and measurements (anthropometric, blood pressure); this has been described in detail by Sudlow et al[6]. SNP genotypes were generated from the Affymetrix Axiom UK Biobank array and the UK BiLEVE array and underwent extensive central quality control (http://biobank.ctsu.ox.ac.uk). We based our analysis on 451,099 individuals of European descent as defined by principal-component analysis (PCA)[7]. We removed 111 participants who withdrew from the study and 348 individuals whose self-reported sex did not match their genetic sex on the basis of relative intensities of X and Y chromosome SNP probe intensity. Genotype-phenotype associations were generated using BOLT-LMM[8] which uses an LD score regression approach to

account for structure caused by relatedness adjusting for SNP chip type, age, sex and test center. For continuous traits, we inverse normalized phenotypes to account for skewed distributions. The UK Biobank has approval from the North West Multicenter Research Ethics Committee (https://www.ukbiobank.ac.uk/ethics/), and these ethics regulations cover the work in this study. Written informed consent was obtained from all participants.

**Generation of the EPO phenotype**

To generate the EPO phenotype, we included all individuals with valid genomic data, hemoglobin level data and EPO level data from 4 independent cohorts (InCHIANTI, PREVEND, BLSA, HealthABC). We excluded anemic patients as per the WHO definition (Males: hemoglobin (Hgb) levels<13g/dL, Females: Hgb<12g/dL) and patients with renal dysfunction based on an estimated glomerular filtration rate (eGFR) threshold of 50mL/min/1.73m$^2$ resulting in a final sample size of 6,127 individuals of European and African American descent (InCHIANTI: N = 1,210, PREVEND: N = 2,954, BLSA: N = 458 and HealthABC: N = 1,505). The standard cut-off for renal dysfunction is 60mL/min/1.73m$^2$ but as the study cohorts were on average older than the general population (Supplementary Table 10), a lower threshold was used due to lower eGFR rates not being unusual in older populations[9] and values between 50-60mL/min/1.73m$^2$ remaining within the normal distribution of each cohort (Supplementary Fig. 2). The eGFR was calculated using the Modification of Diet in Renal Disease (MDRD) equation[10]. We regressed EPO measures on sex and age and performed rank inverse normalization on the resulting residuals to account for skewed data.

**Imputation and Phasing**

We included chromosomes 1-23 and genotype data were reported using NCBI b37 (hg19) coordinates. For Europeans, imputation was carried out to the Haplotype Reference Consortium (HRC) version 1.1 using MiniMac3 (http://genome.sph.umich.edu/wiki/Minimac), whilst for African Americans, imputation was carried out to CAAPA [11]. Phasing was carried out using Eagle version 2.3[11].

**Quality control filters post-GWAS**

After performing GWAS, quality controls checks were undertaken and any single nucleotide polymorphisms (SNPs) with allele frequencies >4 standard deviations (SDs) or <-4 SDs from the Haplotype Reference Consortium (HRC) allele frequency were excluded[12].

**Hepatic eQTL data-set**

The three hepatic eQTL datasets, comprising a total of 861 liver samples from individuals of European ancestry were analyzed in a meta-analysis (methods and results have been reported in Etheridge et al.[13]. Tissue procurement, genotyping, and gene expression and eQTL analyses have been described previously for each of the three studies[14–16]. Genotypes were imputed to the 1000 Genomes Project Phase 1 reference panel with Minimac (http://genome.sph.umich.edu/wiki/Minimac) and expression probe sequences were mapped to ENSEMBL genes. Within each dataset, a genome-wide eQTL analysis was run with an additive genetic model including dataset specific covariates to examine *cis*-associations within a 1mb flanking window. Results from the three datasets were combined with a modified meta test-statistic which was calculated using the following approach: $t_{meta}=(\sum w_i t_i)/\sqrt{(\sum w_i^2)}$, $w=\sqrt{(n-(\#covariates)-1)}$ where i=datasets 1-3 and n=sample size. Generation of *P*-values was accomplished by assuming the meta test-statistics were normally distributed.

**Renal eQTL data-set**

The TransplantLines eQTL cohort used for the kidney analysis is part of a donor cohort for which gene expression results have been described previously[17]. The dataset includes kidneys from living donors, donated after brain death and donated after cardiac death (non-heart-beating). Time of biopsy (that is, before transplantation, before reperfusion and after reperfusion) was recorded as well. For some donors, multiple biopsies from different time points were taken. In addition, for some donors biopsies from both kidneys were available. Samples were genotyped on the Illumina CytoSNP 12 v2 array and imputed using the 1000Genomes Phase 1

ALL reference panel[18] using Impute2[19,20]. Expression and genotype data were available for 236 kidney biopsies of 134 donors. A mixed model eQTL analysis adjusting for sex, age, donor type, time of biopsy, first three principle components and sample ID, was run to account for multiple samples from a donor.

## Construction of the CRISPR-Cas9 expression vectors

Guide RNA (gRNA) sequences were identified using the online CRISPR design tools (available at https://www.benchling.com/crispr/ ) by screening the exonic regions conserved across *EPO* transcripts. This online tool uses the latest algorithms to assess the efficiency of the gRNA. Sequences with high predicted off-target scores (>60) and high predicted on-target scores (> 50) were chosen. Overhangs for cloning into a BbsI restriction cut-site were placed on the ends of the gRNAs to enable successful cloning into the CRISPR-Cas9 vector. The gRNAs were ordered from Integrated DNA technologies (IDT) (https://eu.idtdna.com/). The gRNAs were ligated into the CRISPR plasmids in a single digestion and ligation reaction using T4 ligase (New England BioLabs, Ipswich, UK) following manufacturer's protocol. We transformed bacteria (*Escherichia Coli* DH5-alpha) with ligated plasmids before purifying the plasmid DNA following the QIAprep Spin Miniprep Kit protocol (Qiagen, Maryland, USA). A double diagnostic restriction digest with BbsI and EcoRI was used to confirm successful ligation of the gRNA into the plasmid (Extended Data Fig. 2B). Plasmids with the correct digestion pattern were sent for Sanger sequencing with the LKO.1 forward primer (5'-GACTATCATATGCTTACCGT-3') to confirm insertion of the gRNA in the correct orientation and location (Extended Data Fig. 2C).

## Whole transcriptomic Analysis

Library preparation was performed using the TruSeq DNA HT Library Preparation Kit using the 3' poly-A tail primer Oligo(dT) from Illumina (Illumina, California, USA). RNA Sequencing was performed using the Illumina HiSeq 2500 high-throughput sequencing system (Illumina, California, USA). We resulted in 75 bp paired-end sequences.

## Construction of piggyBac[TM] expression vector

We designed 500 bp homology arms complementary to the genomic DNA either side of the TTAA site closest (88 bp) to the desired SNP edit (rs1617640 at position 7:100317298). In order to ensure seamless excision of the *piggyBac*™ transposon from the genomic DNA after transposase treatment, we inserted the homology arms into the *piggyBac*™ multivector (SGK:005, MV-PGK-Puro-TK) (Hera BioLabs, Kentucky, USA) at the BsiW1 (for insertion of 5’ homology arm) and Nsi1 (for insertion of 3’ homology arm) restriction sites using the Gibson Assembly Cloning Kit (New England BioLabs, Ipswich, UK). We therefore had to add the remainder of the 3’ITR region to the 5’ homology arm and the remainder of the 5’ITR region to the 3’ homology arm. For Gibson cloning to work, 20 bp of sequence complementary to the *piggyBac*™ vector either side of the cut site was also added to the either end of the homology arm sequence. Final homology sequences were ordered as MiniGenes (Integrated DNA Technologies) and then amplified out of the holding vector via PCR. Ordered homology sequences can be seen below with red depicting the desired SNP change at rs1617640, blue depicting the TTAA site, bold depicting the remainder of the 3’ITR region (for the 5’homology arms) or the 5’ITR region (for the 3’ homology arm) and the underlined bases depicting the 20 bp sequences complementary to the *piggyBac* multivector sequence (SGK:005, MV-PGK-Puro-TK).

5’ homology arm sequence for wild-type (WT) sequence at rs1617640 (A/A)

ATAATCATATTGTGACGTACTATTTATTTATTTATTTTAGAGACAAGGTCTTGCCATGTTGT<br>CCGGGCTGGTCTCGAACTCCTGGGCTCAAAGGATCTTCCTGCCTTGGTCTCCCAAAGTGCT<br>GGGATTATAGGTGTCAGCTGCGGCGCCTGGACCTTTCCTGTCTTTTATGAAACCTGAATGG<br>GATAGGCTGGTAGTTTCACCACACCCATTTGACAGATGAGGACATTGAGGGCTCAAGGAC<br>GAGGCCACTTTCTAAGGTGTGAGAGACCAGCTAGTCTTGGTCTCCTGCTCTGGGAATCTCA<br>CTCATCTGGCTCAGGGTTTCCAGAAGCCATAAAACCTTAGCTGTAAATCCCAGCCCCCAT<br>CACTCTTGGTGTTAGCTGTATTTCAGTGTTCTTAA**CCCTAGAAAGATAATCATATTGTG**<br>**AC**GTACGTTAAAGATAATCATG

5’ homology arm sequence for SNP change at rs1617640 (C/C)

ATAATCATATTGTGACGTACTATTTATTTATTTATTTTAGAGACAAGGTCTTGCCATGTTGT<br>CCGGGCTGGTCTCGAACTCCTGGGCTCAAAGGATCTTCCTGCCTTGGTCTCCCAAAGTGCT<br>GGGATTATAGGTGTCAGCTGCGGCGCCTGGACCTTTCCTGTCTTTTATGAAACCTGAATGG<br>GATAGGCTGGTAGTTTCACCACACCCATTTGACAGATGAGGACATTGAGGGCTCAAGGAC<br>GAGGCCACTTTCTAAGGTGTGAGAGACCAGCTAGTCTTGGTCTCCTGCTCTGGGAATCTCA<br>CTCCTCTGGCTCAGGGTTTCCAGAAGCCATAAAACCTTAGCTGTAAATCCCAGCCCCCAT<br>CACTCTTGGTGTTAGCTGTATTTCAGTGTTC**TTAACCCTAGAAAGATAATCATATTGT**<br>**GAC**GTACGTTAAAGATAATCATG

3’ homology arm sequence

AGCAATATTTCAAGAATGCA**TGCGTCAATTTTACGCAGACTATCTTTCTAGGG**TTAA<br>GAACTCAGCAATGCAGCCTAGCTAACCTACACCACAGGTCAAATAAACAGATGTCAAGGT<br>GCATGTGTGTCCTGCACAATGGACTGTGTGCTCTGTGCACTAAAAGTTAAGTGTCTGGGGT<br>GGGGGCTGGGTGCGGTGGCTCACGCCTGCAATCCCAGCACTTTGAGAGGCCGAGGAGGG<br>TGGATCACCTGAGGTCAGGAGTTCAAGACCGGTATGGGCAATATGGCAACACCCCATCTC<br>TACTAAAAATACAAAACATAGCCGGGTGTAAAGGTGTGCCTGTAGTCCCAGCTGCTCCAG<br>AGGCTGAAGCAGGAGAATTGCTTGAACCCAGGAGGCAGAGGTTGTAGTGAACGGGAGAT<br>GGCACCACTGCACTGCCTGGGCAACATAGGGAGGCTACATCTCCAGAAAAAAAAAAAAA<br>AGTTATGCATGCGTCAATTTTACGC

Gibson cloning was first performed for insertion of the 5’ homology arm into the *piggyBac*$^{TM}$ plasmid after digestion with the BsiW1 restriction enzyme. Ligated plasmids were transformed into bacteria (*Escherichia Coli* DH5-alpha). Plasmids were purified from overnight cultures using the QIAprep Spin Miniprep Kit protocol (Qiagen, Maryland, USA). We confirmed successful insertion of the 5’ homology arm

using PCR (Extended Data Figs. 5A-B). Successful plasmids containing the 5' homology arm was digested with Nsi1 and Gibson Cloning was repeated to insert the 3' homology arm into the *piggyBac*$^{TM}$ plasmid following manufacturer's protocol (New England BioLabs, Ipswich, UK). We transformed the ligated plasmids into competent bacteria and purified plasmid DNA from overnight cultures using the QIAprep Spin Miniprep Kit protocol (Qiagen, Maryland, USA) We confirmed successful cloning of both arms into the plasmid using a single diagnostic digest (Extended Data Fig. 5C). Sanger sequencing was then performed on final constructs to confirm correct insertion of both homology arms into the *piggyBac*$^{TM}$ plasmid (Extended Data Figs. 5D-F).

## Supplemental References


1. Ferrucci, L. *et al.* Subsystems contributing to the decline in ability to walk: bridging the gap between epidemiology and geriatric practice in the InCHIANTI study. *J. Am. Geriatr. Soc.* **48**, 1618–1625 (2000).
2. Shock, N. W. Normal human aging: The Baltimore longitudinal study of aging. (1984).
3. Pinto-Sietsma, S. *et al.* Urinary Albumin Excretion Is Associated with Renal Functional Abnormalities in a Nondiabetic Population. *J. Am. Soc. Nephrol.* **11**, 1882 LP – 1888 (2000).
4. Simonsick, E. M. *et al.* Measuring Higher Level Physical Function in Well-Functioning Older Adults: Expanding Familiar Approaches in the Health ABC Study. *Journals Gerontol. Ser. A* **56**, M644–M649 (2001).
5. Collins, R. What makes UK Biobank special? *Lancet (London, England)* **379**, 1173–1174 (2012).
6. Sudlow, C. *et al.* UK Biobank: An Open Access Resource for Identifying the Causes of a Wide Range of Complex Diseases of Middle and Old Age. *PLOS Med.* **12**, e1001779 (2015).
7. Frayling, T. M. *et al.* A Common Allele in FGF21 Associated with Sugar Intake Is Associated with Body Shape, Lower Total Body-Fat Percentage, and Higher Blood Pressure. *Cell Rep.* **23**, 327–336 (2018).
8. Loh, P.-R. *et al.* Efficient Bayesian mixed-model analysis increases association power in large cohorts. *Nat. Genet.* **47**, 284–290 (2015).
9. Wetzels, J. F. M., Kiemeney, L. A. L. M., Swinkels, D. W., Willems, H. L. & Heijer, M. de. Age- and gender-specific reference values of estimated GFR in Caucasians: The Nijmegen Biomedical Study. *Kidney Int.* **72**, 632–637 (2007).
10. Levey, A. S. *et al.* Expressing the Modification of Diet in Renal Disease Study equation for estimating glomerular filtration rate with standardized serum creatinine values. *Clin. Chem.* **53**, 766–772 (2007).
11. Das, S. *et al.* Next-generation genotype imputation service and methods. *Nat. Genet.* **48**, 1284–1287 (2016).
12. McCarthy, S. *et al.* A reference panel of 64,976 haplotypes for genotype imputation. *Nat. Genet.* **48**, 1279–1283 (2016).
13. Etheridge, A. S. *et al.* A New Liver Expression Quantitative Trait Locus Map From 1,183 Individuals Provides Evidence for Novel Expression Quantitative Trait Loci of Drug Response, Metabolic, and Sex-Biased Phenotypes. *Clin. Pharmacol. Ther.* **107**, 1383–1393 (2020).

14. Innocenti, F. *et al.* Identification, replication, and functional fine-mapping of expression quantitative trait loci in primary human liver tissue. *PLoS Genet.* **7**, e1002078 (2011).

15. Schadt, E. E. *et al.* Mapping the genetic architecture of gene expression in human liver. *PLoS Biol.* **6**, e107 (2008).

16. Greenawalt, D. M. *et al.* A survey of the genetics of stomach, liver, and adipose gene expression from a morbidly obese cohort. *Genome Res.* **21**, 1008–1016 (2011).

17. Damman, J. *et al.* Hypoxia and complement-and-coagulation pathways in the deceased organ donor as the major target for intervention to improve renal allograft outcome. *Transplantation* **99**, 1293–1300 (2015).

18. Auton, A. *et al.* A global reference for human genetic variation. *Nature* **526**, 68–74 (2015).

19. Howie, B. N., Donnelly, P. & Marchini, J. A Flexible and Accurate Genotype Imputation Method for the Next Generation of Genome-Wide Association Studies. *PLOS Genet.* **5**, e1000529 (2009).

20. Howie, B., Marchini, J. & Stephens, M. Genotype Imputation with Thousands of Genomes. *G3 Genes, Genomes, Genet.* **1**, 457–470 (2011).
