# Supplementary material for: Identification and single-base gene-editing functional validation of a cis-EPO variant as a genetic predictor for EPO-increasing therapies
Source: Am J Hum Genet. 2022 Sep 1;109(9):1638–52. doi: 10.1016/j.ajhg.2022.08.004 (PMC9502050; doi:10.1016/j.ajhg.2022.08.004)
Supplement: Document S2. Article plus supplemental information [file mmc4.pdf]

Identification and single-base gene-editing functional validation of a *cis-EPO* variant as a genetic predictor for EPO-increasing therapies

Graphical abstract

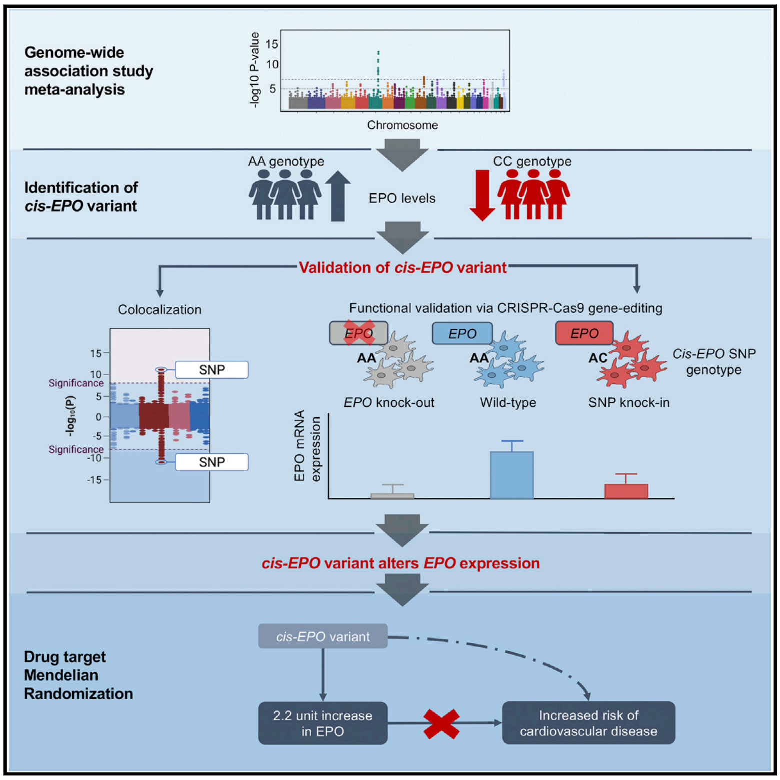

Authors

Charli E. Harlow, Josan Gandawijaya, Rosemary A. Bamford, ..., Audrey Y. Chu, Asami Oguro-Ando, Timothy M. Frayling

Correspondence

a.oguro-ando@exeter.ac.uk (A.O.-A.), t.m.frayling@exeter.ac.uk (T.M.F.)

We identified a common genetic variant associated with EPO levels by performing a GWAS. We successfully validated the genetic variant as causal by using gene-editing techniques. Finally, we used the variant as a partial predictor for EPO-raising therapies and found no evidence of adverse cardiovascular events with long-term EPO rises.

Harlow et al., 2022, The American Journal of Human Genetics *109*, 1638–1652
September 1, 2022 © 2022 The Author(s).
https://doi.org/10.1016/j.ajhg.2022.08.004

ARTICLE

Identification and single-base gene-editing functional validation of a *cis-EPO* variant as a genetic predictor for EPO-increasing therapies

Charli E. Harlow,[1] Josan Gandawijaya,[1] Rosemary A. Bamford,[1] Emily-Rose Martin,[1] Andrew R. Wood,[1] Peter J. van der Most,[2] Toshiko Tanaka,[3] Hampton L. Leonard,[4,5,6] Amy S. Etheridge,[7] Federico Innocenti,[8] Robin N. Beaumont,[1] Jessica Tyrrell,[1] Mike A. Nalls,[4,5,6] Eleanor M. Simonsick,[3] Pranav S. Garimella,[9] Eric J. Shiroma,[10] Niek Verweij,[11] Peter van der Meer,[11] Ron T. Gansevoort,[12] Harold Snieder,[2] Paul J. Gallins,[13] Dereje D. Jima,[13,14] Fred Wright,[13] Yi-hui Zhou,[13] Luigi Ferrucci,[3] Stefania Bandinelli,[15] Dena G. Hernandez,[4] Pim van der Harst,[16] Vickas V. Patel,[17,19] Dawn M. Waterworth,[17,20] Audrey Y. Chu,[18] Asami Oguro-Ando,[1,*] and Timothy M. Frayling[1,*]

Summary

Hypoxia-inducible factor prolyl hydroxylase inhibitors (HIF-PHIs) are currently under clinical development for treating anemia in chronic kidney disease (CKD), but it is important to monitor their cardiovascular safety. Genetic variants can be used as predictors to help inform the potential risk of adverse effects associated with drug treatments. We therefore aimed to use human genetics to help assess the risk of adverse cardiovascular events associated with therapeutically altered EPO levels to help inform clinical trials studying the safety of HIF-PHIs. By performing a genome-wide association meta-analysis of EPO (n = 6,127), we identified a *cis-EPO* variant (rs1617640) lying in the *EPO* promoter region. We validated this variant as most likely causal in controlling EPO levels by using genetic and functional approaches, including single-base gene editing. Using this variant as a partial predictor for therapeutic modulation of EPO and large genome-wide association data in Mendelian randomization tests, we found no evidence (at $p < 0.05$) that genetically predicted long-term rises in endogenous EPO, equivalent to a 2.2-unit increase, increased risk of coronary artery disease (CAD, OR [95% CI] = 1.01 [0.93, 1.07]), myocardial infarction (MI, OR [95% CI] = 0.99 [0.87, 1.15]), or stroke (OR [95% CI] = 0.97 [0.87, 1.07]). We could exclude increased odds of 1.15 for cardiovascular disease for a 2.2-unit EPO increase. A combination of genetic and functional studies provides a powerful approach to investigate the potential therapeutic profile of EPO-increasing therapies for treating anemia in CKD.

Introduction

Anemia, one of the primary complications of chronic kidney disease (CKD), affects one out of every seven individuals with CKD.[1–3] Anemia is associated with faster progression of CKD and increased risk of adverse events, particularly heart disease or stroke, two of the major causes of death associated with CKD.[4,5] Current therapies used to treat anemia in CKD include blood transfusions, intravenous iron therapies, or parenteral injections of recombinant erythropoietin (rhEPO). These treatments have safety and compliance concerns, including risk of infection, adverse gastro-intestinal effects, and increased risk of stroke, myocardial infarction (MI), venous thromboembolism, and heart failure.[4,6–12] These safety concerns have led to the development of hypoxia-inducible factor (HIF) prolyl hydroxylase inhibitors (PHIs) as a novel class of treatment for anemia in CKD. PHIs inhibit the prolyl hydroxylase enzymes (PHD1-3) allowing the level of HIF-1 to rise and bind to the hypoxia response element that, among other activities, increases the transcription of *EPO* and in turn endogenous EPO levels.[13,14] EPO stimulates the bone marrow to increase the production of red blood cells and circulating hemoglobin (Hgb) levels.[12] The recent completion of phase III clinical

[1]University of Exeter Medical School, University of Exeter, Royal Devon and Exeter NHS Trust, Exeter EX2 5DW, UK; [2]University of Groningen, University Medical Center Groningen, Department of Epidemiology, Groningen 9713, the Netherlands; [3]Longitudinal Studies Section, Translation Gerontology Branch, National Institute on Aging, Baltimore, MD 21224, USA; [4]Laboratory of Neurogenetics, National Institute on Aging, NIH, Bethesda, MD 20892, USA; [5]Data Tecnica International, Glen Echo, MD 20812, USA; [6]Center for Alzheimer's and Related Dementias, National Institutes of Health, Bethesda, MD 20892, USA; [7]Eshelman School of Pharmacy and Center for Pharmacogenomics and Individualized Therapy, University of North Carolina at Chapel Hill, 120 Mason Farm Road, Chapel Hill, NC 27599, USA; [8]AbbVie Inc., 1000 Gateway Boulevard, South San Francisco, CA 94080, USA; [9]Division of Nephrology-Hypertension, University of California San Diego, San Diego, CA, USA; [10]Laboratory of Epidemiology and Population Sciences, National Institute on Aging, Bethesda, MD 20892, USA; [11]University of Groningen, University Medical Center Groningen, Department of Cardiology, Groningen 9713, the Netherlands; [12]University of Groningen, University Medical Center Groningen, Department of Nephrology, Groningen 9713, the Netherlands; [13]Bioinformatics Research Center, North Carolina State University, 1 Lampe Drive, Raleigh, NC 27695, USA; [14]Center for Human Health and the Environment, North Carolina State University, Raleigh, NC 27606, USA; [15]Geriatric Unit, Azienda Sanitaria Firenze, Florence 50134, Italy; [16]Department of Cardiology, University Medical Center Utrecht, Utrecht 3584, the Netherlands; [17]GlaxoSmithKline, Collegeville, PA 19426, USA; [18]GlaxoSmithKline, Boston, MA 02140, USA
[19]Present address: Spark Therapeutics, Inc., Philadelphia, PA 19104, USA
[20]Present address: Immunology Translational Sciences, Janssen, Spring House, PA 19044, USA
*Correspondence: a.oguro-ando@exeter.ac.uk (A.O.-A.), t.m.frayling@exeter.ac.uk (T.M.F.)
https://doi.org/10.1016/j.ajhg.2022.08.004.
© 2022 The Author(s). This is an open access article under the CC BY license (http://creativecommons.org/licenses/by/4.0/).

trials assessing cardiovascular safety has indicated non-inferiority of PHIs compared to rhEPO and shown that PHIs can increase and maintain Hgb levels with small increases in circulating EPO levels compared to exogenous EPO administration.[4,15–23] PHIs have already received approval for clinical use in Japan, supporting ongoing development elsewhere.[11,15,18,24–26]

To further assess the potential side-effects of EPO-based treatments, we used a genetic approach. Several studies have shown that genetic data can provide supporting evidence of an association between the drug target and intended therapeutic indication. Genetic associations can also help identify potential unintended effects and inform potential drug safety profiles.[27–33] Genetic variants lying within or nearby the gene encoding the drug target are more likely to have functional impact on the protein product than those further away in the genome.[34] These variants can be used in Mendelian randomization (MR) tests as partial proxies for pharmacological action to help inform the effects of long-term modulation of drug targets on disease outcomes.[35–37] The properties of inherited genetic variation mean the associations are far less likely to be confounded or biased, compared to observational studies. Functional studies can provide further evidence that a genetic variant is a valid proxy for a drug target, for example, by confirming that the variant controls the expression of the expected gene. The development of CRISPR-Cas9 gene editing has made functional validation more straightforward by establishing whole-gene knockouts and modifying single-nucleotide polymorphisms (SNPs).[38–40]

This study aimed to use human genetics to examine the long-term effect of genetically predicted therapeutic modulation of endogenous EPO levels. First, we identified a genetic variant lying *in cis* with *EPO* that is associated with endogenous circulating EPO levels. Second, we functionally validated that variant by using CRISPR-Cas9 gene-editing techniques. Third, we used this variant in drug-target MR tests as a genetic predictor for therapeutically altered EPO levels to help inform the long-term effects of elevated endogenous EPO levels on risk of cardiovascular disease (CVD) or clinical markers (blood pressure and resting heart rate) predisposing to CVD risk factors (e.g., hypertension).

Material and methods

The steps performed to identify and functionally validate a genetic variant associated with circulating EPO are outlined in Figure S1 and, briefly, are as follows.

Identification of genetic variants associated with circulating EPO levels

To identify genetic variants associated with circulating EPO levels, we performed a genome-wide association study (GWAS) meta-analysis of 6,127 individuals of European and African descent from four independent cohorts: InCHIANTI (n = 1,210), PREVEND (n = 2,954), BLSA (n = 458), and HealthABC (n = 1,505) (Table S1). Details of phenotype generation and inclusion criteria are described in the supplemental material and methods, and an analysis plan is outlined in Figure S2. Each study was approved by an institutional review board as described in the supplemental material and methods. We inverse normalized EPO levels to account for a skewed distribution and performed GWAS in GEMMA[41] by using an additive linear mixed model adjusting for age, sex, and any study-specific covariates alongside a genomic relationship matrix to account for all types of relatedness. We combined study-specific estimates and performed inverse variance-weighted fixed-effects meta-analysis on ~25.1 million imputed SNPs in 6,127 unrelated individuals of European and African descent by using METAL[42] with the following filters: minor allele count (MAC) > 3, effect allele frequency (EAF) > 1, EAF < 0, Info ≥ 0.3. After performing meta-analysis, we excluded SNPs with a minor allele frequency (MAF) < 0.01 and performed a multi-SNP-based step-wise conditional and joint association analysis by using GCTA-COJO[43,44] to select SNPs independently associated with EPO ($p < 5 \times 10^{-8}$).

Identification and validation of *cis-EPO* genetic variant

To identify a genetic variant most likely to directly impact on the protein product for use as a genetic predictor for therapeutically altered endogenous EPO levels, we analyzed the GWAS data around *EPO* specifically to identify any *cis*-acting genetic variants and selected variants previously identified. We converted the genetic effect estimate (in SDs) of the genetic variant to original units (IU/L) by using the standard deviation from the InCHIANTI study (Table S2).

Expression quantitative trait loci (eQTLs) analysis

Having identified a *cis-EPO* variant (rs1617640) associated ($p = 9.32 \times 10^{-4}$) with circulating EPO protein levels, we tested its *cis*-effects (+/− 500 kb) with gene expression in a meta-analysis of hepatic gene expression from 861 livers from European individuals in three datasets[45] (Table S3) and 236 kidneys from 134 individuals in one renal gene expression dataset[46] (Table S4). Additional study details can be found in supplemental material and methods. We selected the liver and kidney, as *EPO* is highly expressed in both tissues.

Colocalization analysis

We performed colocalization analysis to assess the likelihood that the liver *EPO* eQTL was the same signal as the circulating protein level association. We obtained summary data for hepatic *cis*-eQTLs associated with *EPO* expression (false discovery rate [FDR] < 0.1) 500 kb on either side of rs1617640 and extracted the summary statistics for these SNPs from our circulating EPO meta-analysis. We performed approximate Bayes factor colocalization analyses by using the R coloc package.[47,48] We obtained overall estimates of the posterior probability that both our EPO meta-analysis and the liver eQTL share the same causal variant.

Establishment of whole-*EPO* knockout

With the use of CRISPR-Cas9 gene-editing technology, human embryonic kidney (HEK)-293 cells were subjected to gene editing to generate isogenic *EPO* knockout (*EPO*$^{-/-}$) cell lines. In brief, paired guide-RNAs (gRNAs), one targeting exon 2 (Ensembl: ENST00000252723.3) (5′-AGAGGTACCTCTCCAGGACT**CGG**-3′) and one targeting exon 4 (5′-CATGTGGATAAAGCCGTCAG**TGG**-3′), were separately cloned into the CRISPR-Cas9 expression vector containing a green fluorescent protein (GFP) reporter

(pSpCas9(BB)-2A-GFP (PX458), Addgene: #48138) and an mCherry fluorescent protein reporter (pU6-(BbsI) CBh-Cas9-T2A-mCherry, Addgene: #64324), respectively. 1×10^6 HEK-293 cells were seeded in 10 cm plates and 24 h later were co-transfected with 6 μg of each CRISPR-Cas9/gRNA vector using Lipofectamine LTX reagent (Thermo Fisher Scientific, Massachusetts, USA). Successfully transfected GFP and red fluorescent protein-positive cells were manually isolated under the EVOS FLoid Imaging system (Thermo Fisher Scientific, Massachusetts, USA). Single cells were clonally expanded for around 2 weeks and media containing 10% FBS was replaced every 48 h. Single cells were screened via polymerase chain reaction (PCR) assay using primers on either side of the two gRNAs (forward: 5′-TCTAGAATGTCCTGCCTGGC-3′, reverse: 5′-GGCCCTGTGACA TCCTTAGA-3′). Sanger sequencing was used to confirm successful disruption to *EPO*. All wild-type (WT) HEK-293 cells were treated the same throughout the experiments but treated with empty CRISPR-Cas9 plasmids (i.e., containing no gRNAs).

EPO overexpression

For use as a positive control, wild-type HEK-293 cells were transfected with an *EPO* overexpression (hEPO) construct (Addgene: #139057)[49] using Lipofectamine LTX reagent (Thermo Fisher Scientific, Massachusetts, USA).

RNA extraction and qRT-PCR

Total RNA was isolated and purified from $EPO^{-/-}$ and WT HEK-293 cell lines using the Direct-zol RNA Miniprep kit following manufacturers protocol (Cambridge Biosciences, Cambridge, UK). 500 ng of RNA was converted to cDNA using PrimeScript RT reagent kit (Takara Bio Europe SAS, Saint-Germain-en-Laye, France). Quantitative reverse transcription (qRT)-PCR was performed using Hot FIREPol EvaGreen qPCR Master Mix with ROX (Solis BioDyne, Teaduspargi, Estonia) with the QuantStudio 6 Flex qPCR machine (Thermo Fisher Scientific, Massachusetts, USA) on at least three biological replicates. Primer sequences are listed in Table S5. Samples with Ct values > 2 SD from the mean were removed. Gene expression levels were standardized against the reference gene *GAPDH* messenger RNA (mRNA) levels using the $2^{-\Delta\Delta Ct}$ method.[50] Expression of alternative housekeeping genes (*UBC* and *Pol2ra*) was also assessed. RefFinder[51] was used to determine the most stable gene or combination of genes for use as an endogenous control (Table S6). Differences in gene expression levels between WT and $EPO^{-/-}$ cell lines were investigated for statistical significance by a paired t test carried out in RStudio version 3.6.1.[52]

RNA sequencing (RNA-seq)

After RNA extraction, RNA concentration and quality were assessed using the Qubit 4 Fluorometer (Thermo Fisher Scientific, Massachusetts, USA) and the Agilent 2200 TapeStation System, respectively (Agilent Technologies, California, USA). 1,000 ng of RNA from samples with an RNA integrity (RIN) score > 8 were prepared for RNA-seq.[53] Library preparation and sequencing were performed by the Exeter Sequencing Service, resulting in 75 bp paired-end sequencing (additional details in supplemental material and methods). Quality control checks were undertaken on the raw reads using MultiQC.[54] Adapter sequences (defined by Illumina), nucleotides with poor quality from the 3′ end (Phred < 25), and reads shorter than 25 bp were removed using CutAdapt version 1.13[55] (Figure S3). Reads were aligned to the *Homo sapiens* GRCh38/hg38 reference genome using STAR version 2.7.1[56] (Figure S4) and gene quantification was performed using the *featureCounts* subread package[57] based on Ensembl GRCh38/hg38 annotation release version 2.0.0 (Figure S5). All other analysis was performed in RStudio unless otherwise stated.[52] Transcripts whose mean count across all samples were <10 were removed. Counts were normalized and transformed using the "*rlogTransformation*" function implemented in *DeSeq2*.[58] Principal-component analysis (PCA) was performed using the R "*prcomp*" function to check for similarity between samples. To identify genotype-specific gene expression changes, we performed differential gene expression analysis by using *DeSeq2*.[58] p values were calculated using the Wald test, and a Benjamini-Hochberg correction was applied to account for multiple testing. Statistically significant differentially expressed genes (DEGs) were determined by an adjusted p value ≤ 0.05. We determined strong differential expression when genes were regulated by 2-fold. As we performed RNA-seq on two $EPO^{-/-}$ cell lines (KOA and KOB) to obtain the most accurate list of DEGs most likely to be differentially expressed due to the effect of $EPO^{-/-}$, differential expression analysis was performed comparing WT to each knockout cell line (WT versus KOA and WT versus KOB). DEGs (p ≤ 0.05) overlapping in both comparisons were identified using the R "*venn.diagram*" function. Gene set enrichment analysis was performed on this list of overlapping DEGs using Enrichr.[59–61] Top DEGs were subsequently subjected to qRT-PCR to validate differential expression.

Immunoblot analysis

$EPO^{-/-}$, WT controls, and HEK-293 cells transfected with hEPO overexpression vector[49] were subjected to immunoblot analysis, as described previously,[62] using a monoclonal mouse anti-EPO antibody (1:1,000; MAB2871, R&D systems, Abingdon, UK)[63] and a mouse anti-GAPDH antibody (1:1,000; sc-47724, Santa Cruz Biotechnology, Texas, USA). Goat anti-mouse IgG (H + L) Cross-Absorbed Alexa Fluor 680 (1:5,000; A21057, Invitrogen, Massachusetts, USA), was used as the secondary antibody. Membranes were visualized on the LI-COR Odyssey CLx system (LI-COR Biotechnologies, Nebraska, USA). Images were converted to grayscale using Image Studio Lite version 5.2.5 (LI-COR Biotechnologies, Nebraska, USA).

Knockin of the rs1617640 C allele via CRISPR-Cas9 gene editing with the *piggyBac* transposon system

Using CRISPR-Cas9 and the *piggyBac* system, HEK-293 cells were subjected to single-base gene editing to generate isogenic cell-lines with either the A/A or A/C genotype for rs16167640 (the A allele being associated with higher circulating EPO levels) using a previously described protocol.[64] In brief, a single gRNA (5′-GGAATCT-CACTCCTCTGGCTCAGGG-3′) was cloned into the GFP CRISPR-Cas9 expression vector. In addition, 5′ and 3′ homology arms were designed (supplemental material and methods), one containing the desired base edit, and cloned into the *piggyBac* multivector (SGK:005, MV-PGK-Puro-TK, Hera BioLabs, Kentucky, USA) either side of the *piggyBac* transposon (containing puromycin/TK selection cassettes) using the BsiW1 and Nsi1 cut-sites (supplemental material and methods). Co-transfection was performed as described above. After 48 h, cells were cultured in a 10 cm dish under puromycin (1 μg/mL) (Sigma-Aldrich, Missouri, USA)[65,66] for 14 days, replacing media every 2–3 days, to select for cells containing the *piggyBac* transposon. Following selection, single puromycin-resistant cells were isolated and clonally expanded. Cells were screened via PCR and Sanger sequencing to confirm successful insertion of the *piggyBac* transposon and successful editing of rs16167640 from the parental A/A genotype to C/C genotype

(primer sequences listed in Table S7). Isogenic HEK-293 cells containing the *piggyBac* transposon were subsequently transfected with 2.5 μg of *piggyBac* transposase in a six-well plate using lipofectamine LTX (Thermo Fisher Scientific, Massachusetts, USA) to remove the *piggyBac* selection cassette from the genome. 48 h after transfection, cells were subject to 200 nM Fialuridine (FIAU) (Sigma-Aldrich, Missouri, USA) selection for 7 days, replacing media every 2–3 days, to select for cells no longer carrying the *piggyBac* transposon cassette. Following selection, single FIAU-resistant cells were isolated and clonally expanded. Cells were screened via PCR (Table S7) and Sanger sequencing to confirm complete removal of the *piggyBac* transposon and successful editing of rs1617640. Successfully edited isogenic HEK-293 cells were subjected to qRT-PCR of the *EPO* gene to measure *EPO* gene expression levels and investigate the effect of the *cis-EPO* SNP on dysregulated Notch signaling genes identified through transcriptomic profiling of the $EPO^{-/-}$ (*HEY2*, *DTX3L*, *PARP9*).

Using the *cis-EPO* variant to examine the therapeutic profile and cardiovascular risk of genetically proxied therapeutic modulation of endogenous EPO levels

Drug-target two-sample Mendelian randomization

To investigate the association between higher endogenous EPO levels and risk of CVD, we performed a two-sample MR by using the *cis-EPO* variant as the genetic instrument. We obtained genotype-exposure association statistics from our EPO meta-analysis (n = 6,127). Primary outcomes were CAD, MI, or stroke using GWAS data consisting of 60,801, 42,561, and 40,585 cases, respectively[64,67] (Table S8). We also performed a GWAS using UKB on CAD, MI, or stroke in 37,741, 105,90, and 9,092 cases, respectively (supplemental materials and methods, Table S8). Where we had genotype-outcome association data from both UKB and publicly available GWASs, we performed an inverse variance-weighted, fixed-effects meta-analysis by using metan[68] to estimate the overall genotype-outcome association effect estimate. As only one genetic variant was used as an instrument, we calculated an overall causal estimate between the exposure and outcome by using the Wald ratio.[69] We also performed MR to investigate the effect of higher circulating EPO levels on levels of clinical markers (systolic blood pressure [SBP], diastolic blood pressure [DBP], and resting heart rate) predisposing to CVD risk factors[70,71] (supplemental materials and methods, Table S8).

Comparing clinical trial effects and genetic association to estimate the genetically predicted impact of therapeutically altered endogenous EPO levels on cardiovascular risk

To scale the genetic effect estimates to a more representative, therapeutically relevant effect, we obtained the effects of a PHI in patients on dialysis from a phase II fixed dose randomized control trial (RCT).[72] The RCT provided an estimate of the effect of a fixed dose of HIF-PHIs on EPO levels during the first 4 weeks (median "maximum" change in EPO levels from baseline at week 4 [27.1]/SD at baseline [61] = 0.44 SD).[72] The scaling factor was calculated by dividing the PHI-induced effect (0.44 SD) by the effect of the *cis-EPO* SNP on EPO levels (0.063 SD). We used this value (7.05) to scale the genetically instrumented effect estimates and 95% confidence intervals of the *cis-EPO* SNP on CVD or clinical markers for CVD risk factors to the effect of PHIs on endogenous EPO levels.

Phenome-wide association study (PheWAS)

To further investigate the therapeutic profile of modulated EPO levels, we tested the association of rs1617640 near *EPO* with 869 traits in up to 451,099 UKB individuals of European ancestry (supplemental materials and methods). Genotype-phenotype associations were generated using BOLT-LMM[73] and traits were selected as previously described in Frayling et al.[74] For continuous traits, we performed inverse normalization prior to regression analysis to account for skewed distributions.

Results

Identification of three genomic loci associated with EPO levels at genome-wide significance

To identify human genetic variants associated with circulating EPO levels and using these variants as genetic predictors of therapeutically elevated endogenous EPO levels, we performed a GWAS meta-analysis of circulating EPO. We used 6,127 individuals of European and African American descent. After conditional analysis, we identified three genomic loci containing three independent signals associated with circulating EPO ($p < 5 \times 10^{-8}$) (Table S1, Figure S6). The most strongly associated genetic variant, rs4895441 (6q23, *HBS1L-MYB* locus), had been previously identified as associated with circulating EPO levels in a GWAS of 2,691 individuals.[75] This variant has stronger primary effects on other erythrocyte phenotypes in previously published GWASs and a PheWAS in UK Biobank (UKB) European individuals (Table S9). The remaining two independent genomic loci (rs855791 and rs112631630) represent novel associations with circulating EPO levels. However, rs855791, located in the *TMPRSS6* locus, has primary, stronger effects on several other erythrocyte phenotypes compared to the effect on circulating EPO in a PheWAS in UKB European individuals (Table S9) and has been previously associated with other erythrocyte phenotypes and iron homeostasis biomarkers in GWASs.[76,77] The variant (rs112631630) located in the *NRAP* locus is only present in one study (African Americans), and we were unable to test in additional datasets. Therefore, these variants were not deemed suitable, specific instruments used in subsequent MR analysis to genetically predict therapeutic modulation of endogenous EPO levels.

Identification of *cis*-SNP lying in the promoter region of *EPO* for use as a genetic predictor for the therapeutic alteration of endogenous EPO levels

The conditionally independent lead variants identified by the GWAS meta-analysis were not sufficiently specific instruments to act as genetic predictors for higher endogenous EPO levels. We therefore looked for *cis* effects at the *EPO* locus. Previous associations have been reported between variants near *EPO* and circulating EPO levels.[78,79] A *cis*-SNP, rs1617640, lying in the *EPO* promoter region, 1,125 bp upstream of the transcription start site, was associated with EPO levels in our study and in the previous study.[78] On the basis of our meta-analysis, each copy of the A-allele at rs1617640 was associated with 0.063 standard deviations (SD), equivalent to 0.32 IU/L, higher endogenous EPO levels ($p = 9.32 \times 10^{-4}$) (Table 1). The

Table 1. Summary statistics for association between the *cis-EPO* genetic variant (rs1617640) and circulating EPO levels or hepatic *EPO* gene expression

Analysis	RSID	Chromosome	Position	A1	A2	Freq A1	β	SE	p value	Sample size
EPO meta-analysis	rs1617640	7	100,317,298	A	C	0.62	0.063	0.02	9.32E−4	6,127
**Analysis**	**RSID**	**Chromosome**	**Position**	**A1**	**A2**	**Freq A1**	**t-meta**	**SE**	**p value**	**Sample size**
Liver eQTL analysis	rs1617640	7	100,317,298	A	C	0.62	5.39	0.15	6.86E−8	861

effect of the *cis-EPO* variant is consistent with that previously reported in affected individuals with diabetic retinopathy or hepatitis C.[78,79]

The *cis-EPO* SNP is associated with altered *EPO* expression and nearby *TFR2* expression in the liver

To provide additional insight into the rs1617640-EPO association and further evaluate its utility as an instrument, we tested the association of the *cis-EPO* SNP with gene expression in the kidney and the liver as *EPO* is highly expressed in these two tissues.[80] In the liver, we found that the C allele at rs1617640 was associated with higher *EPO* expression ($\beta = 0.22$ [0.14, 0.3], $p = 6.86 \times 10^{-8}$) and also *TFR2* expression ($\beta = 0.23$ [0.17, 0.29], $p = 1.56 \times 10^{-13}$), a gene that lies upstream of the *EPO* gene and is involved in iron metabolism (Table S3).[76,81] No effect of rs1617640 on renal expression of *EPO* ($\beta = 0.16$ [−2.46, 2.78], $p > 0.05$) or *TFR2* ($\beta = 1.33$ [−0.59, 3.26], $p > 0.05$) was found (Table S4). We, therefore, proceeded with hepatic results only. Colocalization analysis provided evidence that the variant associated with circulating EPO levels in the meta-analysis and hepatic *EPO* mRNA expression has a 71% posterior probability of being the casual variant (Figure 1).

Functional validation of rs1617640 in influencing *EPO* expression

To further validate the rs1617640 variant, we first sought a better understanding of the downstream causal genes and signaling cascades of EPO. We generated two *EPO* knockout (*EPO*$^{-/-}$) cell lines by using CRISPR-Cas9 gene editing with a paired gRNA approach (Figures 2A and 2B, Figure S7). By performing RNA sequencing (RNA-seq) analysis, we found large transcriptional differences between *EPO*$^{-/-}$ and wild-type (WT) cell lines (Figures 2C and S8). To obtain a list of differentially expressed genes (DEGs) specific to *EPO* knockdown and not due to potential differences in cellular conditions, we performed differential gene expression analysis by comparing controls to the two *EPO*$^{-/-}$ cell lines, KOA and KOB, (Figures S9A and S9B) and then combined lists of DEGs to obtain a final list of 3,722 overlapping DEGs ($p \leq 0.05$) (Figures 2D and 2E, Table S10). 3,501 of the overlapping DEGs showed consistent directions of differential expression (Figure S9C, $r^2 = 0.90$, $p < 2.2 \times 10^{-16}$) and were considered for downstream analysis. 314 of the 3,501 had a $\log_2$ fold-change $\geq |2|$. We validated the differential expression of four DEGs ($p \leq 0.05$, $\log_2$ fold-change $\geq |2|$) with the highest expression in HEK-293 cells (based on the Human Cell Atlas[82]) by using qRT-PCR (Figure 2F). Gene Ontology (GO) analysis of the 3,501 DEGs suggested enrichment for multiple biological processes involved in DNA repair, mRNA processing, ATPase activity, notch signaling, apoptosis, fatty acid oxidation, and cellular respiration (Table S11). Notch signaling and related mitogenic pathways featured prominently in these GO analyses (Figure 2G), so we selected seven genes from these pathways and validated differential expression with qRT-PCR (Figure 2H).

To functionally validate the *cis-EPO* SNP as the most likely causal variant in controlling *EPO* expression levels and therefore the most valid instrument for use to genetically predict the associated risk of CVD, we used CRISPR-Cas9 and the *piggyBac* transposon system to generate two isogenic cell lines, one homozygous for the A allele (A/A) and one heterozygous for the A allele (A/C) at rs1617640 (Figures 3A–3D, S10, and S11). We performed qRT-PCR in the A/A and A/C isogenic cell lines. We showed that homozygous cells for the A allele of rs1617640 had higher *EPO* mRNA expression levels than heterozygotes for the A allele, confirming that rs1617640 has an allele-specific effect on *EPO* gene expression levels (Figure 3E). These results are consistent with our genetic findings that the A allele is associated with higher circulating EPO levels. We also performed qRT-PCR on three Notch-signaling genes (*HEY2*, *DTX3L*, *PARP9*), which showed differential expression in the *EPO*$^{-/-}$ knockouts compared to wild-type, to see whether specific alteration of the *cis-EPO* variant also resulted in dysregulated Notch signaling. We found that A allele homozygotes of rs1617640 had a down-regulated expression of these Notch-signaling genes compared to heterozygotes (Figure 3F). Negative control experiments with two genes not differentially expressed in *EPO*$^{-/-}$ confirmed that the *cis*-SNP editing was specific to the EPO pathways (Figure 3F). As the eQTL analysis showed that the *cis-EPO* SNP was associated with hepatic *TFR2* expression, we also performed qRT-PCR to further investigate the potential pleiotropic effect of the *cis*-SNP. We found no difference in *TFR2* mRNA expression in A allele heterozygotes compared to A allele homozygotes or *EPO*$^{-/-}$ knockouts (Figure S12).

Genetically predicted long-term higher endogenous EPO levels are not associated with increased cardiovascular risk

We used the *cis-EPO* SNP as an instrument in two-sample Mendelian randomization (MR) as a genetic predictor for

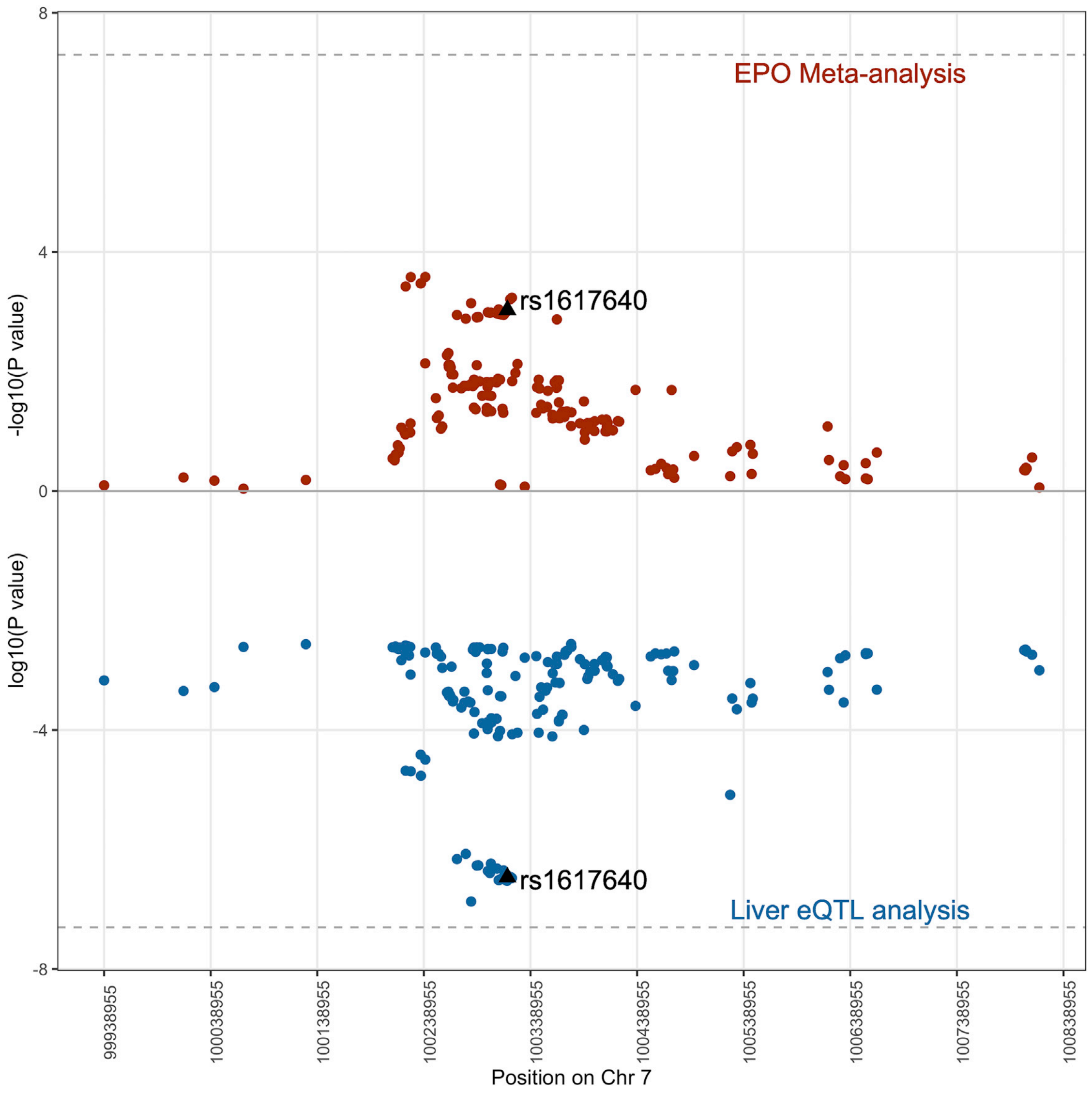

Figure 1. Colocalization analysis of circulating endogenous EPO levels and liver *EPO* gene expression shows that the same genetic variant (*cis-EPO* variant) is likely to be the causal variant (posterior probability = 71%)
We tested for colocalization within a 500 kb flanking window of the *cis-EPO* genetic variant (rs1617640—labeled and represented as a black diamond on the plot). The top half of the Miami plot (red dots) represents $-\log_{10}(p)$ for the circulating EPO levels obtained from the EPO meta-analysis whilst the bottom half of the Miami plot (blue dots) represents $\log_{10}(p)$ for *EPO* gene expression in the liver obtained from a hepatic eQTL meta-analysis. The dashed lines represent genome-wide significance levels of $p = 5 \times 10^{-8}$.

therapeutically altered EPO levels on risk of CVD or levels of clinical markers predisposing to CVD risk factors. The three main CVDs of interest were coronary artery disease (CAD; 98,542 cases, 442,396 controls), myocardial infarction (MI; 53,151 cases, 564,013 controls), and stroke (49,677 cases, 752,534 controls) because of availability of large-scale genetic association data. The clinical markers of interest were systolic blood pressure (SBP; n = 678,320), diastolic blood pressure (DBP; n = 677,567), and resting heart rate (n = 514,695). Using these very large sample sizes, we found no evidence (at $p < 0.05$) of a genetic association between 1 SD higher endogenous EPO levels (equivalent to 5.1 IU/L) and increased odds of CAD (odds ratio [OR] [95% CI] = 1.03 [0.85, 1.25], p = 0.72), stroke (OR [95% CI] = 0.92 [0.70, 1.21], p = 0.55), or MI (OR [95% CI] = 0.98 [0.75, 1.29], p = 0.89) (Table S12). We found evidence of a genetic association between 1 SD, equivalent to 5.1 IU/L, higher endogenous EPO levels, and lower resting heart rate (effect estimate [95% CI] = −0.996 [−1.74, −0.25], p = 0.01) and lower DBP (effect estimate [95% CI] = −0.98 [−1.67, −0.29], p = 0.006) but not with SBP (effect estimate [95% CI] = 0.53 [−0.65, 1.71], p = 0.38) (Table S12).

Comparison of the genetic associations with the effects observed in clinical trials

In an RCT, individuals receiving a PHI (daprodustat) had EPO levels 0.44 SD (27.1/61), equivalent to 2.2 IU/L, higher than baseline EPO levels.[72] Given that the per-allele effect of rs1617640 on endogenous EPO was 0.063 SD, we

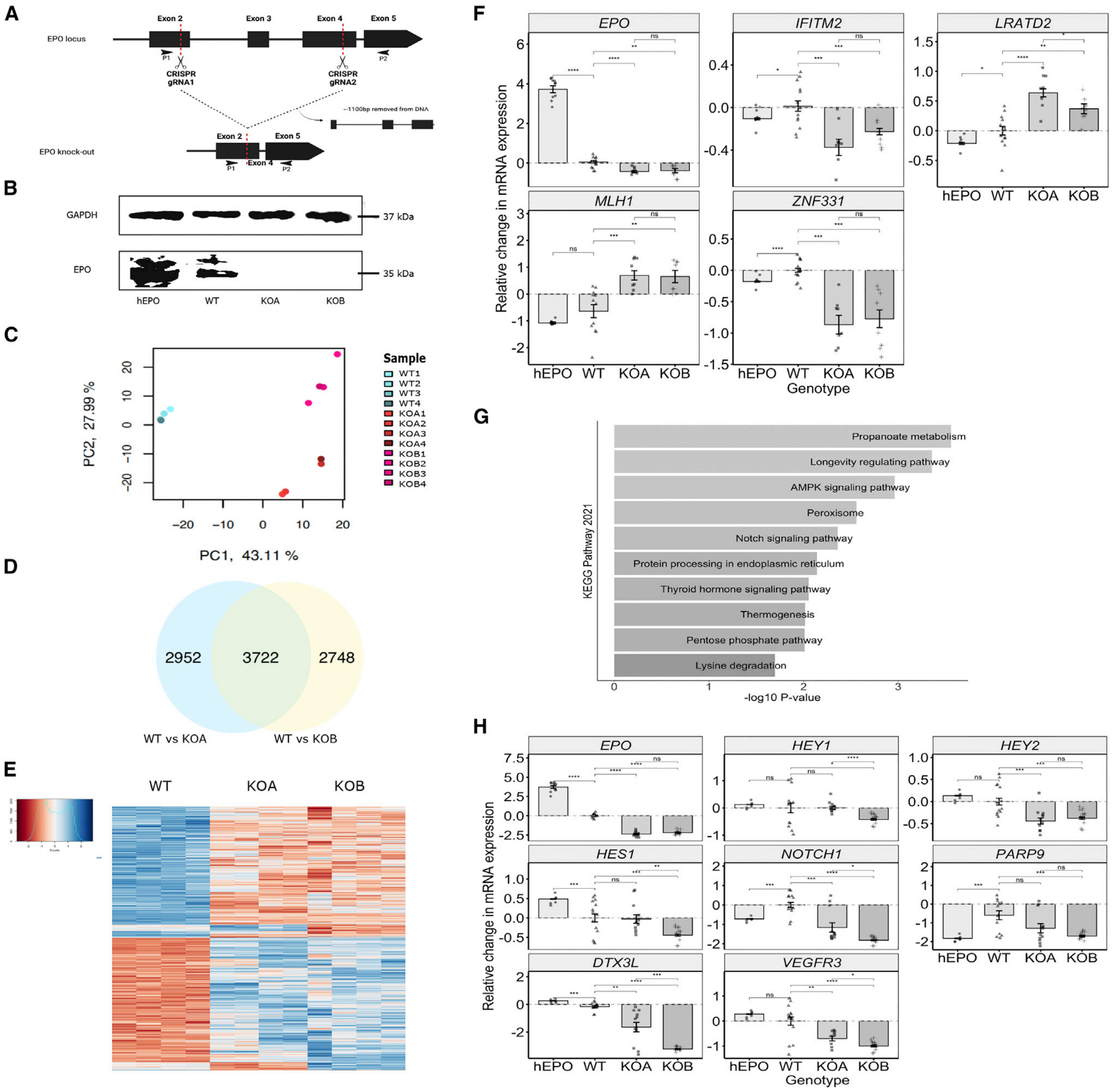

Figure 2. Establishment of whole *EPO* gene knockout in HEK-293 cells using CRISPR-Cas9 gene-editing technology with paired gRNAs and whole transcriptomic profiling of knockouts to identify differentially expressed genes

(A) Hypothetical schematic of the construction of the $EPO^{-/-}$. Successful targeting of the paired gRNAs would result in the introduction of two double-stranded breaks (DSBs) and the removal of the intervening region from the genomic DNA (~645 bp) after DNA repair via non-homologous end joining (NHEJ). The location of the paired gRNAs designed to target the *EPO* gene (ENST00000252723.3) are labeled and highlighted by the red dotted lines. P1 and P2 represent location of primers used for genotyping potential knockouts.

(B) Immunoblotting analysis. Immunoblot analysis, probing for EPO and GAPDH as a control, revealed reduced EPO protein expression levels in both $EPO^{-/-}$ cell lines (KOA and KOB) compared to WT controls and cells transfected with an *EPO* over-expression construct (hEPO).

(C) PCA plot illustrating the transcriptomic profiles of the $EPO^{-/-}$ cell lines compared to WT HEK-293 controls. The plot shows the results obtained from RNA-seq analysis of four WT samples, four KOA samples, and four KOB samples.

(D) The number of differentially expressed genes (DEGs) ($p \le 0.05$) obtained by performing differential gene expression analysis comparing WT to KOA and WT to KOB.

(E) Heatmap illustrating the expression profile of the 3,722 overlapping DEGs. The red blocks represent down-regulated genes, and the blue bocks represent up-regulated genes; the color scale of the heatmap represents the DEG expression level.

(F) qRT-PCR of four of the top DEGs identified by differential gene expression analysis to validate our RNA-seq findings. The graph shows the relative change in mRNA expression levels (+/− SEM) between genotypes.

(G) The top ten KEGG Pathways identified through GEO analysis of the 3,501 overlapping with consistent direction of effects. Analysis was performed with Enrichr online tool. The color scale represents the $\log_{10}$ p value.

(legend continued on next page)

rescaled our genetic association by multiplying by 7.05 (0.44/0.063) to obtain a clinically relevant estimate of the likely impact of genetically predicted therapeutic rises in endogenous EPO on cardiovascular risk. Using this scaling factor allowed us to quantify the upper and lower bounds on the genetically predicted effects of long-term endogenous EPO rises on CVD equivalent to therapeutically, physiologically relevant effects (Figure 4, Table S13). By rescaling the genetic associations to the PHI-induced effects of genetically predicted therapeutic rises in endogenous EPO levels, the odds of disease were 1.01 (95% CI = 0.93, 1.07) for CAD, 0.99 (95% CI = 0.87, 1.15) for MI, and 0.97 (95% CI = 0.87, 1.07) for stroke (Figure 4, Table S13). On the basis of the upper 95% confidence intervals, we could exclude a 1.07, 1.15, and 1.07 increased odds of CAD, MI, or stroke, respectively (Figure 4, Table S13). For the clinical markers predisposing to CVD, we did not observe an association between higher genetically predicted therapeutic rises in endogenous EPO levels and SBP (β [95% CI] = 0.21 [−0.28, 0.78]), DBP (β [95% CI] = −0.42 [−0.71, −0.14]), or resting heart rate (β [95% CI] = −0.42 [−0.78, −0.14]). On the basis of the upper confidence intervals, we could exclude 0.78 mmHg increased SBP levels and any increase in DBP or resting heart rate (Figure 4, Table S13).

The *cis-EPO* SNP is associated with several relevant erythrocyte phenotypes and no unintended effects or diseases

To further determine the specificity of the *cis-EPO* SNP as a genetic instrument for endogenous, physiological EPO levels and identify any potential additional, unintended effects that may be associated with long-term rises in genetically predicted endogenous EPO levels, we tested the association between the *cis-EPO* SNP and 869 traits in up to 451,099 unrelated European UKB individuals. We found the *cis-EPO* SNP was associated with 18 relevant erythrocyte traits ($p < 5 \times 10^{-8}$) with effects between 0.01 and 0.06 SD (Figure S13, Table S14). We also found evidence for an association between the *EPO*-increasing A allele of rs1617640 and decreased fibrosis-4 score ($\beta = -0.01$, $p = 4.7 \times 10^{-17}$) and non-alcoholic fatty acid liver disease (NAFLD) fibrosis score ($\beta = -0.02$, $p = 2.20 \times 10^{-25}$) (Figure S13, Table S14). However, these associations were not clinically significant (equivalent to a 0.06 and 0.07 change in fibrosis-4 or NAFLD for 1 IU/L increase in EPO levels). These associations are most likely driven by the strong association with higher platelet counts ($\beta = 0.02$, $p = 4.7 \times 10^{-39}$). We did not find evidence for an association between genetically predicted higher endogenous EPO levels and other unintended effects or diseases.

Discussion

We have shown how a combination of genetic analyses and functional validation studies provides a powerful approach to assess the therapeutic profile and effects of long-term genetically mediated alterations in drug target levels. Several lines of evidence indicate the *cis-EPO* variant is an excellent proxy to naturally mimic the pharmaceutical effects of raising endogenous EPO levels and test effects on cardiovascular risk. First, we found the A allele of rs1617640 increases EPO levels in a meta-analysis of circulating EPO levels (Table 1). Second, we found the *cis-EPO* SNP is associated with hepatic *EPO* gene expression and, from colocalization analysis, is likely to represent the same causal variant (Table 1, Figure 1). Third, by establishing a heterozygous knockin cell model using CRISPR-Cas9 single-base gene editing, we provided evidence that the *cis-EPO* variant has an allele-specific effect on *EPO* expression levels with homozygotes of the A allele having higher *EPO* expression compared to heterozygotes for the A allele (Figure 3). Our findings help clarify previous studies that found rs1617640 to have an allele-specific effect on *EPO* expression levels. Some had reported the A allele to be associated with higher EPO concentrations,[78,79] while others reported conflicting evidence with the C allele being associated with higher EPO concentrations and promoter activity.[83,84] We found an association between the variant and hepatic *EPO* gene expression; however, the C allele was found to be associated with increased hepatic *EPO* expression while the A allele was found to increase endogenous EPO levels (Table 1). We did not find an association between the variant and renal gene expression despite the CRISPR-Cas9 gene functional work in an embryonic kidney cell line. These differences could be due to lack of power attributable to the small sample size (n = 236 kidneys) compared to the liver dataset. The rs1617640 SNP has different effects depending upon cell type, physiological condition, state, and timing alongside the complex compensatory feedback mechanisms involved in EPO signaling.[85] When testing the *in vitro* effects of *EPO* gene knockdown, we indicate a role for EPO in Notch signaling. The Notch-signaling pathway is known to play a role in cell-cycle signaling, cell-fate specification, and metabolic processes and therefore may represent the broad mitogenic effects of EPO.[86,87] We also found the *cis-EPO* SNP to alter gene expression levels of Notch-signaling genes that were identified through

(H) qRT-PCR of genes involved in the Notch signaling pathway identified by differential gene expression analysis to determine whether $EPO^{-/-}$ results in altered Notch signaling activity. hEPO represents WT HEK-293 cells transfected with an over-expression *EPO* construct. The graph shows the relative change in mRNA expression levels (+/− SEM) between genotypes. Columns and error bars in (F) and (H) represent mean and SEM values. Paired t test was performed. ns, non-significant; *p ≤ 0.05, **p ≤ 0.01, ***p ≤ 0.001, ****p ≤ 0.0001.

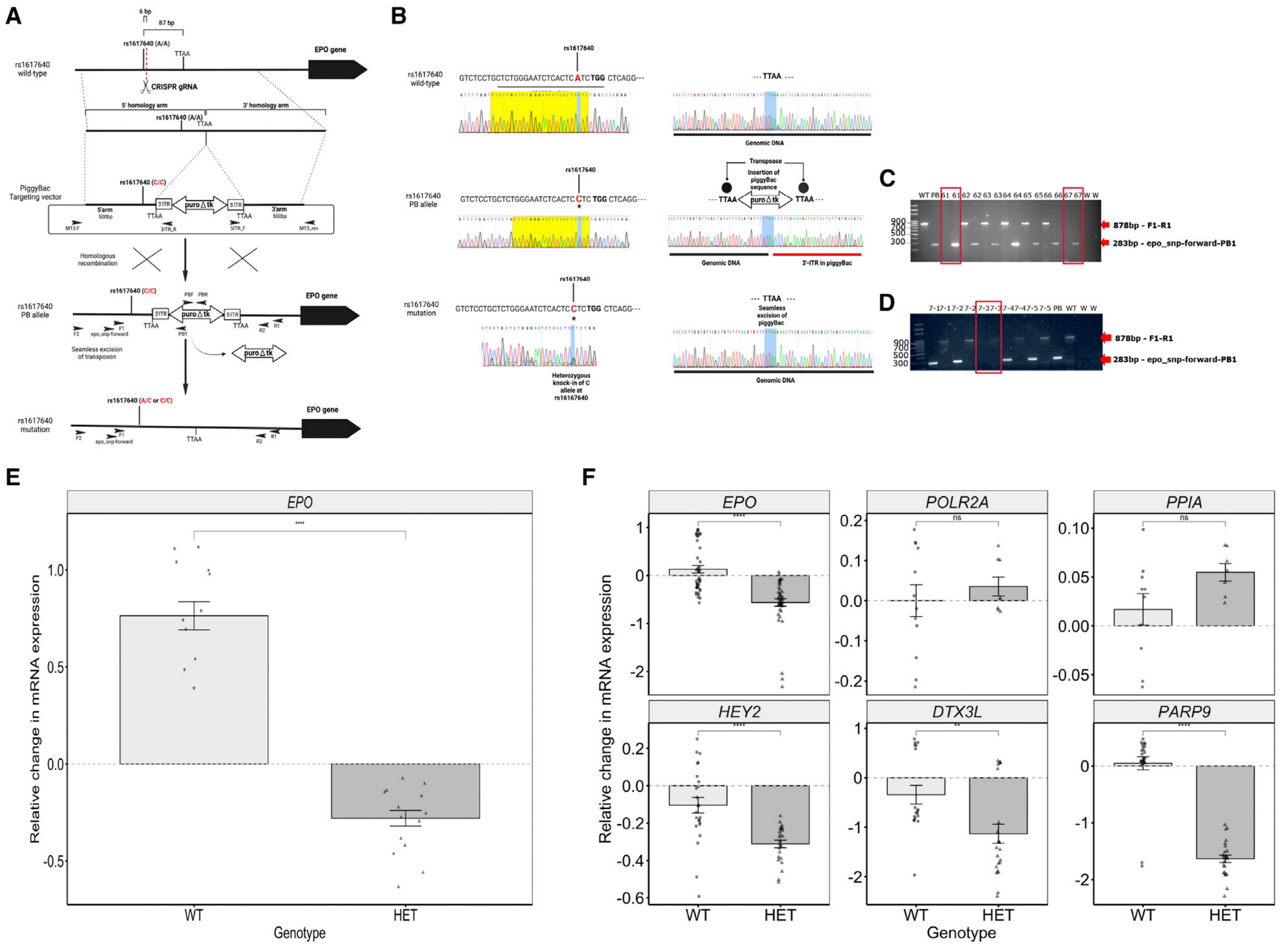

Figure 3. Establishment of heterozygous knockin at rs1617640 (A/C) in HEK-293 cells using CRISPR-Cas9 gene-editing technology alongside *piggyBac* transposon system

(A) Schematic of the generation of a SNP knockin model of rs1617640 using CRISPR-Cas9 gene editing alongside the *piggyBac* transposon system. The location of rs1617640 (A/A) and the site at which the gRNA is designed to introduce a DSB within the wild-type sequence of HEK-293 cells is shown in the top panel. Two homology arms either side of the closest TTAA site are designed with one (5′ arm) containing the desired gene edit at rs1617640 (C/C). The two homology arms are cloned into the targeting construct either side of the *piggyBac* transposon carrying the selectable markers, *puroΔtk*. Upon introduction of a DSB at the site targeted by the gRNA, DNA repair via homologous recombination due to the presence of the homologous arms is initiated and the the *piggyBac* transposon becomes integrated into the genomic DNA at the TTAA site. After selection with puromycin, clones with mutation-corrected lines were identified and transiently transfected with *piggyBac* transposase plasmid, followed by FIAU treatment to eliminate *piggyBac*-containing clones. Mutation-corrected heterozygous clones for rs1617640 (A/C genotype) were isolated with no marks remaining within the genomic DNA.
(B) Sanger sequencing results of the sequence at each stage of the CRISPR-Cas9 and *piggyBac* transposon technique. The top panel represents the wild-type sequence at rs1617640 and the wild-type sequences up- and downstream of the TTAA site prior to gene editing. The middle panel represents the mutated sequence at rs1617640 and the insertion of the *piggyBac* transposon sequence at the TTAA site following homologous recombination after successful introduction of the DSB. The bottom panel represents the seamless excision of the *piggyBac* transposon from the genomic DNA. The yellow highlighted region represents the gRNA sequence.
(C) PCR gel electrophoresis confirming the insertion of the *piggyBac* transposon in clones 61 and 67.
(D) PCR gel electrophoresis confirming successful removal of the *piggyBac* transposon from the genome following treatment with the transposase in clone 7-3.
(E) qRT-PCR of *EPO* to validate rs1617640 as causal in controlling *EPO* mRNA expression levels. The graph shows the relative change in mRNA expression levels (+/− SEM) between genotypes (A/A and A/C).
(F) qRT-PCR of genes involved in the Notch signaling pathway (*HEY2*, *DTX3L*, and *PARP9)* and two control genes (*PPIA* and *POLR2A*). *EPO* was repeated again as a positive control for altered expression for comparison. The graph shows the relative change in mRNA expression levels (+/− SEM) between genotypes. Columns and error bars in (E) and (F) represent mean and SEM values. Paired t test was performed. ns, non-significant; *$p \leq 0.05$, **$p \leq 0.01$, ***$p \leq 0.001$, ****$p \leq 0.0001$.

whole-transcriptomic profiling of $EPO^{-/-}$ cell lines, indicating that rs1617640 is important in controlling *EPO* expression levels and in downstream implicated pathways involved in cell-cycle activity, DNA repair, and metabolic processes (Figures 2 and 3).

Having provided genetic and functional evidence that a *cis-EPO* variant alters *EPO* gene expression and circulating protein levels, we used this variant as a genetic predictor for long-term therapeutic modulation of EPO levels to show that genetically predicted higher endogenous EPO

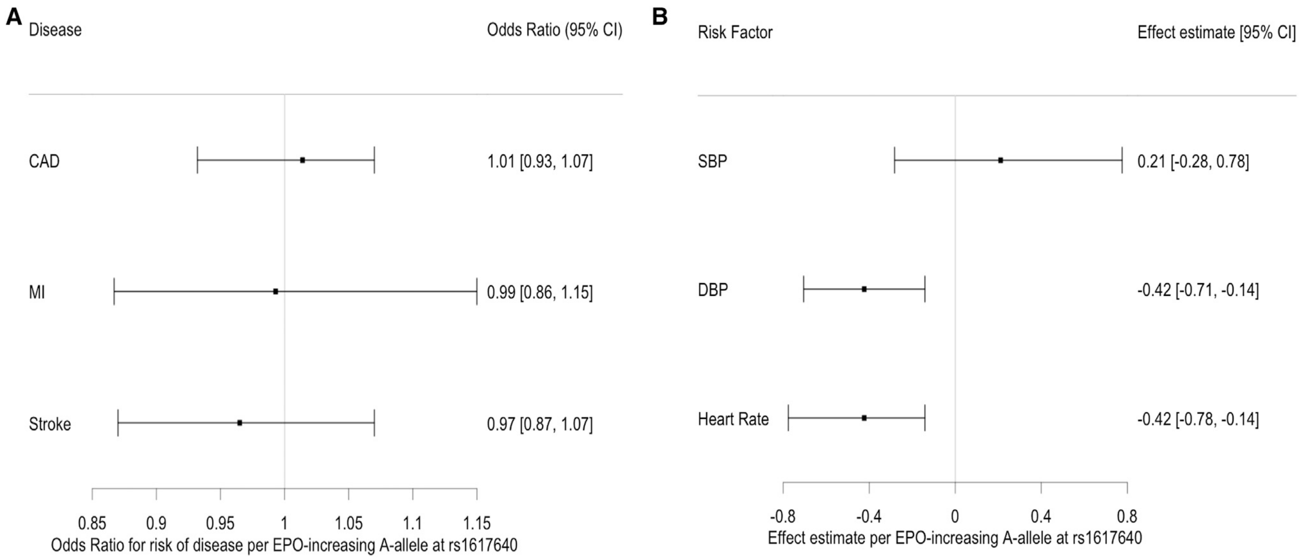

Figure 4. Genetically predicted therapeutic rises in endogenous EPO levels are not associated with an increased risk of CVD or clinical markers for CVD risk factors

We rescaled the genetic estimates obtained through two-sample MR by using the *cis-EPO* SNP to genetically estimate the effect of therapeutic rises in endogenous EPO levels to the PHI-induced effect reported in a phase II RCT. The black point on the plot represents the odds ratio and the bars represent the 95% confidence intervals.For all other figures, elements have been defined in the corresponding figure legends.

(A) On the basis of the upper confidence interval, we were able to exclude increased odds of 1.07, 1.15, and 1.07 for CAD, MI, or stroke, respectively with 2.2 IU/L genetically mediated higher endogenous EPO levels.

(B) On the basis of the upper confidence interval, we were also able to exclude levels higher than 0.21 mmHg for SBP and no increase in DBP or resting heart rate with genetically mediated therapeutic higher endogenous EPO levels.

levels (equivalent to 5.1 IU/L) are not associated with increased cardiovascular risk or elevated values of clinical markers (SBP, DBP, or resting heart rate) predisposing to CVD risk factors (e.g., hypertension). To obtain a more representative effect of therapeutically altered endogenous EPO levels, we rescaled these genetic effects to the PHI-induced effects on endogenous EPO levels from a fixed dose phase II trial[72] and did not observe an association between genetically predicted therapeutic rises in endogenous EPO levels and risk of CVD (Figure 4). Using the upper bound of the confidence interval, we were able to statistically exclude increased odds of 1.07, 1.15, and 1.07 for CAD, MI, or stroke, respectively, and 0.78 mmHg increased levels of SBP (Figure 4). We were able to exclude adverse effects on DBP and heart rate (Figure 4). Our results are consistent with the hypothesis that PHIs are not likely to increase CVD risk or CVD risk factors in treating anemia of CKD when increasing circulating EPO levels within the physiological range. Scaling genetic estimates to the effect of a fixed dose after 4 weeks of treatment may not be the most clinically relevant because PHIs require titration to a hemoglobin target, but this was the best estimate available, as changes in EPO levels were only measured in the one fixed dose phase II trial.[72] Because all PHIs work through the same mechanism, these genetically predicted effects of therapeutically altering endogenous EPO levels are likely applicable to all PHI compounds. Any slight differences in effects on EPO levels and CVD risk between HIF-PHI compounds would most likely be related to independent biochemical properties of the compounds, variations in dosages, and the effects of PHIs on transcription of other hypoxic response genes, which were not investigated in this study.

There are some limitations to our study. As with any study using human genetics as predictors, our results cannot rule out effects but instead can provide upper bounds on their probability.[88] First, here we performed the genetic analyses in a general, "healthy" population as opposed to a diseased cohort in whom the treatment is used. Diseased populations may respond differently to that estimated by the genetic association as a result of having variable baseline EPO levels or additional underlying conditions.[89,90] Despite rescaling the genetic effect to the PHI-induced effect to try and overcome this, we still assume linearity, which may not be the case for the therapy. For example, MR estimates could change depending on baseline levels, and therefore inferences about the likely effect at the individual level need careful consideration, particularly when doses are titrated.[90] As larger studies become available, particularly in disease cohorts, our ability to detect associations and perform stratified analyses will increase. We will become more confident about the conclusions drawn from these types of investigations.[90,91] Second, common genetic variants differ from clinical trials in that they represent subtle, life-long perturbations, whereas clinical trials test more acute larger changes.[92,93] In addition, therapeutic interventions may result in localized effects at a particular time, titration to a particular level resulting in different

individual-level biomarker increases, or may only be efficacious in a certain physiological state, which is difficult to accurately predict using genetics.[89,93] Third, as with any MR investigation, pleiotropy could lead to biased estimates. As we only used a single genetic instrument, we were unable to fully test for horizontal pleiotropy using established statistical methods and it remains possible that our MR findings are confounded by the variant's association with *TFR2*, as well as *EPO*. The presence of the variant in the *EPO* promoter, and the alteration of *EPO* expression, but not *TFR2* expression, in our single-base editing experiments suggests *EPO* is the main target. The single-base editing enabled separation of the primary effect of the *cis*-SNP on *EPO* from a likely secondary effect on *TFR2* (Figure S12) eliminating some concerns of biased estimates due to horizontal pleiotropy.

In summary, this study indicates that genetically predicted long-term rises in endogenous EPO levels do not increase cardiovascular risk, with upper limits of 1.07, 1.15, and 1.07 for CAD, MI, and stroke, respectively, given a clinically relevant 2.2 unit rise in endogenous EPO levels. These estimates were established using extremely large case-control studies. Our functional evidence using CRISPR-Cas9 and the *piggyBac* system to change the allele at rs1617640 validated the *cis-EPO* SNP as a partial proxy for therapeutically altered endogenous EPO. We have shown how genetic analyses combined with functional validation studies represent a powerful approach to identify relevant genetic markers that can investigate the long-term effect of therapeutic action.

Data and code availability

The code generated during this study are available at https://github.com/CharliHarlow/EPO_metaanalysis_rnaseq_MR_phewas. The accession number for the summary-level EPO meta-analysis data generated in this paper is Zenodo: 6811853 (https://zenodo.org/record/6811853). The accession number for the raw sequencing read data generated in this paperr is Zenodo: 6811704 (https://zenodo.org/record/6811704#.Ys1Jzy2ZPOQ). The published article includes the rest of the data generated or analyzed during this study.

Supplemental information

Supplemental information can be found online at https://doi.org/10.1016/j.ajhg.2022.08.004.

Acknowledgments

The authors acknowledge all participants and investigators involved in the different studies. The HealthABC study was supported by National Institute on Aging (NIA) contracts (N01-AG-6-2101; N01-AG-6-2103; N01-AG-6-2106; NIA grant R01-AG0280502, and NINR grant R01-NR012459) and was funded in part by the Intramural Research Program of the NIA. The InCHIANTI study baseline (1998–2000) was supported as a "targeted project" (ICS110.1/RF97.71) by the Italian Ministry of Health and in part by the NIA (contracts: 263 MD 9164 and 263 MD 821336). The research utilized data from the UK Biobank (UKB) resource carried out under application number 9072. UKB protocols were approved by the National Research Ethics Service Committee. The authors thank the Exeter Sequencing service in carrying out the RNA-sequencing. The equipment utilized is funded by the Wellcome Trust Institutional Strategic Support Fund (WT097835MF), Wellcome Trust Multi User Equipment Award (WT101650MA), and BBSRC LOLA award (BB/K003240/1). The authors acknowledge the use of the University of Exeter High-Performance Computing (HPC) facility in carrying out this work funded by an MRC Clinical Research Infrastructure award (MRC grant: MR/M008924/1). T.M.F. is supported by MRC awards MR/WO14548/1 and MR/T002239/1. J.T is funded by an Academy of Medical Sciences grant. pLV-EF1alpha-EPO-218-PDGFR was a gift from Tao Liu. The authors thank Akshay Bhinge for providing the *piggyBac* plasmid and transposase plasmid, Dario Pacitti for his help with the RNA-seq analysis, and Natsuki Takamura and Yuriko Iizuka for help in the laboratory. For the purpose of open access, the author has applied a CC BY public copyright license to any Author Accepted Manuscript version arising from this submission.

Declaration of interests

C.E.H. was awarded an MRC iCASE studentship (MR/P016065/1), which was cofunded by GSK and the MRC for the duration of the study. GSK are undertaking clinical development into a novel PHI and have given permission to publish this work alongside an internal review of the manuscript. A.Y.C. is an employee of GSK, is a shareholder of GSK stock, and was involved in the study design, interpretation of the data, and writing of the paper. V.V.P. was an employee of GSK at the time of the study conception and design. He was involved in interpreting the data and writing of the paper and is a shareholder of GSK and Roche Holding AG stocks. D.M.W. was an employee of GSK at the time of the study conception and design and was involved in the interpretation of the data and writing of the paper. H.L.L. and M.A.N. received support from a consulting contract between Data Tecnica International and the National Institute on Aging (NIA), National Institutes of Health (NIH). They were involved in data analysis and review of the manuscript. N.V. is a full-time employee of Regeneron Pharmaceutical Inc. and receives stock options and restricted stock units as compensation. D.D.J. received support from the Center of Human Health and the Environment P30ESO25128 grant. F.I. is an AbbVie employee and receives stocks from AbbVie, which has not been involved in this work at any level.

Received: April 14, 2022
Accepted: August 3, 2022
Published: September 1, 2022

Web resources

AddGene, https://www.addgene.org
Benchling, https://www.benchling.com
BioRender, https://biorender.com
BLAST, https://www.ensembl.org/Multi/Tools/Blast
Ensembl, https://www.ensembl.org/index.html
Enrichr, https://maayanlab.cloud/Enrichr/
GEMMA, https://www.xzlab.org/software/GEMMAmanual.pdf

Genewiz, https://www.genewiz.com/en-GB/
GTEx, https://gtexportal.org/
Human Cell Atlas, https://www.humancellatlas.org
METAL, https://genome.sph.umich.edu/wiki/METAL_Quick_Start#Meta-Analysis_Results
RefFinder, https://www.heartcure.com.au/reffinder/?type=reference
RStudio, https://www.rstudio.com
Stata, https://www.stata.com

References

1. Stauffer, M.E., and Fan, T. (2014). Prevalence of anemia in chronic kidney disease in the United States. PLoS One *9*, e84943.
2. Hill, N.R., Fatoba, S.T., Oke, J.L., Hirst, J.A., O'Callaghan, C.A., Lasserson, D.S., and Hobbs, F.D.R. (2016). Global Prevalence of Chronic Kidney Disease – A Systematic Review and Meta-Analysis. PLoS One *11*, e0158765.
3. St Peter, W.L., Guo, H., Kabadi, S., Gilbertson, D.T., Peng, Y., Pendergraft, T., and Li, S. (2018). Prevalence, treatment patterns, and healthcare resource utilization in Medicare and commercially insured non-dialysis-dependent chronic kidney disease patients with and without anemia in the United States. BMC Nephrol. *19*, 67.
4. Zheng, Q., Wang, Y., Yang, H., Sun, L., Fu, X., Wei, R., Liu, Y.N., and Liu, W.J. (2020). Efficacy and Safety of Daprodustat for Anemia Therapy in Chronic Kidney Disease Patients: A Systematic Review and Meta-Analysis. Front. Pharmacol. *11*, 573645.
5. Cases, A., Egocheaga, M.I., Tranche, S., Pallarés, V., Ojeda, R., Górriz, J.L., and Portolés, J.M. (2018). Anemia of chronic kidney disease: Protocol of study, management and referral to Nephrology. Nefrologia *38*, 8–12.
6. Fishbane, S., and Spinowitz, B. (2018). Update on Anemia in ESRD and Earlier Stages of CKD: Core Curriculum 2018. Am. J. Kidney Dis. *71*, 423–435.
7. Jelkmann, W. (2013). Physiology and pharmacology of erythropoietin. Transfus. Med. Hemother. *40*, 302–309.
8. Pfeffer, M.A., Burdmann, E.A., Chen, C.Y., Cooper, M.E., de Zeeuw, D., Eckardt, K.U., Feyzi, J.M., Ivanovich, P., Kewalramani, R., Levey, A.S., et al. (2009). A Trial of Darbepoetin Alfa in Type 2 Diabetes and chronic kidney disease. N. Engl. J. Med. *361*, 2019–2032.
9. Babitt, J.L., and Lin, H.Y. (2012). Mechanisms of anemia in CKD. J. Am. Soc. Nephrol. *23*, 1631–1634.
10. Bonomini, M., Del Vecchio, L., Sirolli, V., and Locatelli, F. (2016). New treatment approaches for the anemia of CKD. Am. J. Kidney Dis. *67*, 133–142.
11. Parfrey, P. (2021). Hypoxia-Inducible Factor Prolyl Hydroxylase Inhibitors for Anemia in CKD. N. Engl. J. Med. *385*, 2390–2391. https://doi.org/10.1056/NEJMe2117100.
12. Kaplan, J.M., Sharma, N., and Dikdan, S. (2018). Hypoxia-inducible factor and its role in the management of anemia in chronic kidney disease. Int. J. Mol. Sci. *19*, E389.
13. Haase, V.H. (2013). Regulation of erythropoiesis by hypoxia-inducible factors. Blood Rev. *27*, 41–53.
14. Sugahara, M., Tanaka, T., and Nangaku, M. (2017). Prolyl hydroxylase domain inhibitors as a novel therapeutic approach against anemia in chronic kidney disease. Kidney Int. *92*, 306–312.
15. Singh, A.K., Carroll, K., McMurray, J.J.V., Solomon, S., Jha, V., Johansen, K.L., Lopes, R.D., Macdougall, I.C., Obrador, G.T., Waikar, S.S., et al. (2021). Daprodustat for the treatment of anemia in patients not undergoing dialysis. N. Engl. J. Med. *385*, 2313–2324. https://doi.org/10.1056/NEJMoa2113380.
16. Singh, A.K., Carroll, K., Perkovic, V., Solomon, S., Jha, V., Johansen, K.L., Lopes, R.D., Macdougall, I.C., Obrador, G.T., Waikar, S.S., et al. (2021). Daprodustat for the treatment of anemia in pateints undergoing dialysis. N. Engl. J. Med. *385*, 2325–2335. https://doi.org/10.1056/NEJMoa2113379.
17. Eckardt, K.-U., Agarwal, R., Aswad, A., Awad, A., Block, G.A., Bacci, M.R., Farag, Y.M.K., Fishbane, S., Hubert, H., Jardine, A., et al. (2021). Safety and efficacy of vadadustat for anemia in patients undergoing dialysis. N. Engl. J. Med. *384*, 1601–1612.
18. Chertow, G.M., Pergola, P.E., Farag, Y.M.K., Agarwal, R., Arnold, S., Bako, G., Block, G.A., Burke, S., Castillo, F.P., Jardine, A.G., et al. (2021). Vadadustat in patients with anemia and non–dialysis-dependent CKD. N. Engl. J. Med. *384*, 1589–1600.
19. Chen, N., Hao, C., Peng, X., Lin, H., Yin, A., Hao, L., Tao, Y., Liang, X., Liu, Z., Xing, C., et al. (2019). Roxadustat for anemia in patients with kidney disease not receiving dialysis. N. Engl. J. Med. *381*, 1001–1010.
20. Fishbane, S., El-Shahawy, M.A., Pecoits-Filho, R., Van, B.P., Houser, M.T., Frison, L., Little, D.J., Guzman, N.J., and Pergola, P.E. (2021). Roxadustat for treating anemia in patients with CKD not on dialysis: results from a randomized phase 3 study. J. Am. Soc. Nephrol. *32*, 737–755.
21. Akizawa, T., Iwasaki, M., Yamaguchi, Y., Majikawa, Y., and Reusch, M. (2020). Phase 3, Randomized, Double-Blind, Active-Comparator (Darbepoetin Alfa) Study of Oral Roxadustat in CKD Patients with Anemia on Hemodialysis in Japan. J. Am. Soc. Nephrol. *31*, 1628–1639.
22. Akizawa, T., Iwasaki, M., Otsuka, T., Yamaguchi, Y., and Reusch, M. (2021). Phase 3 study of roxadustat to treat anemia in non–dialysis-dependant CKD. Kidney Int. Rep. *6*, 1810–1828.
23. Chen, N., Hao, C., Liu, B.C., Lin, H., Wang, C., Xing, C., Liang, X., Jiang, G., Liu, Z., Li, X., et al. (2019). Roxadustat treatment for anemia in patients undergoing long-term dialysis. N. Engl. J. Med. *381*, 1011–1022.
24. Akizawa, T., Nangaku, M., Yonekawa, T., Okuda, N., Kawamatsu, S., Onoue, T., Endo, Y., Hara, K., and Cobitz, A.R. (2020). Efficacy and safety of daprodustat compared with darbepoetin alfa in japanese hemodialysis patients with anemia: a randomized, double-blind, phase 3 trial. Clin. J. Am. Soc. Nephrol. *15*, 1155–1165.
25. Kanai, H., Nangaku, M., Nagai, R., Okuda, N., Kurata, K., Nagakubo, T., Endo, Y., and Cobitz, A. (2021). Efficacy and safety of daprodustat in Japanese peritoneal dialysis patients. Ther. Apher. Dial. *25*, 979–987. https://doi.org/10.1111/1744-9987.13686.
26. Dhillon, S. (2020). First approval. Drugs *80*, 1491–1497.
27. Gill, D., Georgakis, M.K., Koskeridis, F., Jiang, L., Feng, Q., Wei, W.Q., Theodoratou, E., Elliott, P., Denny, J.C., Malik, R., et al. (2019). Use of genetic variants related to antihypertensive drugs to inform on efficacy and side effects. Circulation *140*, 270–279.
28. Nelson, M.R., Tipney, H., Painter, J.L., Shen, J., Nicoletti, P., Shen, Y., Floratos, A., Sham, P.C., Li, M.J., Wang, J., et al. (2015). The support of human genetic evidence for approved drug indications. Nat. Genet. *47*, 856–860.

29. Nguyen, P.A., Born, D.A., Deaton, A.M., Nioi, P., and Ward, L.D. (2019). Phenotypes associated with genes encoding drug targets are predictive of clinical trial side effects. Nat. Commun. *10*, 2132.
30. Plenge, R.M., Scolnick, E.M., and Altshuler, D. (2013). Validating therapeutic targets through human genetics. Nat. Rev. Drug Discov. *12*, 581–594.
31. Lotta, L.A., Sharp, S.J., Burgess, S., Perry, J.R.B., Stewart, I.D., Willems, S.M., Luan, J., Ardanaz, E., Arriola, L., Balkau, B., et al. (2016). Association between low-density lipoprotein cholesterol-lowering genetic variants and risk of type 2 diabetes: a meta-analysis. JAMA *316*, 1383–1391.
32. Scott, R.A., Freitag, D.F., Li, L., Chu, A.Y., Surendran, P., Young, R., Grarup, N., Stancáková, A., Chen, Y., Varga, T.V., et al. (2016). A genomic approach to therapeutic target validation identifies a glucose-lowering GLP1R variant protective for coronary heart disease. Sci. Transl. Med. *8*, 341ra76.
33. Swerdlow, D.I., Preiss, D., Kuchenbaecker, K.B., Holmes, M.V., Engmann, J.E.L., Shah, T., Sofat, R., Stender, S., Johnson, P.C.D., Scott, R.A., et al. (2015). HMG-coenzyme A reductase inhibition, type 2 diabetes, and bodyweight: evidence from genetic analysis and randomised trials. Lancet (London, England) *385*, 351–361.
34. Melzer, D., Perry, J.R.B., Hernandez, D., Corsi, A.M., Stevens, K., Rafferty, I., Lauretani, F., Murray, A., Gibbs, J.R., Paolisso, G., et al. (2008). A Genome-Wide Association Study Identifies Protein Quantitative Trait Loci (pQTLs). PLoS Genet. *4*, e1000072.
35. Walker, V.M., Davey Smith, G., Davies, N.M., and Martin, R.M. (2017). Mendelian randomization: a novel approach for the prediction of adverse drug events and drug repurposing opportunities. Int. J. Epidemiol. *46*, 2078–2089.
36. Swerdlow, D.I., Kuchenbaecker, K.B., Shah, S., Sofat, R., Holmes, M.V., White, J., Mindell, J.S., Kivimaki, M., Brunner, E.J., Whittaker, J.C., et al. (2016). Selecting instruments for Mendelian randomization in the wake of genome-wide association studies. Int. J. Epidemiol. *45*, 1600–1616.
37. Davey Smith, G., and Hemani, G. (2014). Mendelian randomization: genetic anchors for causal inference in epidemiological studies. Hum. Mol. Genet. *23*, R89–R98.
38. Lichou, F., and Trynka, G. (2020). Functional studies of GWAS variants are gaining momentum. Nat. Commun. *11*, 6283.
39. Wang, H., La Russa, M., and Qi, L.S. (2016). CRISPR/Cas9 in Genome Editing and Beyond. Annu. Rev. Biochem. *85*, 227–264.
40. Yang, L., Guell, M., Byrne, S., Yang, J.L., De Los Angeles, A., Mali, P., Aach, J., Kim-Kiselak, C., Briggs, A.W., Rios, X., et al. (2013). Optimization of scarless human stem cell genome editing. Nucleic Acids Res. *41*, 9049–9061.
41. Zhou, X., and Stephens, M. (2012). Genome-wide efficient mixed-model analysis for association studies. Nat. Genet. *44*, 821–824.
42. Willer, C.J., Li, Y., and Abecasis, G.R. (2010). METAL: fast and efficient meta-analysis of genome-wide association scans. Bioinformatics *26*, 2190–2191.
43. Yang, J., Lee, S.H., Goddard, M.E., and Visscher, P.M. (2011). GCTA: a tool for genome-wide complex trait analysis. Am. J. Hum. Genet. *88*, 76–82.
44. Yang, J., Ferreira, T., Morris, A.P., Medland, S.E., Genetic Investigation of ANthropometric Traits GIANT Consortium; and DIAbetes Genetics Replication And Meta-analysis DIAGRAM Consortium, Madden, P.A.F., Heath, A.C., Martin, N.G., Montgomery, G.W., et al. (2012). Conditional and joint multiple-SNP analysis of GWAS summary statistics identifies additional variants influencing complex traits. Nat. Genet. *44*, 369–375.
45. Etheridge, A.S., Gallins, P.J., Jima, D., Broadaway, K.A., Ratain, M.J., Schuetz, E., Schadt, E., Schroder, A., Molony, C., Zhou, Y., et al. (2020). A new liver expression quantitative trait locus map from 1, 183 individuals provides evidence for novel expression quantitative trait loci of drug response, metabolic, and sex-biased phenotypes. Clin. Pharmacol. Ther. *107*, 1383–1393.
46. Damman, J., Bloks, V.W., Daha, M.R., van der Most, P.J., Sanjabi, B., van der Vlies, P., Snieder, H., Ploeg, R.J., Krikke, C., Leuvenink, H.G.D., and Seelen, M.A. (2015). Hypoxia and complement-and-coagulation pathways in the deceased organ donor as the major target for intervention to improve renal allograft outcome. Transplantation *99*, 1293–1300.
47. Giambartolomei, C., Vukcevic, D., Schadt, E.E., Franke, L., Hingorani, A.D., Wallace, C., and Plagnol, V. (2014). Bayesian Test for Colocalisation between Pairs of Genetic Association Studies Using Summary Statistics. PLoS Genet. *10*, e1004383.
48. Wallace, C. (2020). Eliciting priors and relaxing the single causal variant assumption in colocalisation analyses. PLoS Genet. *16*, e1008720.
49. Liu, T., Jia, P., Ma, H., Reed, S.A., Luo, X., Larman, H.B., and Schultz, P.G. (2017). Construction and screening of a lentiviral secretome library. Cell Chem. Biol. *24*, 767–771.e3.
50. Livak, K.J., and Schmittgen, T.D. (2001). Analysis of Relative Gene Expression Data Using Real-Time Quantitative PCR and the $2-\Delta\Delta CT$ Method. Methods *25*, 402–408.
51. Xie, F., Xiao, P., Chen, D., Xu, L., and Zhang, B. (2012). miRDeepFinder: a miRNA analysis tool for deep sequencing of plant small RNAs. Plant Mol. Biol. *80*, 75–84. https://doi.org/10.1007/s11103-012-9885-2.
52. RStudio Team (2018). RStudio: Integrated Development for R. (PBC, Boston, MA: RStudio). https://www.rstudio.com/.
53. Schroeder, A., Mueller, O., Stocker, S., Salowsky, R., Leiber, M., Gassmann, M., Lightfoot, S., Menzel, W., Granzow, M., and Ragg, T. (2006). The RIN: an RNA integrity number for assigning integrity values to RNA measurements. BMC Mol. Biol. *7*, 3.
54. Ewels, P., Magnusson, M., Lundin, S., and Käller, M. (2016). MultiQC: summarize analysis results for multiple tools and samples in a single report. Bioinformatics *32*, 3047–3048.
55. Martin, M. (2011). Cutadapt removes adapter sequences from high-throughput sequencing reads. EMBnet. J. *17*, 10. Next Gener. Seq. Data Anal.. https://doi.org/10.14806/ej.17.1.200
56. Dobin, A., Davis, C.A., Schlesinger, F., Drenkow, J., Zaleski, C., Jha, S., Batut, P., Chaisson, M., and Gingeras, T.R. (2013). STAR: ultrafast universal RNA-seq aligner. Bioinformatics *29*, 15–21.
57. Liao, Y., Smyth, G.K., and Shi, W. (2014). featureCounts: an efficient general purpose program for assigning sequence reads to genomic features. Bioinformatics *30*, 923–930.
58. Love, M.I., Huber, W., and Anders, S. (2014). Moderated estimation of fold change and dispersion for RNA-seq data with DESeq2. Genome Biol. *15*, 550.
59. Chen, E.Y., Tan, C.M., Kou, Y., Duan, Q., Wang, Z., Meirelles, G.V., Clark, N.R., and Ma'ayan, A. (2013). Enrichr: interactive and collaborative HTML5 gene list enrichment analysis tool. BMC Bioinf. *14*, 128.
60. Kuleshov, M.V., Jones, M.R., Rouillard, A.D., Fernandez, N.F., Duan, Q., Wang, Z., Koplev, S., Jenkins, S.L., Jagodnik, K.M., Lachmann, A., et al. (2016). Enrichr: a comprehensive gene

set enrichment analysis web server 2016 update. Nucleic Acids Res. *44*, W90–W97.
61. Xie, Z., Bailey, A., Kuleshov, M.V., Clarke, D.J.B., Evangelista, J.E., Jenkins, S.L., Lachmann, A., Wojciechowicz, M.L., Kropiwnicki, E., Jagodnik, K.M., et al. (2021). Gene set knowledge discovery with enrichr. Curr. Protoc. *1*, e90.
62. Oguro-Ando, A., Rosensweig, C., Herman, E., Nishimura, Y., Werling, D., Bill, B.R., Berg, J.M., Gao, F., Coppola, G., Abrahams, B.S., and Geschwind, D.H. (2015). Increased CYFIP1 dosage alters cellular and dendritic morphology and dysregulates mTOR. Mol. Psychiatry *20*, 1069–1078.
63. Chin, C.L., Goh, J.B., Srinivasan, H., Liu, K.I., Gowher, A., Shanmugam, R., Lim, H.L., Choo, M., Tang, W.Q., Tan, A.H.M., et al. (2019). A human expression system based on HEK293 for the stable production of recombinant erythropoietin. Sci. Rep. *9*, 16768.
64. Nikpay, M., Goel, A., Won, H.H., Hall, L.M., Willenborg, C., Kanoni, S., Saleheen, D., Kyriakou, T., Nelson, C.P., Hopewell, J.C., et al. (2015). A comprehensive 1, 000 Genomes-based genome-wide association meta-analysis of coronary artery disease. Nat. Genet. *47*, 1121–1130.
65. Iwaki, T., and Umemura, K. (2011). A single plasmid transfection that offers a significant advantage associated with puromycin selection, fluorescence-assisted cell sorting, and doxycycline-inducible protein expression in mammalian cells. Cytotechnology *63*, 337–343.
66. Yusa, K. (2013). Seamless genome editing in human pluripotent stem cells using custom endonuclease–based gene targeting and the piggyBac transposon. Nat. Protoc. *8*, 2061–2078.
67. Malik, R., Chauhan, G., Traylor, M., Sargurupremraj, M., Okada, Y., Mishra, A., Rutten-Jacobs, L., Giese, A.K., van der Laan, S.W., Gretarsdottir, S., et al. (2018). Multiancestry genome-wide association study of 520, 000 subjects identifies 32 loci associated with stroke and stroke subtypes. Nat. Genet. *50*, 524–537.
68. Harris, R.J., Deeks, J.J., Altman, D.G., Bradburn, M.J., Harbord, R.M., and Sterne, J.A.C. (2008). metan: fixed- and random-effects meta-analysis. STATA J. *8*, 3–28.
69. Burgess, S., Small, D.S., and Thompson, S.G. (2017). A review of instrumental variable estimators for Mendelian randomization. Stat. Methods Med. Res. *26*, 2333–2355.
70. Wain, L.V., Vaez, A., Jansen, R., Joehanes, R., Van Der Most, P.J., Erzurumluoglu, A.M., O'Reilly, P.F., Cabrera, C.P., Warren, H.R., Rose, L.M., and Verwoert, G.C. (2017). Novel Blood Pressure Locus and Gene Discovery Using Genome-Wide Association Study and Expression Data Sets From Blood and the Kidney. Hypertension *70*, e4–e19. https://doi.org/10.1161/hypertensionaha.117.09438.
71. den Hoed, M., Eijgelsheim, M., Esko, T., Brundel, B.J.J.M., Peal, D.S., Evans, D.M., Nolte, I.M., Segrè, A.V., Holm, H., Handsaker, R.E., et al. (2013). Identification of heart rate-associated loci and their effects on cardiac conduction and rhythm disorders. Nat. Genet. *45*, 621–631.
72. Meadowcroft, A.M., Cizman, B., Holdstock, L., Biswas, N., Johnson, B.M., Jones, D., Nossuli, A.K., Lepore, J.J., Aarup, M., and Cobitz, A.R. (2019). Daprodustat for anemia: A 24-week, open-label, randomized controlled trial in participants on hemodialysis. Clin. Kidney J. *12*, 139–148.
73. Loh, P.-R., Tucker, G., Bulik-Sullivan, B.K., Vilhjálmsson, B.J., Finucane, H.K., Salem, R.M., Chasman, D.I., Ridker, P.M., Neale, B.M., Berger, B., et al. (2015). Efficient Bayesian mixed-model analysis increases association power in large cohorts. Nat. Genet. *47*, 284–290.
74. Frayling, T.M., Beaumont, R.N., Jones, S.E., Yaghootkar, H., Tuke, M.A., Ruth, K.S., Casanova, F., West, B., Locke, J., Sharp, S., et al. (2018). A common allele in FGF21 associated with sugar intake is associated with body shape, lower total body-fat percentage, and higher blood pressure. Cell Rep. *23*, 327–336.
75. Grote Beverborg, N., Verweij, N., Klip, I.T., van der Wal, H.H., Voors, A.A., van Veldhuisen, D.J., Gansevoort, R.T., Bakker, S.J.L., van der Harst, P., and van der Meer, P. (2015). Erythropoietin in the general population: reference ranges and clinical, biochemical and genetic correlates. PLoS One *10*, e0125215.
76. Benyamin, B., Esko, T., Ried, J.S., Radhakrishnan, A., Vermeulen, S.H., Traglia, M., Gögele, M., Anderson, D., Broer, L., Podmore, C., et al. (2014). Novel loci affecting iron homeostasis and their effects in individuals at risk for hemochromatosis. Nat. Commun. *5*, 4926.
77. Chen, M.-H., Raffield, L.M., Mousas, A., Sakaue, S., Huffman, J.E., Moscati, A., Trivedi, B., Jiang, T., Akbari, P., Vuckovic, D., et al. (2020). Trans-ethnic and ancestry-specific blood-cell genetics in 746, 667 individuals from 5 global populations. Cell *182*, 1198–1213.e14.
78. Tong, Z., Yang, Z., Patel, S., Chen, H., Gibbs, D., Yang, X., Hau, V.S., Kaminoh, Y., Harmon, J., Pearson, E., et al. (2008). Promoter polymorphism of the erythropoietin gene in severe diabetic eye and kidney complications. Proc. Natl. Acad. Sci. USA *105*, 6998–7003.
79. Amanzada, A., Goralczyk, A.D., Reinhardt, L., Moriconi, F., Cameron, S., and Mihm, S. (2014). Erythropoietin rs1617640 G allele associates with an attenuated rise of serum erythropoietin and a marked decline of hemoglobin in hepatitis C patients undergoing antiviral therapy. BMC Infect. Dis. *14*, 503.
80. Franklin, B.H. (2013). Erythropoietin. Cold Spring Harb. Perspect. Med. *3*, a011619.
81. Forejtnikovà, H., Vieillevoye, M., Zermati, Y., Lambert, M., Pellegrino, R.M., Guihard, S., Gaudry, M., Camaschella, C., Lacombe, C., Roetto, A., et al. (2010). Transferrin receptor 2 is a component of the erythropoietin receptor complex and is required for efficient erythropoiesis. Blood *116*, 5357–5367.
82. Regev, A., Teichmann, S.A., Lander, E.S., Amit, I., Benoist, C., Birney, E., Bodenmiller, B., Campbell, P., Carninci, P., Clatworthy, M., et al. (2017). The Human Cell Atlas. Elife *6*, e27041.
83. Fan, Y., Fu, Y.-Y., Chen, Z., Hu, Y.-Y., and Shen, J. (2016). Gene-gene interaction of erythropoietin gene polymorphisms and diabetic retinopathy in Chinese Han. Exp. Biol. Med. *241*, 1524–1530.
84. Kästner, A., Grube, S., El-Kordi, A., Stepniak, B., Friedrichs, H., Sargin, D., Schwitulla, J., Begemann, M., Giegling, I., Miskowiak, K.W., et al. (2012). Common variants of the genes encoding erythropoietin and its receptor modulate cognitive performance in schizophrenia. Mol. Med. *18*, 1029–1040.
85. Renner, W., Kaiser, M., Khuen, S., Trummer, O., Mangge, H., and Langsenlehner, T. (2020). The Erythropoetin rs1617640 Gene Polymorphism Associates with Hemoglobin Levels, Hematocrit and Red Blood Cell Count in Patients with Peripheral Arterial Disease. Genes *11*, 1305.
86. Duarte, S., Woll, P.S., Buza-Vidas, N., Chin, D.W.L., Boukarabila, H., Luís, T.C., Stenson, L., Bouriez-Jones, T., Ferry, H., Mead, A.J., et al. (2018). Canonical Notch signaling is

dispensable for adult steady-state and stress myelo-erythropoiesis. Blood *131*, 1712–1719.

87. Huang, C., Yang, D., Ye, G.W., Powell, C.A., and Guo, P. (2021). Vascular Notch Signaling in Stress Hematopoiesis. Front. Cell Dev. Biol. *8*, 606448.

88. Page, P. (2014). Beyond statistical significance: clinical interpretation of rehabilitation research literature. Int. J. Sports Phys. Ther. *9*, 726–736.

89. Mokry, L.E., Ahmad, O., Forgetta, V., Thanassoulis, G., and Richards, J.B. (2015). Mendelian randomisation applied to drug development in cardiovascular disease: a review. J. Med. Genet. *52*, 71–79.

90. Sofianopoulou, E., Kaptoge, S.K., Afzal, S., Jiang, T., Gill, D., Gundersen, T.E., Bolton, T.R., Allara, E., Arnold, M.G., Mason, A.M., and Chung, R. (2021). Estimating dose-response relationships for vitamin D with coronary heart disease, stroke, and all-cause mortality: observational and Mendelian randomisation analyses. Lancet Diabetes Endocrinol. *9*, 837–846.

91. Visscher, P.M., Wray, N.R., Zhang, Q., Sklar, P., McCarthy, M.I., Brown, M.A., and Yang, J. (2017). 10 Years of GWAS Discovery: Biology, Function, and Translation. Am. J. Hum. Genet. *101*, 5–22.

92. Pulley, J.M., Shirey-Rice, J.K., Lavieri, R.R., Jerome, R.N., Zaleski, N.M., Aronoff, D.M., Bastarache, L., Niu, X., Holroyd, K.J., Roden, D.M., et al. (2017). Accelerating Precision Drug Development and Drug Repurposing by Leveraging Human Genetics. Assay Drug Dev. Technol. *15*, 113–119.

93. Burgess, S., Butterworth, A., Malarstig, A., and Thompson, S.G. (2012). Use of Mendelian randomisation to assess potential benefit of clinical intervention. BMJ *345*, e7325.

The American Journal of Human Genetics, Volume *109*

Supplemental information

Identification and single-base gene-editing functional validation of a *cis-EPO* variant as a genetic predictor for EPO-increasing therapies

Charli E. Harlow, Josan Gandawijaya, Rosemary A. Bamford, Emily-Rose Martin, Andrew R. Wood, Peter J. van der Most, Toshiko Tanaka, Hampton L. Leonard, Amy S. Etheridge, Federico Innocenti, Robin N. Beaumont, Jessica Tyrrell, Mike A. Nalls, Eleanor M. Simonsick, Pranav S. Garimella, Eric J. Shiroma, Niek Verweij, Peter van der Meer, Ron T. Gansevoort, Harold Snieder, Paul J. Gallins, Dereje D. Jima, Fred Wright, Yi-hui Zhou, Luigi Ferrucci, Stefania Bandinelli, Dena G. Hernandez, Pim van der Harst, Vickas V. Patel, Dawn M. Waterworth, Audrey Y. Chu, Asami Oguro-Ando, and Timothy M. Frayling

Supplemental Figures

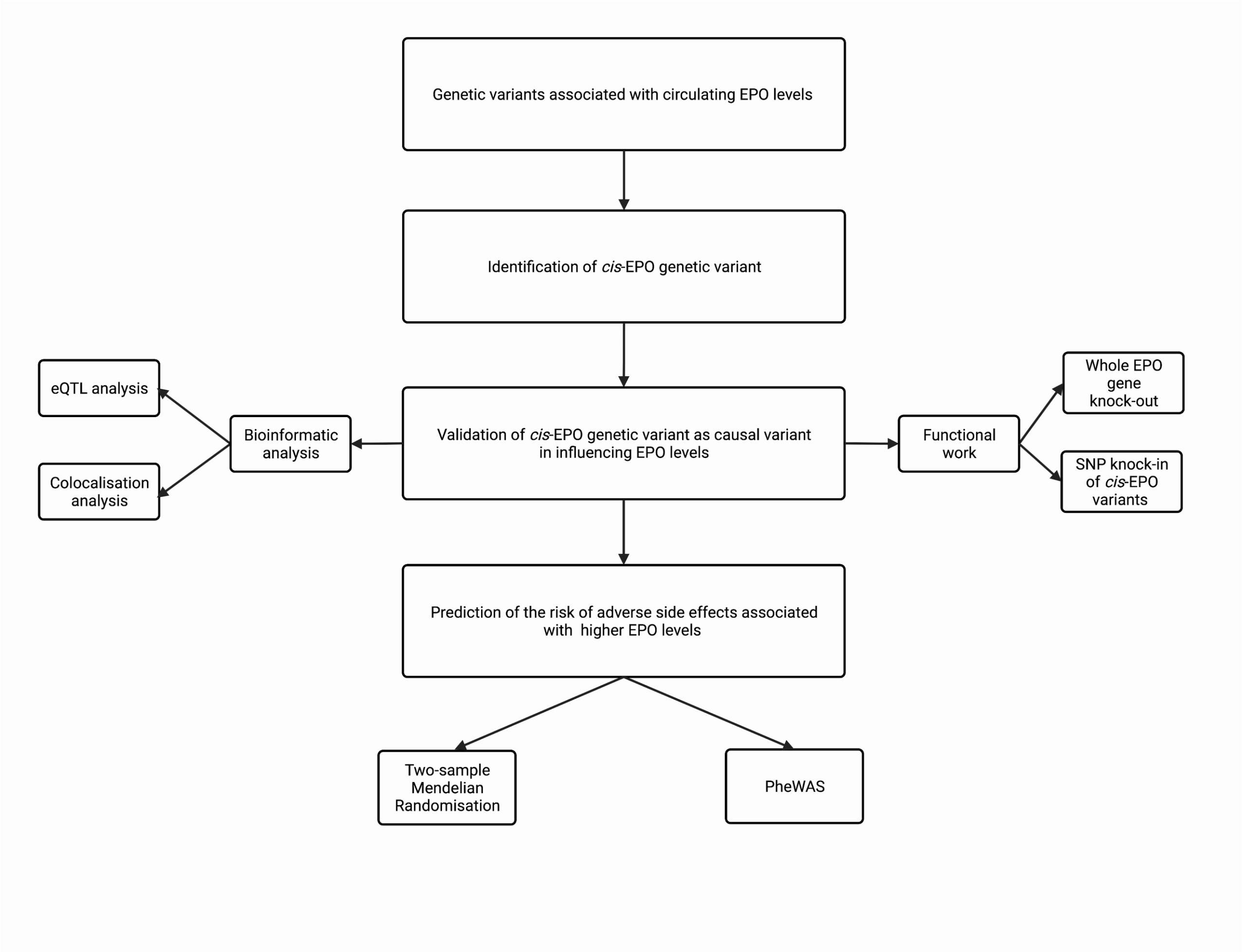

Figure S1. Study design. Using a combination of genetic studies and functional approaches, this study identified and validated a genetic variant lying in *cis* with the *EPO* gene for use as a proxy to characterize the therapeutic profile of long-term therapeutic rises in endogenous EPO levels particularly in terms of CVD risk.

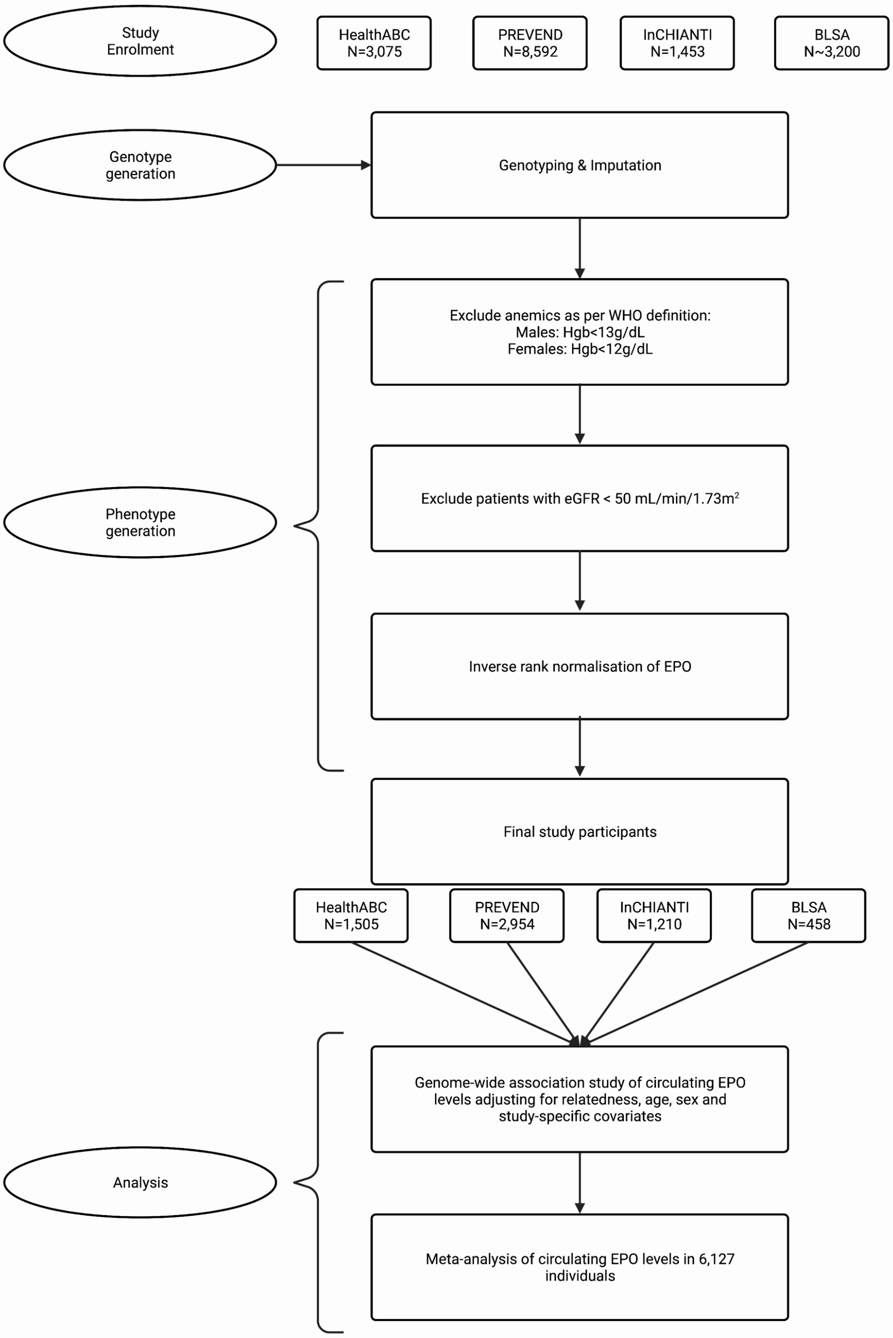

Figure S2. Analysis plan for the genome-wide association study meta-analysis of circulating EPO levels. Four independent cohorts with valid genotypic and phenotypic data were used for the meta-analysis of circulating EPO levels. The EPO phenotype was derived by excluding anemic individuals and those with an estimated glomerular filtration rate < 50 mL/min/1.73m^2. Phenotypes were then inverse rank normalized to account for the skewed distribution. GWAS was performed adjusting for age, sex, PCs and study specific covariates before a fixed-effects inverse variance-weighted meta-analysis was implemented. The final study sample size included 6,127 individuals of European and African American ancestry.

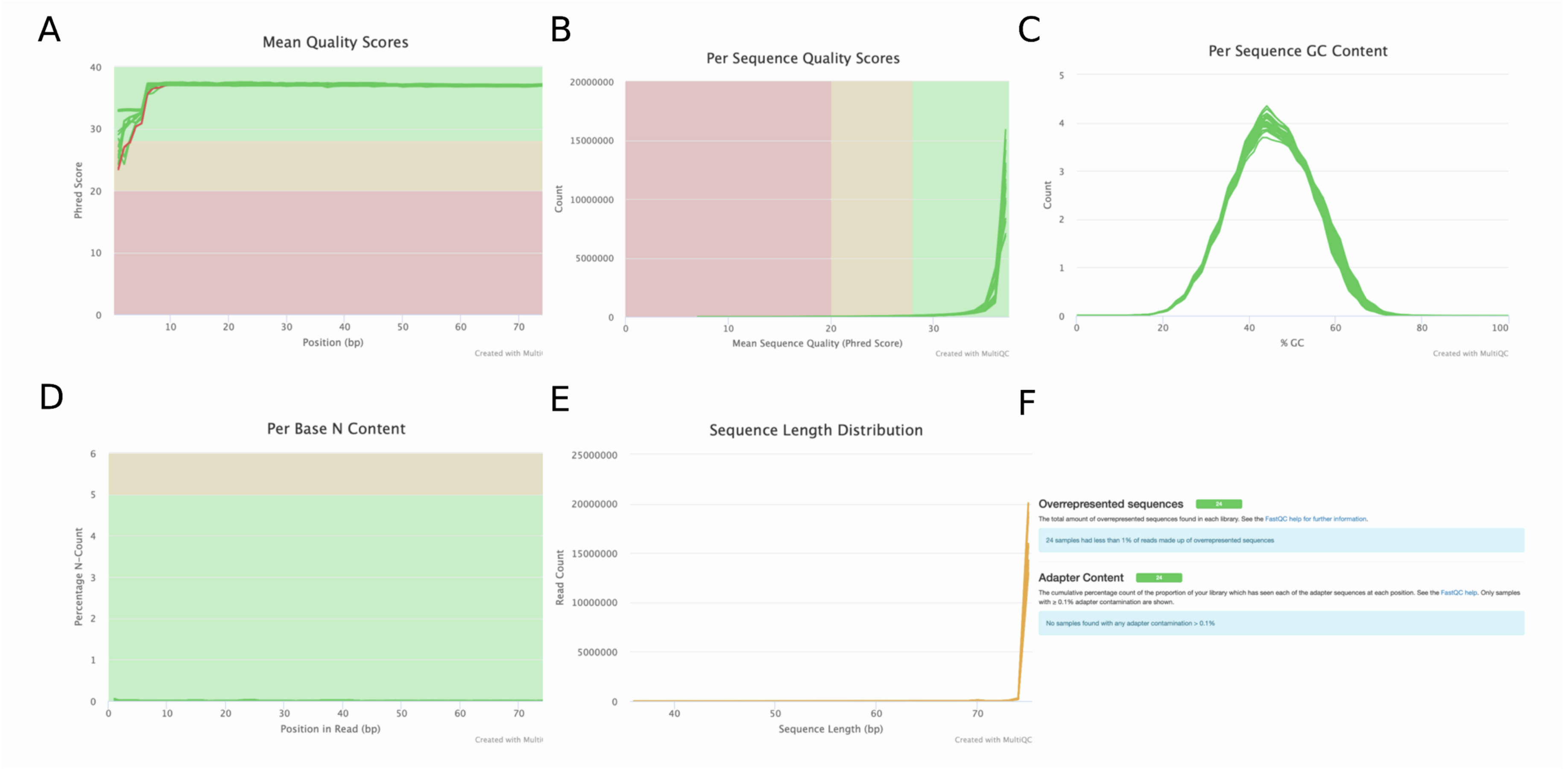

Figure S3. Quality Control checks of the RNA-seq raw reads after trimming had been performed. a) Plot of the Phred Scores for the 12 trimmed sequencing reads. All Phred scores are above 20. **b)** Per sequence quality plot. All sequencing reads had an average quality > 30. **c)** Plot showing the GC content per sequence. **d)** The number of bases read as 'N' along each sequencing read. **e)** The distribution of the sequence lengths across reads. **f)** Quality control check to see if all adapter sequences had been removed and if any sequences were over-represented. Images were produced using MultiQC.

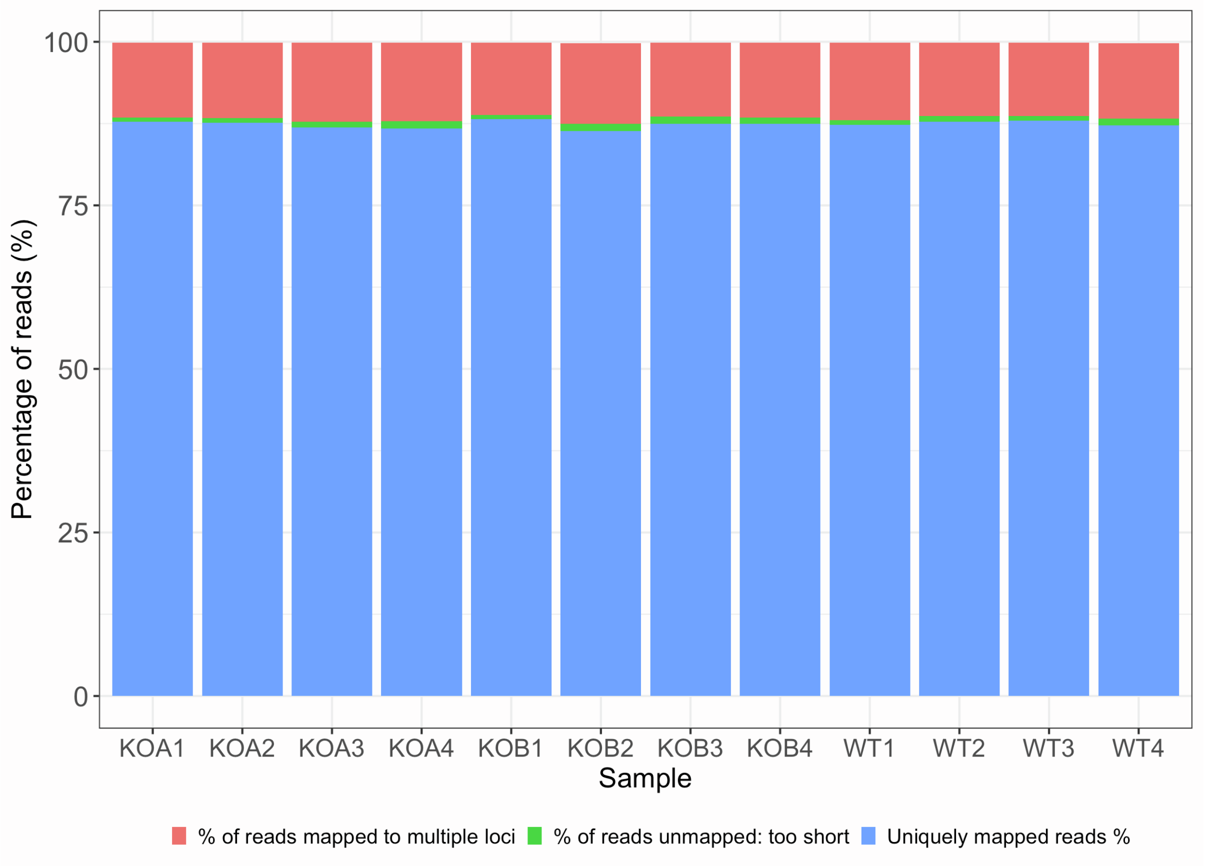

Figure S4. Alignment statistics for the RNA-seq analysis. The percentage of reads (y-axis) per sample (x-axis) aligning to either unique positions (blue), multiple loci (red) or no loci due to being too short (green) on the GRCh38/hg38 reference genome.

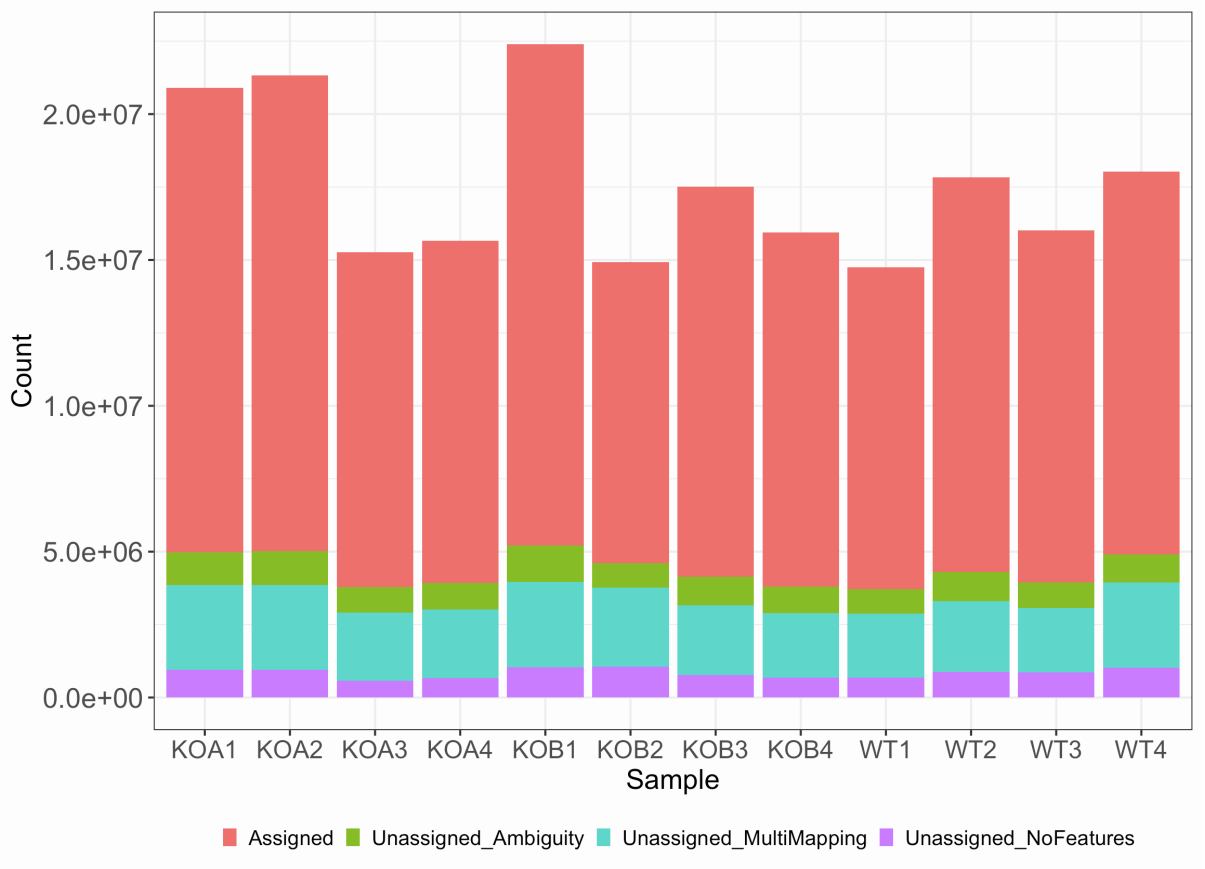

Figure S5. Gene Quantification Plot. The number of reads (y-axis) per sample (x-axis) being assigned to genomic features (exons). The red blocks represent reads that reads that were successfully assigned to a genomic feature. The green blocks represent reads unassigned to a genomic feature due to ambiguity. The turquoise blocks represent reads unassigned to a genomic feature due to multi-mapping. The purple blocks represent reads unassigned to a genomic feature due to not overlapping any genomic feature.

A

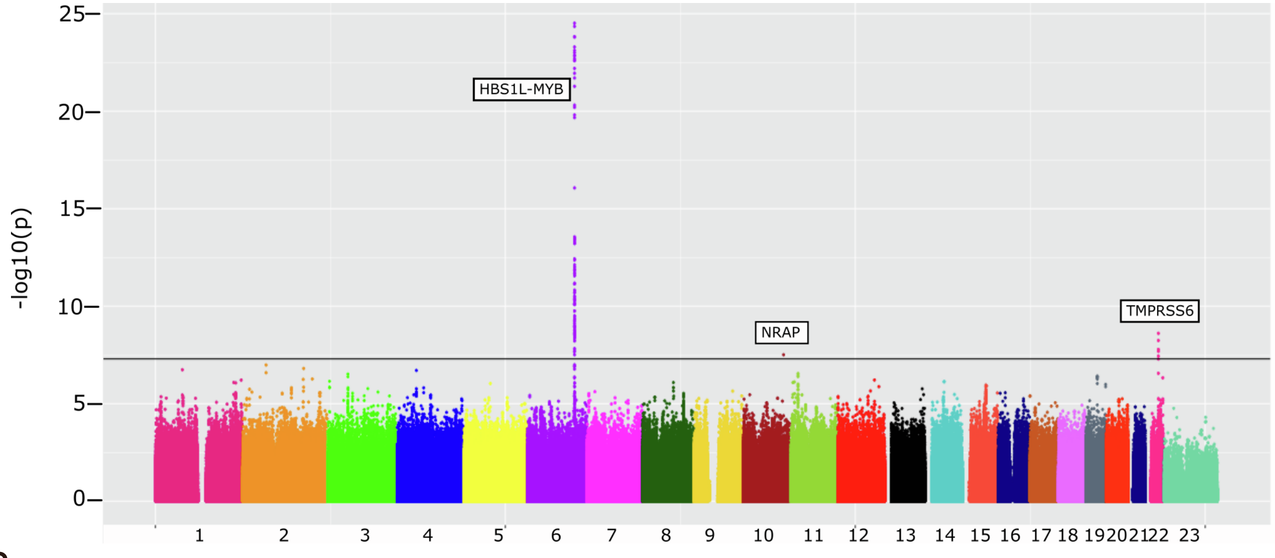

B

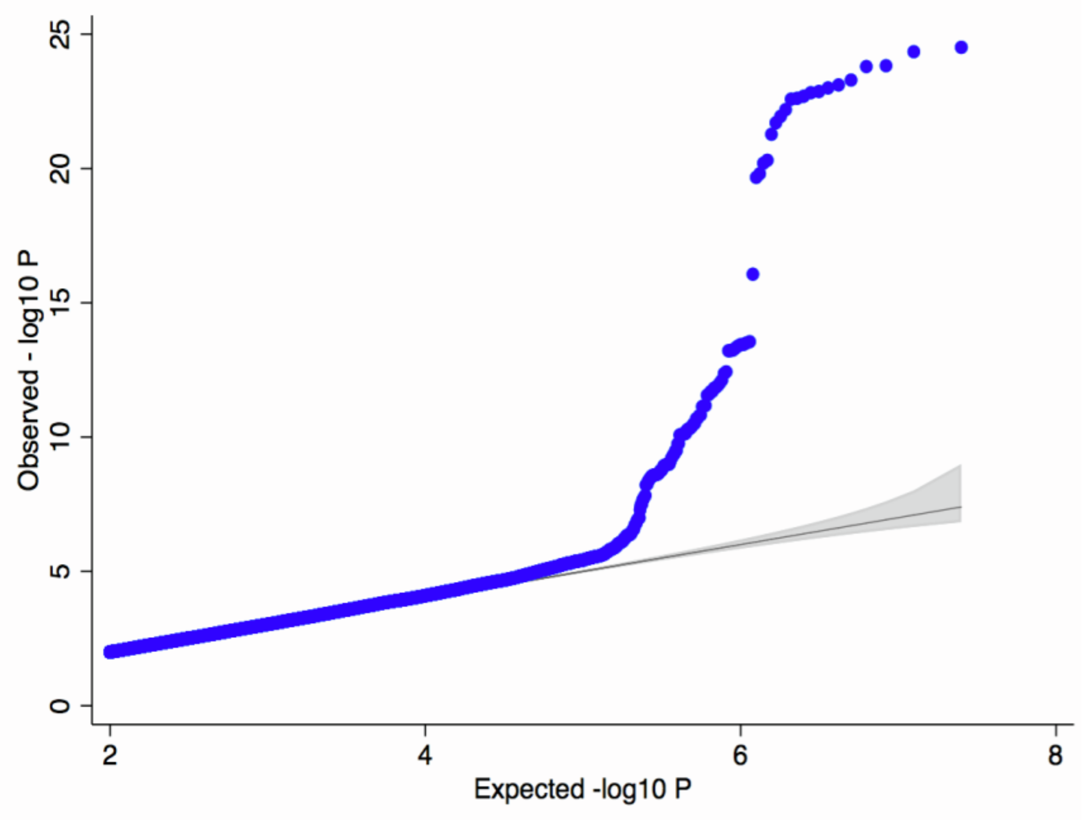

Figure S6. Genome-wide association study of endogenous EPO in 6,127 individuals. A: Manhattan Plot of results from genome-wide association study meta-analysis of circulating EPO levels in 6,127 individuals of European and African American descent. Meta-analysis was performed on genome-wide summary statistics from four independent studies using a fixed-effects inverse-variance weighted models. Black solid line indicates p value threshold for genome-wide significance ($p < 5 \times 10^{-08}$). Conditionally independent genomic loci passing genome-wide significance, as identified through conditional analysis, are labelled. Each dot represents an individual marker. Results are plotted by chromosome and position (x-axis). **B:** Quantile-Quantile plots of p values from genome-wide association study

meta-analysis in 6,127 individuals of European and African descent. The areas shaded in grey are 95% confidence bands for the black diagonal line (i.e. the expected distribution of p values). Points to the left of the diagonal line represent associations that are more significant than expected

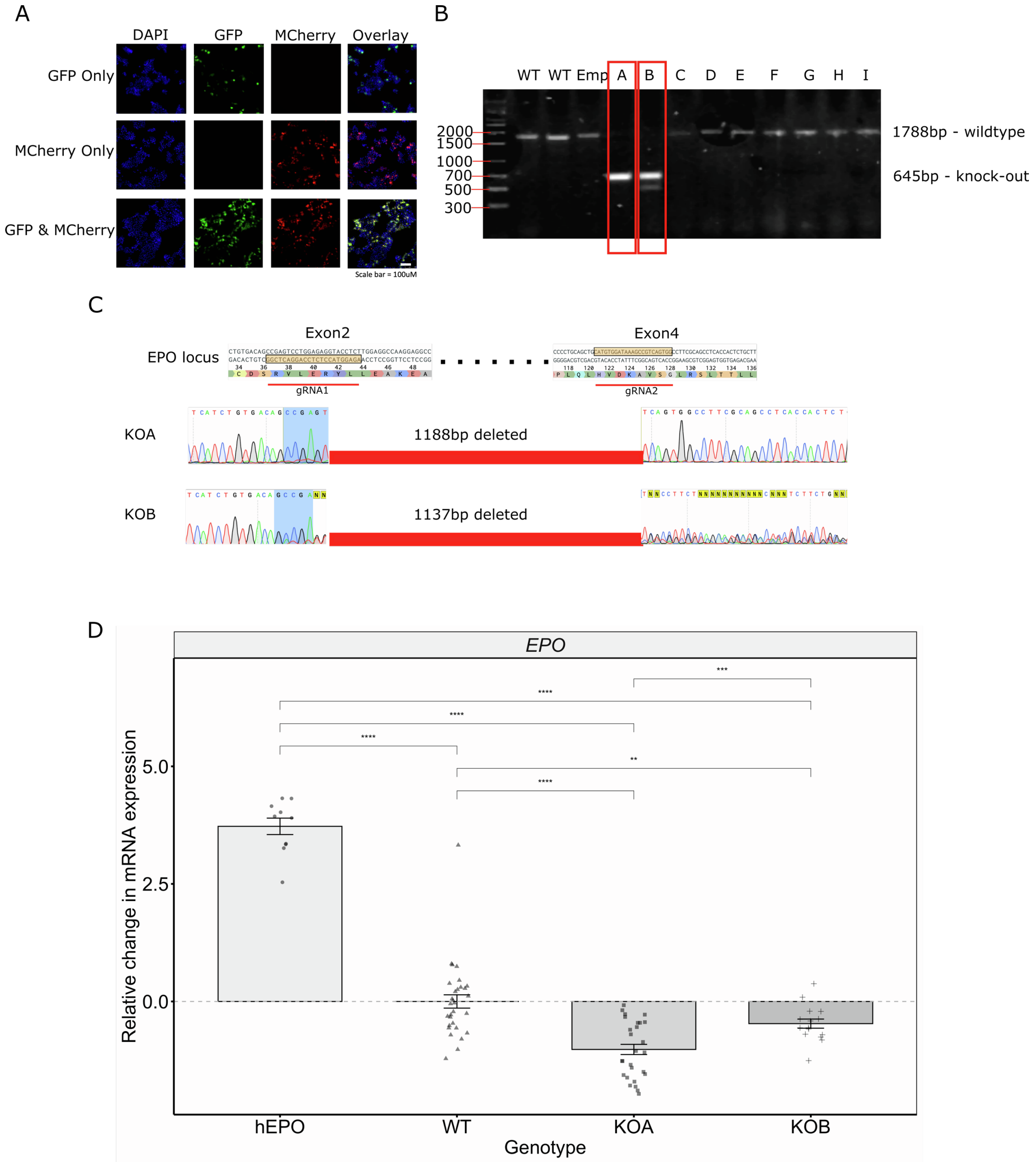

Figure S7. Establishment of whole *EPO* gene knock-out cell-lines in HEK-293 cells using CRISPR-Cas9 gene-editing technology with paired gRNA approach. **A)** Fluorescence microscopy images of HEK-293 cells to confirm successful transfection of both CRISPR-Cas9 plasmids (mCherry and GFP) containing the paired gRNAs. **B)** PCR Gel electrophoresis image screening CRISPR-Cas9/gRNA treated cell-lines for potential *EPO* knock-out. Cell-lines A and B appeared to be potential *EPO* knock-outs due to the presence of a PCR amplicon of 645 bp as opposed to 1788 bp. WT = wild-type HEK-293 cells, Emp = wild-type HEK-293 cells treated with empty CRISPR-Cas9 vectors (i.e. no gRNAs) used as a negative control. Lane 1 kb ladder. **C)** Sanger sequencing of KOA and KOB to confirm successful disruption of the *EPO* genomic sequence. The expected sequence between the two gRNAs has been deleted from the genome in both KOA and KOB confirming successful gene-editing. **D)** qRT-PCR analysis confirmed a significant reduction in *EPO* mRNA expression levels in KOA and KOB compared to WT cells. Scale bar in A represents 100μM. Data in D is shown as mean ± SEM. Paired t-test was performed. * $p \leq 0.05$, ** $p \leq 0.01$, *** $p \leq 0.001$, **** $p \leq 0.0001$.

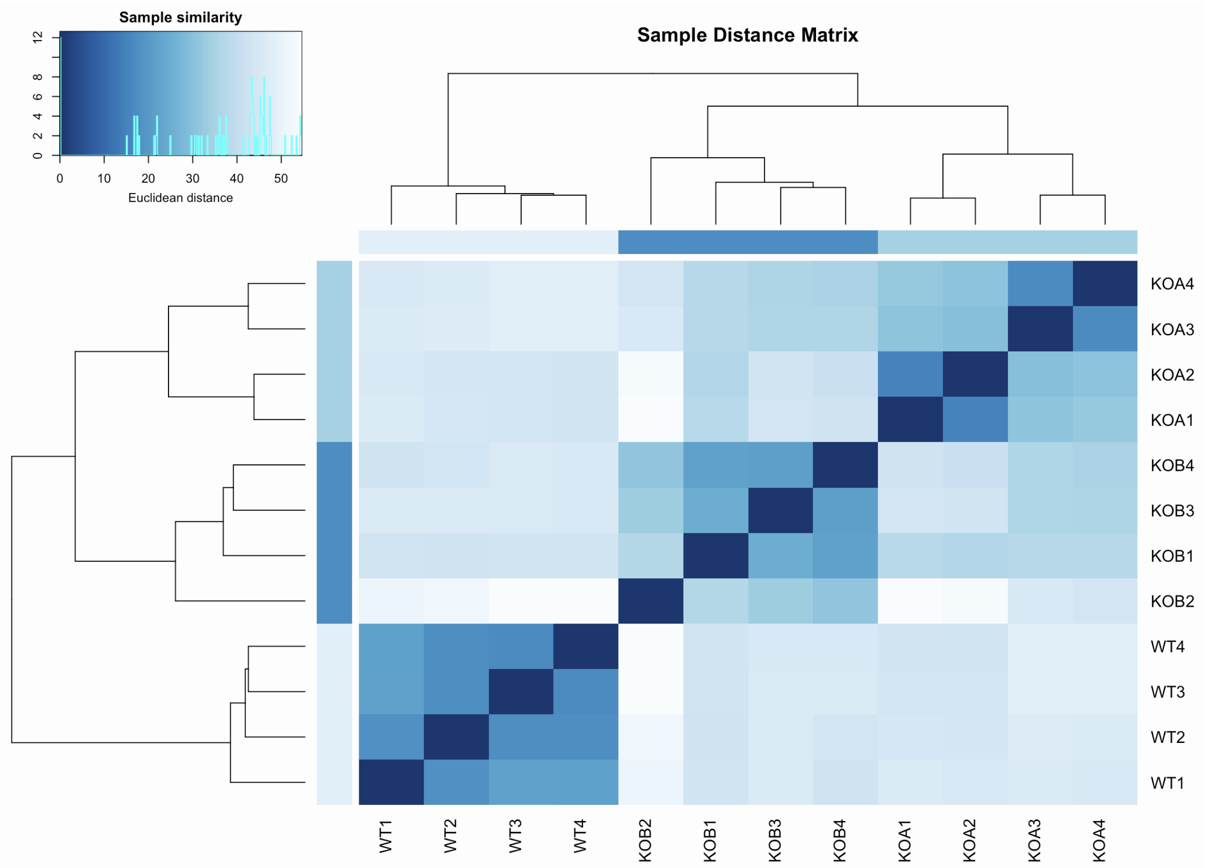

Figure S8. Distance Matrix Plot illustrating the transcriptomic profile of wild-type HEK293 cells compared to the *EPO*$^{-/-}$ cell-lines. The color scale represents the distances between samples, where the darker the blue represents the smaller the distance between samples.

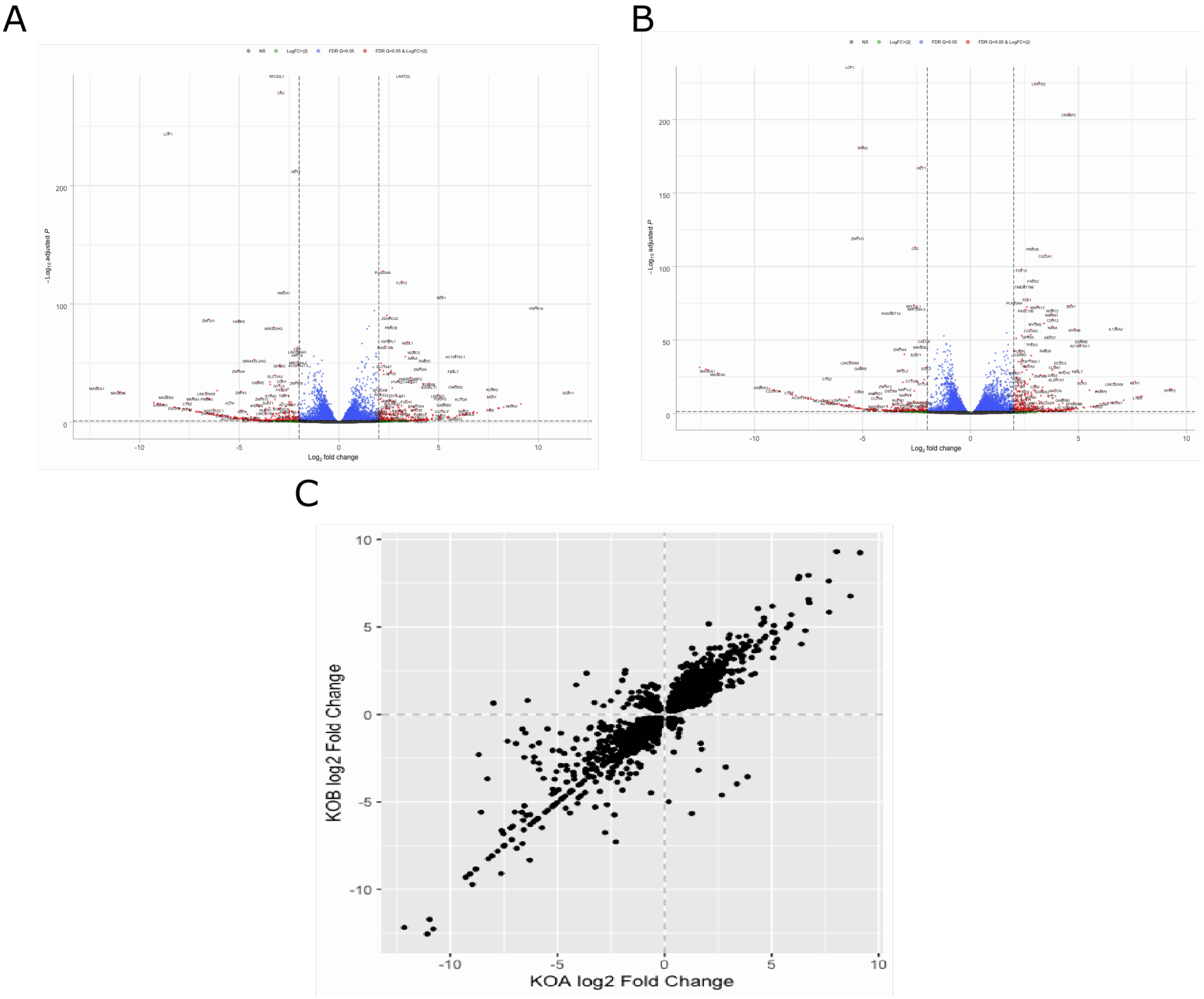

Figure S9. Differential gene expression analysis of WT HEK-293 cells versus the two *EPO*$^{-/-}$ cell-lines (KOA or KOB). A) Volcano plot of differential gene expression analysis comparing WT to KOA. **B)** Volcano plot of differential gene expression analysis comparing WT to KOB. The x-axis shows the log_2 fold-change and the y-axis represents the $-log_{10}$ adjusted p value. The vertical dashed lines represent a log_2 fold-change > |2| and the horizontal lines represent an adjusted p value ≤ 0.05. The red circles represent genes with an adjusted p value ≤ 0.05 and a log_2 fold-changes > |2|, the blue circles represent genes with an adjusted p value ≤ 0.05, the green circles represent genes with a log_2 fold-change > |2|. Black dots represent all gene that did not reach statistical significance (i.e. had a log_2 fold-change < |2| or an adjusted p > 0.05). **C)** Comparison of the log_2 fold-changes of the 3,722 overlapping DEGs (p ≤ 0.05) identified from WT vs KOA (x-axis) differential gene expression analysis and the WT vs KOB (y-axis) differential gene expression analysis. Pearson's correlation coefficient, $r^2 = 0.90$, $p < 2.2 \times 10^{-16}$. The 3,501 DEGs with consistent log_2 fold-changes were taken forward for further analysis.

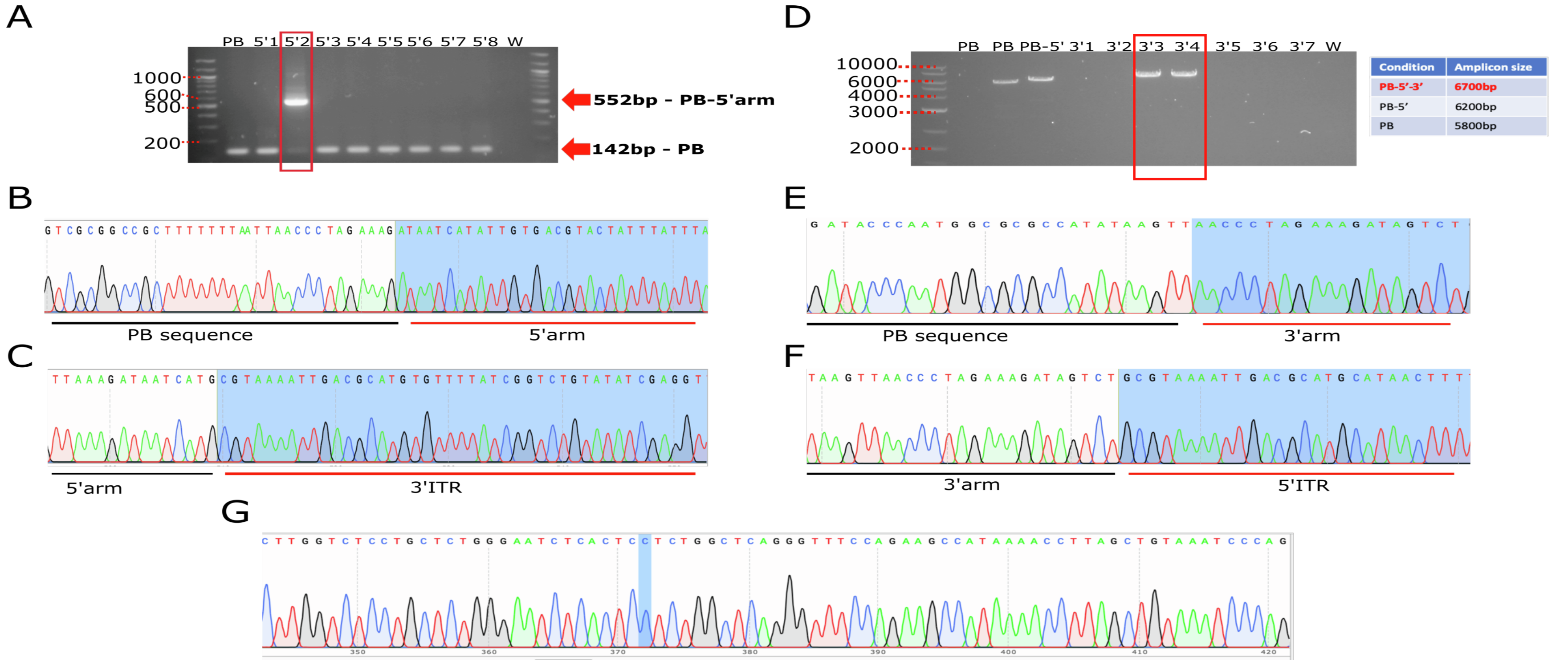

Figure S10. Cloning of the 5’ and 3’ homology arms into the *piggyBac™* plasmid. A) PCR Gel electrophoresis to screen *piggyBac™* plasmids for successful cloning of the 5’ homology arm upstream of the 3’ITR region. Plasmid 5’2 contained the 5’ homology arm inserted into *piggyBac™* plasmid. Lane 1: 100 bp ladder. **B)** Sanger sequencing to confirm successful integration of the 5’ homology arm within the correct location downstream of the plasmid sequence in the *piggyBac™* backbone plasmid. **C)** Sanger sequencing to confirm successful integration of the 5’ homology arms upstream of the 3’ITR in the *piggyBac™* backbone plasmid. **D)** Gel electrophoresis of *NsiI* digested *piggyBac™* plasmids to screen for *piggyBac™* plasmids which contain the 5’homology arm and the 3’homology arm. Digested plasmid 3’3 and 3’4 appear to have both the 5’ and 3’ homology arms. Lane 1: 1 kb ladder. **E)** Sanger sequencing to confirm successful integration of the 5’ homology arm within the correct location upstream of the *piggyBac™* plasmid sequence. **F)** Sanger sequencing to confirm successful integration of the 5’ homology arm downstream of the 5’ITR in the *piggyBac™* backbone plasmid. **G)** Sanger sequencing to confirm the desired single base gene-edit (A > C at variant rs1617640) within the 5’ homology arm. PB: empty *piggyBac™* plasmid; W: ddH_20; PB-5’: *piggyBac™* plasmid with the 5’arm inserted.

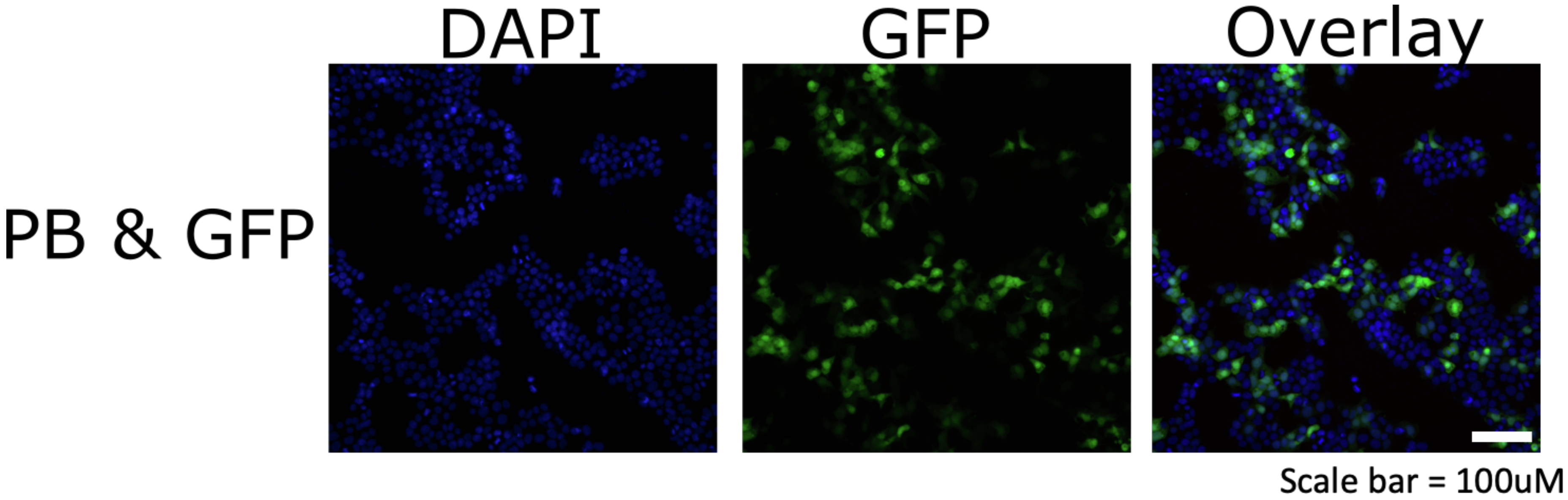

Figure S11. Fluorescent microscopy images confirming successful transfection of the CRISPR-Cas9-GFP plasmid and the *piggyBac™* plasmid into HEK-293 cells. Scale bar represents 100μM.

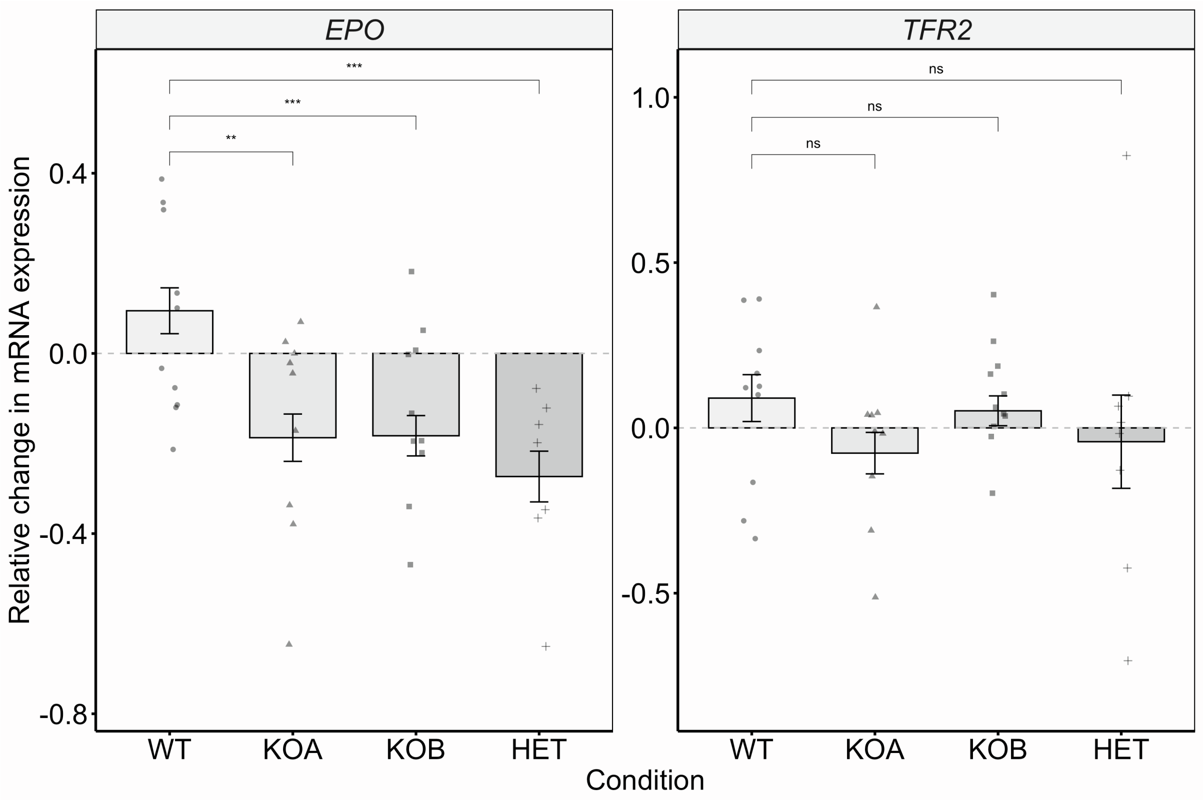

Figure S12. Investigation of the effect of disruption to the *EPO* gene on *TFR2* mRNA expression. qRT-PCR showed no difference in *TFR2* mRNA expression in *EPO*$^{-/-}$ knock-outs (KOA or KOB) or heterozygotes for the A-allele at the *cis-EPO* SNP (HET) compared to wild-type control cells homozygote for the A-allele at the *cis-SNP* (WT). Data is shown as mean ± SEM. Paired t-test was performed. * p ≤ 0.05, **p ≤ 0.01, *** p ≤ 0.001, ns=non-significant.

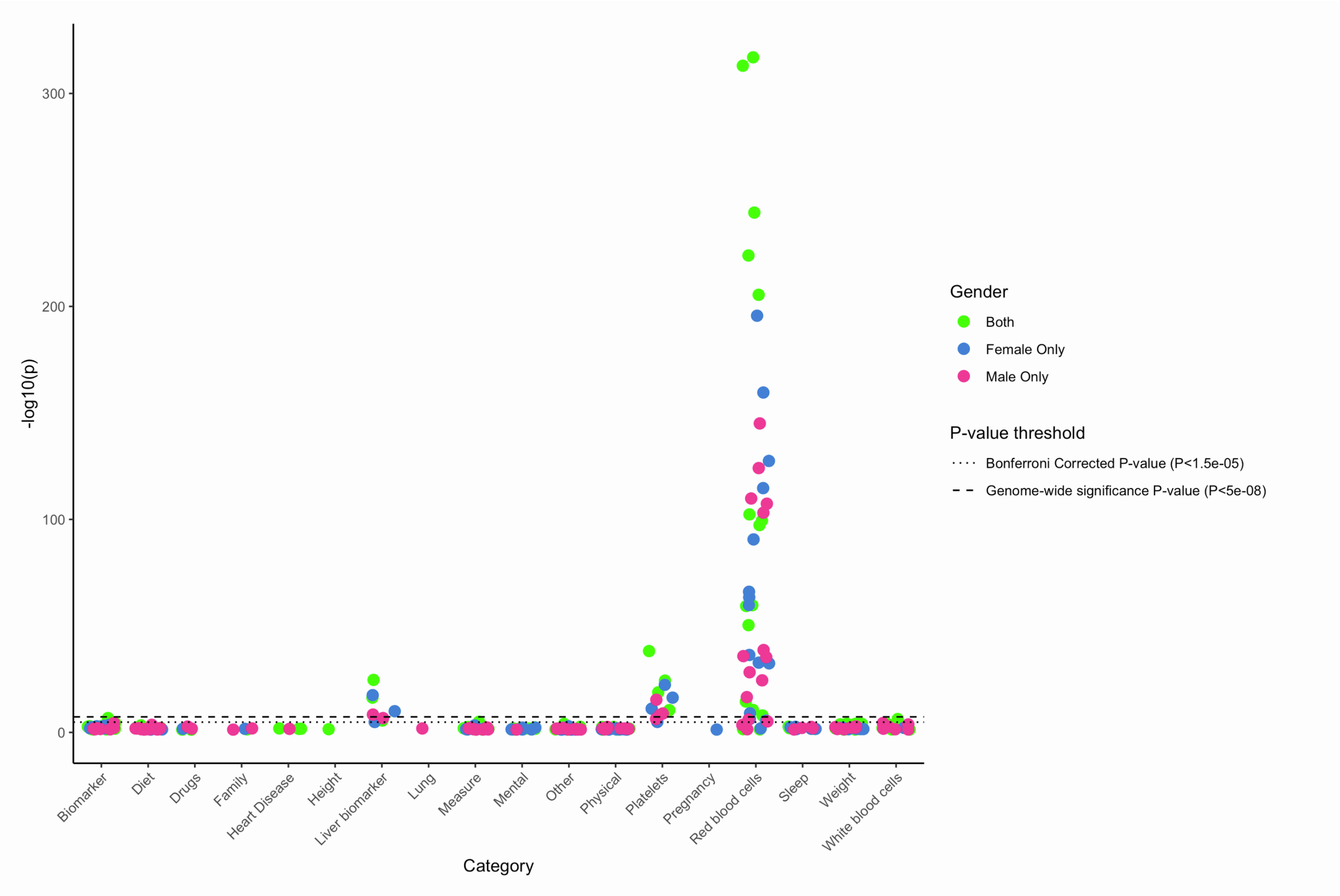

Figure S13. PheWAS of the *cis*-EPO SNP with 869 traits in up to 451,099 individuals from UK Biobank. Plot represents the -$\log_{10}$ p values (y-axis) for all traits passing a p value threshold of 0.05. Analysis was performed in both males and females combined (green dots), females only (blue dots) and males only (pink dots). The dotted line highlights associations passing a Bonferroni corrected p value < 1.5 x 10^{-05} and the dashed line highlights associations passing genome-wide significance p value < 5 x 10^{-08}. Traits have been clumped together into categories which are represented on the x-axis.

Supplemental Tables

Table S1. Conditionally independent SNPs associated with (at p < *5E-08)* circulating EPO levels in meta-analysis of 6,127 individuals of European and African American descent.

Lead SNP	Chromosome	Position	Genomic Locus	Effect allele	EAF	Effect size	SE	p value	Sample size
rs4895441	6	135426573	HBS1L	A	0.74	-0.24	0.021	1.45E-30	6127
rs112631630	10	115407228	NRAP	A	0.996	3.598	0.65	3.09E-08	536
rs855791	22	37462936	TMPRSS6	A	0.42	0.113	0.019	2.47E-09	6529

Table S2. Study characteristics of the four independent cohorts included in EPO meta-analysis.

Cohort	Sample size	% Men	Mean Age/years (SD)	Mean EPO/ IU/L (SD)	Mean hemoglobin/ g/dL (SD)	Mean eGFR/ mL/min/1.73m^2 (SD)	Software GWAS implemented in	Covariates adjusted for	Other sample exclusion criteria based on GWAS data
InCHIANTI	1210	44.63	66.7(15.3)	9.7 (5.1)	14.1 (1.1)	75.8 (16.0)	GEMMA 0.94.1	Age and Sex	Genotype or phenotype missing data
PREVEND	2954	51.76	53.69(11.92)	9.03 (14.94)	13.76(1.23)	80.95 (13.95)	SNPtest v2.5.4	Age, Sex, 10 Pcs	Genotype or phenotype missing data, sex mismatch, <95% call rate, PC outliers.
HealthABC Europeans	969	51.7	73.678 (2.772)	12.937 (6.467)	14.161 (1.081)	78.458 (15.953)	Rvtests 2.1.0	Age, Sex, Study site, and PCs	Excess heterozygosity, missingness > 5%, sex mismatch, population

									outliers, and related individuals were excluded by a grm cut-off of 0.125 (no closer than cousin)
Health ABC African Americans	536	41.2	73.254 (2.858)	13.626 (1.014)	13.626 (1.014)	88.332 (20.832)	Rvtests 2.1.0	Age, Sex, Study site, and PCs	Excess heterozygosity, missingness > 5%, sex mismatch, population outliers, and related individuals were excluded by a grm cut-off of 0.125 (no closer than cousin)
BLSA	458	50	69.1 (13.6)	15.2 (1.48)	14.0 (1.1)	72.1 (13.8)	GEMMA 0.94.1	Age, sex and PCs	Phenotype or GWAS missing data

Table S3. Association between rs1617640 and EPO and TFR2 expression in human liver. The data was coded such that a negative beta (tmeta) means that as the number of minor alleles (C-alleles) increases there is a decrease in *EPO* or *TFR2* expression.

**Dataset**	**Sample Size**	**Expression**	**Genotyping**	**PMID**	***EPO***			***TFR2***		
					**Beta**	**SE**	**p value**	**Beta**	**SE**	**p value**
Dataset1	161	Agilent-014850 Whole Human Genome. 4x44K gene expression (NCBI GEO accession: GSE25935)	Illumina Human610-Quad v1.0 BeadChip (NCBI GEO accession: GSE26105)	21637794	0.0592	0.1008	0.558	0.0827	**1.56 E-13**	0.3036
		4x44K gene expression	(NCBI GEO accession: GSE26105)							
		(NCBI GEO accession: GSE25935)								
Dataset2	145	Illumina Human Whole Genome-6 v2.0 (NCBI GEO accession: GSE32504)	Illumina HumanHap300-Duo v2.0 Genotyping (NCBI GEO accession: GSE39036)	18462017	0.1048	0.0989	0.2911	0.2653	0.0729	3.98 E-4
		(NCBI GEO accession: GSE32504)	(NCBI GEO accession: GSE39036)							

Dataset3	555	Agilent Technologies (NCBI GEO accession: GSE9588)	HumanHap 650Y	21602305	0.2842	0.0492	1.35 E-8	0.246	0.0367	5.43 E-11
		(NCBI GEO accession: GSE9588)								
**Meta-analysis**	**861**				**T_{mets} = 5.39**	**0.1488**	**6.86 E-8**	**T_{mets} = 7.38**	**0.1488**	**1.56 E-13**

Table S4. Association between rs1617640 and *EPO* and *TFR2* expression in human kidneys. The data was coded such that a negative beta (tmeta) means that as the number of minor alleles (C-alleles) increases there is a decrease in *EPO* or *TFR2* expression.

**Dataset**	**Sample size**	**Tissue**	***EPO***				***TFR2***			
			**T-statistic**	**Beta**	**SE**	**p value**	**T-statistic**	**Beta**	**SE**	**p value**
Kidney TransplantLines [2]	286	Kidney	0.117	0.157	1.338	0.907	1.358	1.333	0.981	0.177

Table S5. List of primers used in qRT-PCR.

Gene	Primer name	Sequence (5’-3’)
EPO	EPO-qpcr_fwd	CCTTCGCAGCCTCACCACT
EPO	EPO-qpcr_rev	TGTACAGCTTCAGCTTTCCCC
GAPDH	GADPH_qpcr_fwd	TCCTCTGACTTCAACAGCGAC
GAPDH	GADPH_qpcr_rev	GCTGTAGCCAAATTCGTTGTCA
IFITM2	IFITM2_qpcr_fwd	TTCATGAACACCTGCTGCCT
IFITM2	IFITM2_qpcr_rev	AGATGTTCAGGCACTTGGCG
LRATD2	LRATD2_qpcr_fwd	GCCGAGCCTACACCTTCAAA
LRATD2	LRATD2_qpcr_rev	CGAAACCAACTCCAGGGTCA
MLH1	MLH1_qpcr_fwd	GAAGTTATCCAGCGGCCAG
MLH1	MLH1_qpcr_rev	TGAATCAACTTCAGGCCTCC
ZNF331	ZNF331_qpcr_fwd	GGTCTCACTGGATTTGGAGT
ZNF331	ZNF331_qpcr_rev	AGCGTACCTTCACATATCCAG
HEY1	HEY1_qpcr_fwd	TGCGGACGAGAATGGAAACT
HEY1	HEY1_qpcr_rev	TCGTCGGCGCTTCTCAATTA
HEY2	HEY2_qpcr_fwd	GCAACAGGGGGTAAAGGCTA
HEY2	HEY2_qpcr_rev	CTTCCACGGAGCTCAGGTAC
HES1	HES1_qpcr_fwd	AAGAAAGATAGCTCGCGGCA
HES1	HES1_qpcr_rev	TACTTCCCCAGCACACTTGG
NOTCH1	NOTCH1_qpcr_fwd	CGCACAAGGTGTCTTCCAG
NOTCH1	NOTCH1_qpcr_rev	AGGATCAGTGGCGTCGTG
PARP9	PARP9_qpcr_fwd	GGCAAAGAGGTCCAAGATGCTG
PARP9	PARP9_qpcr_rev	GCCTCATACATCTCTTCCACGT
DTX3L	DTX3L_qpcr_fwd	AGAGCTCTAAGTCCTCGGGC

DTX3L	DTX3L_qpcr_rev	ACTCTCTCCTTAGCTGCCCT
VEGFR3	VEGFR3_qpcr_fwd	TGCACGAGGTACATGCCAAC
VEGFR3	VEGFR3_qpcr_rev	GCTGCTCAAAGTCTCTCACGA
TFR2	TFR2_qpcr_fwd	TTTCCACCAGGGCAGACTCT
TFR2	TFR2_qpcr_rev	TGGTTTGCCTGATGGTGTCC
POLR2A	POLR2A_qpcr_fwd	CCATCAAGAGAGTCCAGTTCG
POLR2A	POLR2A_qpcr_rev	ACCCTCCGTCACAGACATTC
PPIA	PPIA_qpcr_fwd	TTCATCTGCACTGCCAAGAC
PPIA	PPIA_qpcr_rev	TCGAGTTGTCCACAGTCAGC
UBC	UBC_qpcr_fwd	ATTTGGGTCGCGGTTCTTG
UBC	UBC_qpcr_rev	TGCCTTGACATTCTCGATGGT

Table S6. Comprehensive gene stability of the tested housekeeping genes for use as a reference gene in qRT-PCR.

Genes	Geomean of ranking values	Stability
GAPDH	1.41	Most stable
Pol2Rα+GAPDH	1.57	
GAPDH+UBC	2.63	
Pol2Rα	3.66	
UBC	4.73	
Pol2Rα+GAPDH+UBC	6	Least stable

Table S7. List of primers used in the establishment of the rs1617640 knock-in model using CRISPR-Cas9 and the *piggyBac* system.

Primer Name	Function of primer	Sequence (5'-3')
F1	Genotyping	CTGGTAGTTTCACCACACCCA
R1	Genotyping	TTGGGCGGAGACTCAGAGAT
epo_snp-forward	Genotyping	CTGAATGGGATAGGCTGGTAGT
PB1	Genotyping	CGTCAATTTTACGCATGATTATCTTTAAC
F2	Genotyping	TGAGCCACCACACCTGACTA
R2	Genotyping	TTCTTCCTCCCCACCTCACT
PB-F	Detect transposon re-integration	GGCATAGTATATCGGCATAG
PB-R	Detect transposon re-integration	GTTAGAAGACTTCCTCTGC
M13_F	Insertion of homology arm into piggyBac plasmid	TGTAAAACGACGGCCAGT
3ITR_R	Insertion of homology arm into piggyBac plasmid	CGTCAATTTTACGCATGATTATCTTTAAC
5ITR_F	Insertion of homology arm into piggyBac plasmid	GCGACGGATTCGCGCTATTTAGAAAG
M13_R	Insertion of homology arm into piggyBac plasmid	CAGGAAACAGCTATGACCATG
5arm_seq	Sequencing of 5' homology arm & 3' ITR	AGACTGCCTTGGGAAAAG
3arm_seq	Sequencing of 3' homology arm & 5'ITR	GCATTCTAGTTGTGGTTTGTCC

Table S8. rs1617640-outcome association statistics. All effects have been aligned to the EPO-increasing allele (A).

Disease Outcomes									
**Outcome**	**Study**	**A1**	**A2**	**A1 freq**	**OR**	**Lower 95%**	**Upper 95%**	**p value**	**N (Cases/Controls)**
CAD	UK Biobank	A	C	0.6	1.001	0.986	1.016	0.82	37741 / 318892
CAD	Nikpey *et al.* (2015)	A	C	0.59	1.005	0.985	1.025	0.643	60801 / 123504
	**Meta-analysis**				**1.002**	**0.99**	**1.01**	**0.72**	**98542 / 442396**
MI	UK Biobank	A	C	0.6	0.99	0.964	1.018	0.48	10590 / 440509
MI	Nikpay *et al.* (2015)	A	C	0.57	1.004	0.983	1.026	0.706	42561 / 123504
	**Meta-analysis**				**0.999**	**0.98**	**1.02**	**0.889**	**53151 / 564013**
Stroke	UK Biobank	A	C	0.6	0.963	0.935	0.993	0.014	9092 / 346423
Stroke	Malik *et al.* (2018)	A	C	0.6	1.01	0.993	1.031	0.344	40585 / 406111
	**Meta-analysis**				**0.995**	**0.98**	**1.01**	**0.553**	**49677 / 752534**
**Continuous Outcomes**									
**Outcome**	**Study**	**A1**	**A2**	**A1 freq**	**Beta (95% CI)**	**Lower 95%**	**Upper 95%**	**p value**	**N**

SBP	UK Biobank	A	C	0.6	0.04	-0.05	0.12	0.8	450075
SBP	Wain *et al.* (2017)	A	C	0.59	0.024	-0.11	0.16	0.725	228245
	**Meta-analysis**				**0.03**	**-0.04**	**0.11**	**0.38**	**678320**
DBP	UK Biobank	A	C	0.6	-0.07	-1.20E-01	-0.02	1.00E-05	449322
DBP	Wain *et al.* (2017)	A	C	0.59	-0.03	-0.11	0.05	0.469	228245
	**Meta-analysis**				**-0.06**	**-0.1**	**-0.02**	**0.006**	**677567**
Heart rate	UK Biobank	A	C	0.6	-0.07	-0.13	-0.02	0.019	423846
Heart rate	den Hoed *et al.* (2013)	A	C	0.59	-0.021	-0.13	0.09	0.703	90849
	**Meta-analysis**				**-0.06**	**-0.11**	**-0.02**	**0.01**	**514695**

Table S9. Genome-wide significant association of rs4895441 and rs855791, two of the three lead genetic variants identified in the EPO meta-analysis, with other phenotypes in up to 451,099 UK Biobank unrelated, European individuals. Effect estimates are aligned to the EPO-increasing allele.

Genetic variant	Phenotype	Gender	Beta	SE	p value
**rs4895441**	Erythropoietin	Combined	0.218	0.021	1.45E-30
	Corpuscular hemoglobin	Combined	0.179	0.002	4.3E-1635
	Corpuscular Volume	Combined	0.157	0.002	6.2E-1353
	Mean corpuscular volume	Combined	0.157	0.002	6.7E-1270
	Red blood cell count	Combined	-0.139	0.002	2.3E-1224
	Mean corpuscular volume (anemics excluded)	Combined	0.163	0.002	2.7E-1148
	Platelet crit	Combined	0.12	0.002	5.4E-790
	Platelet Count	Combined	0.107	0.002	1.8E-635
	Fibrosis-4 Score	Combined	-0.079	0.002	1.9E-399
	Red blood cell distribution width	Combined	-0.092	0.002	1.3E-386
	Red blood cell distribution width (anemics excluded)	Combined	-0.098	0.002	4.5E-378
	Non-alcoholic fatty liver disease fibrosis score	Combined	-0.085	0.002	2.6E-367
	Corpuscular hemoglobin concentration	Combined	0.075	0.002	4.70E-261
	Sphered cell volume	Combined	0.07	0.002	7.60E-257
	Hematocrit percentage	Combined	-0.062	0.002	5.30E-255
	Non-alcoholic fatty liver disease fibrosis score	Female	-0.093	0.003	4.00E-220
	fibrosis-4 Score	Female	-0.082	0.003	1.30E-214
	Reticulocyte Volume	Combined	0.065	0.002	3.60E-209
	fibrosis-4 Score	Male	-0.078	0.003	1.30E-171
	Non-alcoholic fatty liver disease fibrosis score	Male	-0.077	0.003	3.00E-141

	Hemoglobin Concentration	Combined	-0.038	0.002	1.10E-103
	Reticulocyte Percentage	Combined	0.046	0.002	1.50E-101
	Eosinophil Count	Combined	-0.042	0.002	2.40E-83
	High Light Scatter reticulocyte percentage	Combined	0.041	0.002	8.40E-81
	Glycated hemoglobin	Combined	-0.036	0.002	3.60E-66
	Lymphocyte Count	Combined	-0.034	0.002	1.00E-52
	Monocyte Count	Combined	-0.031	0.002	9.70E-51
	Neutrophil Count	Combined	-0.03	0.002	1.40E-42
	Eosinophil Percentage	Combined	-0.029	0.002	2.10E-39
	Glycated hemoglobin	Female	-0.035	0.003	2.10E-35
	Glycated hemoglobin	Male	-0.037	0.003	2.50E-31
	Cholesterol corrected for statin use	Combined	-0.024	0.002	7.10E-30
	Low density lipoprotein corrected for statin use	Combined	-0.024	0.002	7.60E-30
	Cholesterol corrected for statin use	Male	-0.028	0.003	1.30E-19
	Platelet distribution width	Combined	0.018	0.002	2.60E-19
	Aspartate Aminotransferase	Combined	-0.019	0.002	3.40E-19
	Low density lipoprotein corrected for statin use	Male	-0.027	0.003	4.30E-18
	Albumin	Combined	0.02	0.002	4.00E-17
	Cholesterol	Combined	-0.017	0.002	9.10E-15
	High Light Scatter Reticulocyte Count	Combined	0.016	0.002	2.20E-14
	Low density lipoprotein corrected for statin use	Female	-0.021	0.003	2.70E-14
	Cholesterol corrected for statin use	Female	-0.021	0.003	6.40E-14
	Low density lipoprotein	Combined	-0.015	0.002	2.40E-12
	Aspartate Aminotransferase	Female	-0.021	0.003	7.70E-12
	Immature reticulocyte	Combined	0.014	0.002	6.40E-11
	Microalbumin	Combined	0.013	0.002	1.30E-10
	Reticulocyte Count	Combined	0.013	0.002	1.60E-10

	Albumin	Male	0.022	0.004	4.60E-10
	Albumin	Female	0.019	0.003	2.20E-09
	Apolipoprotein B	Combined	-0.013	0.002	2.40E-09
	Microalbumin	Female	0.019	0.003	3.25E-03
	Cholesterol levels	Male	-0.019	0.003	3.47E-03
	Aspartate Aminotransferase	Male	-0.019	0.003	3.49E-03
	High Density Lipoprotein	Male	-0.018	0.003	3.45E-03
**rs855791**	EPO	Combined	0.113	0.019	2.47E-09
	Corpuscular Hemoglobin	Combined	-0.151	0.002	3.6E-1399
	Corpuscular Volume	Combined	-0.129	0.002	3.0E-1102
	Mean corpuscular volume	Combined	-0.13	0.002	9.0E-1038
	Mean corpuscular volume (anemics excluded)	Combined	-0.133	0.002	2.5E-932
	Red blood cell distribution width (anemics excluded)	Combined	0.113	0.002	1.4E-480
	Hemoglobin concentration	Combined	-0.073	0.002	3.5E-446
	Red blood cell distribution width	Combined	0.113	0.002	1.0E-416
	Corpuscular Hemoglobin concentration	Combined	-0.067	0.002	9.20E-251
	Glycated hemoglobin levels	Combined	0.064	0.002	2.00E-244
	Hematocrit Percentage	Combined	-0.053	0.002	2.10E-224
	Sphered cell volume	Combined	-0.055	0.002	1.10E-193
	Glycated hemoglobin levels	Female	0.062	0.003	8.10E-129
	Glycated hemoglobin levels	Male	0.065	0.003	7.10E-111
	Reticulocyte Percentage	Combined	-0.04	0.002	6.60E-90
	Reticulocyte Count	Combined	-0.035	0.002	4.70E-72
	Total Bilirubin	Combined	-0.029	0.002	9.10E-67
	Platelet Count	Combined	0.027	0.002	5.00E-52
	Platelet crit	Combined	0.027	0.002	7.30E-52

	Fibrosis-4 score	Combined	-0.022	0.002	1.60E-38
	High light scatter reticulocyte percentage	Combined	-0.025	0.002	5.60E-37
	Total Bilirubin	Female	-0.031	0.003	1.40E-35
	Total Bilirubin	Male	-0.032	0.003	8.80E-34
	High light scatter reticulocyte count	Combined	-0.022	0.002	1.80E-28
	Non-alcoholic fatty liver disease fibrosis score	Combined	-0.021	0.002	3.40E-28
	Platelet distribution width	Combined	-0.019	0.002	8.90E-24
	Direct Bilirubin	Combined	-0.019	0.002	4.80E-23
	Red blood cell count	Combined	0.016	0.002	2.20E-22
	Fibrosis-4 score	Female	-0.024	0.002	4.20E-22
	Non-alcoholic fatty liver disease fibrosis score	Female	-0.025	0.003	1.40E-19
	Direct Bilirubin	Male	-0.023	0.003	2.20E-16
	Fibrosis-4 score	Male	-0.020	0.003	2.80E-15
	Immature reticulocytes	Combined	0.015	0.002	5.20E-13
	Non-alcoholic fatty liver disease fibrosis score	Male	-0.018	0.003	1.10E-10
	Phosphate levels	Combined	0.012	0.002	1.20E-08
	Direct Bilirubin	Female	-0.014	0.003	4.00E-08

Table S10. ***See separate excel sheet.***

Table S11. ***See separate excel sheet.***

Table S12. Two-sample MR, using rs1617640 as an instrumental variable and a meta-analysis of UK Biobank GWAS and previously published, publicly available GWAS, to assess the genetic association between genetically predicted therapeutically altered endogenous EPO levels and risk of CVD or clinical markers for CVD risk factors.

Exposure	Outcome	Odds ratio	Lower 95%	Upper 95%	p value	Number of Cases	Number of Controls
EPO	CAD	1.03	0.85	1.25	0.72	98542	442396
EPO	Stroke	0.92	0.70	1.21	0.55	49677	752534
EPO	MI	0.98	0.75	1.29	0.89	53151	564013
	**Risk Factors**	**Effect estimate**	**Lower 95%**	**Upper 95%**	**p value**	**Total Sample size**	
EPO	SBP	0.53	-0.65	1.71	0.38	586080	
EPO	DBP	-0.98	-1.67	-0.29	0.0057	585325	
EPO	Heart rate	-0.996	-1.74	-0.25	0.0097	514706	

Table S13. Rescaling the genetic estimates of EPO levels on adverse cardiovascular risk to the PHI-induced effect on EPO levels (obtained from a recent RCT (Meadowcroft et al., 2019)).

Disease				
**Exposure**	**Outcome**	**Odds ratio**	**Lower 95%**	**Upper 95%**
EPO	CAD	1.0141892	0.9315804	1.07268792
EPO	MI	0.99296952	0.86721782	1.14986417
EPO	Stroke	0.96527007	0.86721782	1.07268792
**Risk Factors**				
**Exposure**	**Outcome**	**Effect estimate**	**Lower 95%**	**Upper 95%**
EPO	SBP	0.21155347	-0.2820713	0.77569607
EPO	DBP	-0.4231069	-0.7051782	-0.1410356
EPO	Heart rate	-0.4231069	-0.7756961	-0.1410356

Table S14. Association of rs1617640 and traits passing genome-wide significance (P < 5E08) obtained from a PheWAS on 869 traits in up to 451,099 European, unrelated, UK Biobank individuals. All reported effect sizes are aligned to the EPO-increasing A allele of rs1617640.

Phenotype	Beta	SE	P-value
Red blood cell count	-0.0643	0.0017	2.3E-315
Corpuscular hemoglobin	0.0721	0.002	3.3E-313
Corpuscular volume	0.0615	0.0019	8.60E-245
Mean corpuscular volume	0.0612	0.002	1.20E-224
Mean corpuscular volume (anemics excluded)	0.0634	0.0021	3.50E-206
Red blood cell distribution width	-0.0429	0.0021	4.50E-103
Red blood cell distribution width (anemics excluded)	-0.0454	0.0022	3.40E-100
Hematocrit percentage	-0.0349	0.0017	4.70E-98
Corpuscular hemoglobin concentration	0.0328	0.002	1.90E-60
Sphered cell volume	0.0308	0.0019	4.60E-60
Hemoglobin concentration	-0.0244	0.0016	4.40E-51
Platelet Count	0.0234	0.0019	6.40E-39
Non-alcoholic fatty acid liver disease fibrosis score	-0.0202	0.0019	2.20E-25
Platelet volume	-0.0178	0.0018	4.80E-25
Platelet crit	0.0165	0.0019	1.50E-19
Fibrosis-4 score	-0.0141	0.0017	4.70E-17
Reticulocyte volume	0.0152	0.002	3.30E-15
Reticulocyte percentage	0.013	0.002	2.70E-11
Platelet distribution width	-0.0124	0.0019	3.50E-11
High light scatter reticulocyte percentage	0.0114	0.002	1.10E-08

Supplemental information

Supplemental Materials and Methods

Invecchiare in Chianti (InCHIANTI)

InCHIANTI is a prospective, population-based study of 1,453 individuals aged between 20-102 years (1,156 > 65 years) living in the Chianti region of Tuscany, Italy. Data was collected between 1998 and 2000 and included telephone interviews, medical examinations and blood samples. A detailed description of the study has been described previously[1]. The study was approved by the ethical committee of the Italian National Institute of Research and Care of Aging and complies with the Declaration of Helsinki. All participants received a detailed description of the study purpose and procedures and all signed the informed consent. The present study included 1,210 participants with valid phenotypic and genotypic information.

Baltimore Longitudinal Study of Aging (BLSA)

BLSA is a longitudinal cohort study conducted by the Intramural Research Program of the National Institute of Aging (NIH) which started in 1958[2]. Healthy volunteers aged between 17 and older are enrolled in the study and participate in follow-up assessment visits of health, physical and psychological performance every 2 years. Currently, the study population has over 3,200 active participants. An independent institutional review board approved the BLSA study protocol, and participants provided informed consent for all analyses included in this report. The present study included 458 patients with valid phenotypic and genotypic data.

Prevention of Renal and Vascular ENd-stage Disease (PREVEND)

The PREVEND study[3] is a prospective, observational cohort of 8,592 Groningen inhabitants aged between 28-75 years. The main aim of the study is to assess the long-term impact of elevated urinary albumin levels on cardiac- renal- and peripheral vascular end-stage diseases. Upon enrolment, participants agreed to giving a urine sample and answering a questionnaire and are followed up every 2-3 years for a survey on cardiac-, renal- and peripheral vascular morbidity. The PREVEND study

was approved by the medical ethics committee of the University Medical Center Groningen and conducted in accordance with the guidelines of the Declaration of Helsinki. All participants gave written informed consent. The present study included 2,954 individuals with valid phenotypic and genotypic information.

The Health, Aging and Body Composition Study (HealthABC)

HealthABC is a prospective, longitudinal study of 3,075 individuals aged between 70-79 between 1997 and 1998 living in Memphis, Tennessee or Pittsburgh. 42% of participants were of African-American ancestry and 52% were of Caucasian ancestry. Participants were enrolled in the study if they had no disabilities, no life-threatening conditions or difficulties walking quarter of a mile and climbing 10 steps. The study consisted of yearly clinical examinations for 6 years, primarily taking measurements of body composition, strength and function, and biannual phone calls to update health status, followed by bi-annual telephone interviews up until Year 16 and examination in Year 16[4]. All respondents provided written informed consent, and all protocols were approved by the institutional review boards at the study sites. The present study included 1,505 individuals with valid phenotypic and genotypic information.

UK Biobank (UKB) Cohort

Briefly, UKB recruited more than 500,000 individuals aged 37-73 years between 2006 and 2010 from across the UK[5]. Participants provided a range of information via questionnaires and interviews (e.g. health status, lifestyle) and measurements (anthropometric, blood pressure); this has been described in detail by Sudlow et al[6]. SNP genotypes were generated from the Affymetrix Axiom UK Biobank array and the UK BiLEVE array and underwent extensive central quality control (http://biobank.ctsu.ox.ac.uk). We based our analysis on 451,099 individuals of European descent as defined by principal-component analysis (PCA)[7]. We removed 111 participants who withdrew from the study and 348 individuals whose self-reported sex did not match their genetic sex on the basis of relative intensities of X and Y chromosome SNP probe intensity. Genotype-phenotype associations were generated using BOLT-LMM[8] which uses an LD score regression approach to

account for structure caused by relatedness adjusting for SNP chip type, age, sex and test center. For continuous traits, we inverse normalized phenotypes to account for skewed distributions. The UK Biobank has approval from the North West Multicenter Research Ethics Committee (https://www.ukbiobank.ac.uk/ethics/), and these ethics regulations cover the work in this study. Written informed consent was obtained from all participants.

Generation of the EPO phenotype

To generate the EPO phenotype, we included all individuals with valid genomic data, hemoglobin level data and EPO level data from 4 independent cohorts (InCHIANTI, PREVEND, BLSA, HealthABC). We excluded anemic patients as per the WHO definition (Males: hemoglobin (Hgb) levels<13g/dL, Females: Hgb<12g/dL) and patients with renal dysfunction based on an estimated glomerular filtration rate (eGFR) threshold of 50mL/min/1.73m^2 resulting in a final sample size of 6,127 individuals of European and African American descent (InCHIANTI: N = 1,210, PREVEND: N = 2,954, BLSA: N = 458 and HealthABC: N = 1,505). The standard cut-off for renal dysfunction is 60mL/min/1.73m^2 but as the study cohorts were on average older than the general population (Supplementary Table 10), a lower threshold was used due to lower eGFR rates not being unusual in older populations[9] and values between 50-60mL/min/1.73m^2 remaining within the normal distribution of each cohort (Supplementary Fig. 2). The eGFR was calculated using the Modification of Diet in Renal Disease (MDRD) equation[10]. We regressed EPO measures on sex and age and performed rank inverse normalization on the resulting residuals to account for skewed data.

Imputation and Phasing

We included chromosomes 1-23 and genotype data were reported using NCBI b37 (hg19) coordinates. For Europeans, imputation was carried out to the Haplotype Reference Consortium (HRC) version 1.1 using MiniMac3 (http://genome.sph.umich.edu/wiki/Minimac), whilst for African Americans, imputation was carried out to CAAPA [11]. Phasing was carried out using Eagle version 2.3[11].

Quality control filters post-GWAS

After performing GWAS, quality controls checks were undertaken and any single nucleotide polymorphisms (SNPs) with allele frequencies >4 standard deviations (SDs) or <-4 SDs from the Haplotype Reference Consortium (HRC) allele frequency were excluded[12].

Hepatic eQTL data-set

The three hepatic eQTL datasets, comprising a total of 861 liver samples from individuals of European ancestry were analyzed in a meta-analysis (methods and results have been reported in Etheridge et al.[13]. Tissue procurement, genotyping, and gene expression and eQTL analyses have been described previously for each of the three studies[14–16]. Genotypes were imputed to the 1000 Genomes Project Phase 1 reference panel with Minimac (http://genome.sph.umich.edu/wiki/Minimac) and expression probe sequences were mapped to ENSEMBL genes. Within each dataset, a genome-wide eQTL analysis was run with an additive genetic model including dataset specific covariates to examine *cis*-associations within a 1mb flanking window. Results from the three datasets were combined with a modified meta test-statistic which was calculated using the following approach: $t_{meta}=(\sum w_i t_i)/\sqrt{(\sum w_i^2)}$, $w=\sqrt{(n-(\#covariates)-1)}$ where i=datasets 1-3 and n=sample size. Generation of *P*-values was accomplished by assuming the meta test-statistics were normally distributed.

Renal eQTL data-set

The TransplantLines eQTL cohort used for the kidney analysis is part of a donor cohort for which gene expression results have been described previously[17]. The dataset includes kidneys from living donors, donated after brain death and donated after cardiac death (non-heart-beating). Time of biopsy (that is, before transplantation, before reperfusion and after reperfusion) was recorded as well. For some donors, multiple biopsies from different time points were taken. In addition, for some donors biopsies from both kidneys were available. Samples were genotyped on the Illumina CytoSNP 12 v2 array and imputed using the 1000Genomes Phase 1

ALL reference panel[18] using Impute2[19,20]. Expression and genotype data were available for 236 kidney biopsies of 134 donors. A mixed model eQTL analysis adjusting for sex, age, donor type, time of biopsy, first three principle components and sample ID, was run to account for multiple samples from a donor.

Construction of the CRISPR-Cas9 expression vectors

Guide RNA (gRNA) sequences were identified using the online CRISPR design tools (available at https://www.benchling.com/crispr/) by screening the exonic regions conserved across *EPO* transcripts. This online tool uses the latest algorithms to assess the efficiency of the gRNA. Sequences with high predicted off-target scores (>60) and high predicted on-target scores (> 50) were chosen. Overhangs for cloning into a BbsI restriction cut-site were placed on the ends of the gRNAs to enable successful cloning into the CRISPR-Cas9 vector. The gRNAs were ordered from Integrated DNA technologies (IDT) (https://eu.idtdna.com/). The gRNAs were ligated into the CRISPR plasmids in a single digestion and ligation reaction using T4 ligase (New England BioLabs, Ipswich, UK) following manufacturer's protocol. We transformed bacteria (*Escherichia Coli* DH5-alpha) with ligated plasmids before purifying the plasmid DNA following the QIAprep Spin Miniprep Kit protocol (Qiagen, Maryland, USA). A double diagnostic restriction digest with BbsI and EcoRI was used to confirm successful ligation of the gRNA into the plasmid (Extended Data Fig. 2B). Plasmids with the correct digestion pattern were sent for Sanger sequencing with the LKO.1 forward primer (5'-GACTATCATATGCTTACCGT-3') to confirm insertion of the gRNA in the correct orientation and location (Extended Data Fig. 2C).

Whole transcriptomic Analysis

Library preparation was performed using the TruSeq DNA HT Library Preparation Kit using the 3' poly-A tail primer Oligo(dT) from Illumina (Illumina, California, USA). RNA Sequencing was performed using the Illumina HiSeq 2500 high-throughput sequencing system (Illumina, California, USA). We resulted in 75 bp paired-end sequences.

Construction of piggyBac™ expression vector

We designed 500 bp homology arms complementary to the genomic DNA either side of the TTAA site closest (88 bp) to the desired SNP edit (rs1617640 at position 7:100317298). In order to ensure seamless excision of the *piggyBac*™ transposon from the genomic DNA after transposase treatment, we inserted the homology arms into the *piggyBac*™ multivector (SGK:005, MV-PGK-Puro-TK) (Hera BioLabs, Kentucky, USA) at the BsiW1 (for insertion of 5' homology arm) and Nsi1 (for insertion of 3' homology arm) restriction sites using the Gibson Assembly Cloning Kit (New England BioLabs, Ipswich, UK). We therefore had to add the remainder of the 3'ITR region to the 5' homology arm and the remainder of the 5'ITR region to the 3' homology arm. For Gibson cloning to work, 20 bp of sequence complementary to the *piggyBac*™ vector either side of the cut site was also added to the either end of the homology arm sequence. Final homology sequences were ordered as MiniGenes (Integrated DNA Technologies) and then amplified out of the holding vector via PCR. Ordered homology sequences can be seen below with red depicting the desired SNP change at rs1617640, blue depicting the TTAA site, bold depicting the remainder of the 3'ITR region (for the 5'homology arms) or the 5'ITR region (for the 3' homology arm) and the underlined bases depicting the 20 bp sequences complementary to the *piggyBac* multivector sequence (SGK:005, MV-PGK-Puro-TK).

5’ homology arm sequence for wild-type (WT) sequence at rs1617640 (A/A)

ATAATCATATTGTGACGTACTATTTATTTATTTATTTTAGAGACAAGGTCTTGCCATGTTGT
CCGGGCTGGTCTCGAACTCCTGGGCTCAAAGGATCTTCCTGCCTTGGTCTCCCAAAGTGCT
GGGATTATAGGTGTCAGCTGCGGCGCCTGGACCTTTCCTGTCTTTTATGAAACCTGAATGG
GATAGGCTGGTAGTTTCACCACACCCATTTGACAGATGAGGACATTGAGGGCTCAAGGAC
GAGGCCACTTTCTAAGGTGTGAGAGACCAGCTAGTCTTGGTCTCCTGCTCTGGGAATCTCA
CTCATCTGGCTCAGGGTTTCCAGAAGCCATAAAACCTTAGCTGTAAATCCCAGCCCCCAT
CACTCTTGGTGTTAGCTGTATTTCAGTGTTCTTAA**CCCTAGAAAGATAATCATATTGTG**
ACGTACGTTAAAGATAATCATG

5’ homology arm sequence for SNP change at rs1617640 (C/C)

ATAATCATATTGTGACGTACTATTTATTTATTTATTTTAGAGACAAGGTCTTGCCATGTTGT
CCGGGCTGGTCTCGAACTCCTGGGCTCAAAGGATCTTCCTGCCTTGGTCTCCCAAAGTGCT
GGGATTATAGGTGTCAGCTGCGGCGCCTGGACCTTTCCTGTCTTTTATGAAACCTGAATGG
GATAGGCTGGTAGTTTCACCACACCCATTTGACAGATGAGGACATTGAGGGCTCAAGGAC
GAGGCCACTTTCTAAGGTGTGAGAGACCAGCTAGTCTTGGTCTCCTGCTCTGGGAATCTCA
CTCCTCTGGCTCAGGGTTTCCAGAAGCCATAAAACCTTAGCTGTAAATCCCAGCCCCCAT
CACTCTTGGTGTTAGCTGTATTTCAGTGTTC**TTAACCCTAGAAAGATAATCATATTGT**
GACGTACGTTAAAGATAATCATG

3’ homology arm sequence

AGCAATATTTCAAGAATGCA**TGCGTCAATTTTACGCAGACTATCTTTCTAGGGTTAA**
GAACTCAGCAATGCAGCCTAGCTAACCTACACCACAGGTCAAATAAACAGATGTCAAGGT
GCATGTGTGTCCTGCACAATGGACTGTGTGCTCTGTGCACTAAAAGTTAAGTGTCTGGGGT
GGGGGCTGGGTGCGGTGGCTCACGCCTGCAATCCCAGCACTTTGAGAGGCCGAGGAGGG
TGGATCACCTGAGGTCAGGAGTTCAAGACCGGTATGGGCAATATGGCAACACCCCATCTC
TACTAAAAATACAAAACATAGCCGGGTGTAAAGGTGTGCCTGTAGTCCCAGCTGCTCCAG
AGGCTGAAGCAGGAGAATTGCTTGAACCCAGGAGGCAGAGGTTGTAGTGAACGGGAGAT
GGCACCACTGCACTGCCTGGGCAACATAGGGAGGCTACATCTCCAGAAAAAAAAAAAAA
AGTTATGCATGCGTCAATTTTACGC

Gibson cloning was first performed for insertion of the 5’ homology arm into the *piggyBac*^{TM} plasmid after digestion with the BsiW1 restriction enzyme. Ligated plasmids were transformed into bacteria (*Escherichia Coli* DH5-alpha). Plasmids were purified from overnight cultures using the QIAprep Spin Miniprep Kit protocol (Qiagen, Maryland, USA). We confirmed successful insertion of the 5’ homology arm

using PCR (Extended Data Figs. 5A-B). Successful plasmids containing the 5' homology arm was digested with Nsi1 and Gibson Cloning was repeated to insert the 3' homology arm into the *piggyBac*^{TM} plasmid following manufacturer's protocol (New England BioLabs, Ipswich, UK). We transformed the ligated plasmids into competent bacteria and purified plasmid DNA from overnight cultures using the QIAprep Spin Miniprep Kit protocol (Qiagen, Maryland, USA) We confirmed successful cloning of both arms into the plasmid using a single diagnostic digest (Extended Data Fig. 5C). Sanger sequencing was then performed on final constructs to confirm correct insertion of both homology arms into the *piggyBac*^{TM} plasmid (Extended Data Figs. 5D-F).

Supplemental References

1. Ferrucci, L. *et al.* Subsystems contributing to the decline in ability to walk: bridging the gap between epidemiology and geriatric practice in the InCHIANTI study. *J. Am. Geriatr. Soc.* **48**, 1618–1625 (2000).
2. Shock, N. W. Normal human aging: The Baltimore longitudinal study of aging. (1984).
3. Pinto-Sietsma, S. *et al.* Urinary Albumin Excretion Is Associated with Renal Functional Abnormalities in a Nondiabetic Population. *J. Am. Soc. Nephrol.* **11**, 1882 LP – 1888 (2000).
4. Simonsick, E. M. *et al.* Measuring Higher Level Physical Function in Well-Functioning Older Adults: Expanding Familiar Approaches in the Health ABC Study. *Journals Gerontol. Ser. A* **56**, M644–M649 (2001).
5. Collins, R. What makes UK Biobank special? *Lancet (London, England)* **379**, 1173–1174 (2012).
6. Sudlow, C. *et al.* UK Biobank: An Open Access Resource for Identifying the Causes of a Wide Range of Complex Diseases of Middle and Old Age. *PLOS Med.* **12**, e1001779 (2015).
7. Frayling, T. M. *et al.* A Common Allele in FGF21 Associated with Sugar Intake Is Associated with Body Shape, Lower Total Body-Fat Percentage, and Higher Blood Pressure. *Cell Rep.* **23**, 327–336 (2018).
8. Loh, P.-R. *et al.* Efficient Bayesian mixed-model analysis increases association power in large cohorts. *Nat. Genet.* **47**, 284–290 (2015).
9. Wetzels, J. F. M., Kiemeney, L. A. L. M., Swinkels, D. W., Willems, H. L. & Heijer, M. de. Age- and gender-specific reference values of estimated GFR in Caucasians: The Nijmegen Biomedical Study. *Kidney Int.* **72**, 632–637 (2007).
10. Levey, A. S. *et al.* Expressing the Modification of Diet in Renal Disease Study equation for estimating glomerular filtration rate with standardized serum creatinine values. *Clin. Chem.* **53**, 766–772 (2007).
11. Das, S. *et al.* Next-generation genotype imputation service and methods. *Nat. Genet.* **48**, 1284–1287 (2016).
12. McCarthy, S. *et al.* A reference panel of 64,976 haplotypes for genotype imputation. *Nat. Genet.* **48**, 1279–1283 (2016).
13. Etheridge, A. S. *et al.* A New Liver Expression Quantitative Trait Locus Map From 1,183 Individuals Provides Evidence for Novel Expression Quantitative Trait Loci of Drug Response, Metabolic, and Sex-Biased Phenotypes. *Clin. Pharmacol. Ther.* **107**, 1383–1393 (2020).

14. Innocenti, F. *et al.* Identification, replication, and functional fine-mapping of expression quantitative trait loci in primary human liver tissue. *PLoS Genet.* **7**, e1002078 (2011).

15. Schadt, E. E. *et al.* Mapping the genetic architecture of gene expression in human liver. *PLoS Biol.* **6**, e107 (2008).

16. Greenawalt, D. M. *et al.* A survey of the genetics of stomach, liver, and adipose gene expression from a morbidly obese cohort. *Genome Res.* **21**, 1008–1016 (2011).

17. Damman, J. *et al.* Hypoxia and complement-and-coagulation pathways in the deceased organ donor as the major target for intervention to improve renal allograft outcome. *Transplantation* **99**, 1293–1300 (2015).

18. Auton, A. *et al.* A global reference for human genetic variation. *Nature* **526**, 68–74 (2015).

19. Howie, B. N., Donnelly, P. & Marchini, J. A Flexible and Accurate Genotype Imputation Method for the Next Generation of Genome-Wide Association Studies. *PLOS Genet.* **5**, e1000529 (2009).

20. Howie, B., Marchini, J. & Stephens, M. Genotype Imputation with Thousands of Genomes. *G3 Genes, Genomes, Genet.* **1**, 457–470 (2011).
